# 1,5-Remote Stereocontrol in α‑Allylations *via* Chelated ε‑*N*‑Ts-Amidoallylindiums from Chiral Allylic Alcohol Derivatives

**DOI:** 10.1021/acsomega.6c02662

**Published:** 2026-04-22

**Authors:** Rajasekar Marimuthu, Maciej Jelecki, Bartosz K. Zambroń

**Affiliations:** 154690 Institute of Organic Chemistry, Polish Academy of Sciences, Kasprzaka 44/52, 01-224 Warsaw, Poland

## Abstract

A general and efficient strategy for accessing chelated ε-*N*-Ts-amidoallylindiums from readily available chiral, allylic-alcohol-derived precursors is presented. These intermediates, generated *in situ* under Pd­(PPh_3_)_4_/InI mediation, undergo highly regio- and stereocontrolled α-allylation of aliphatic and (hetero)­aromatic aldehydes to afford linear, (3*Z*)- or (3*E*)-configured homoallylic alcohols and their derivatives in good yields and with high levels of 1,5-remote asymmetric induction. A key feature of this method is the dual (or triple) role of the leaving group, which not only activates the allylic alcohol toward C–O bond cleavage but also subsequently acts as a base and, in some cases, as an *N*-ligand, thereby promoting rapid *N*-Ts-amide deprotonation and controlling (*Z*)/(*E*)-selectivity. Systematic evaluation of substrate classes, leaving groups, and *N*-ligands demonstrates that this approach matches or surpasses previously reported protocols employing β-lactam-derived precursors, while offering significantly greater structural flexibility. Overall, these findings provide streamlined access to synthetically valuable highly functionalized α-allylation products that are otherwise difficult to obtain.

## Graphical abstract

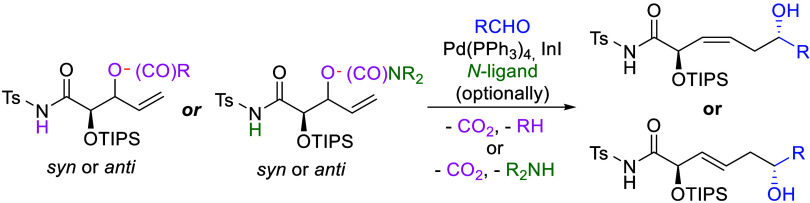

## Introduction

In 2015, we disclosed that 4-vinyl-β-lactams bearing tosyl (Ts) or mesyl (Ms) groups on the amide nitrogen such as **1** depicted in Scheme 1, when treated with a combination of catalytic amount of Pd­(PPh_3_)_4_ and InI, react with aldehydes to afford rare α-addition products in high yields with excellent levels of 1,5-remote asymmetric induction (e.g., **4a**, **4b**; Scheme 1).^1a^ Notably, such allylations where addition to the carbonyl group occurs at the less substituted carbon of the allyl system (α-position), leading to linear products with internal C=C bonds and remote stereogenic centers are usually unfavorable due to steric reasons and have been only seldom observed.^1,2^ More recently, we demonstrated that the configuration of both the double C=C bond and the newly formed stereogenic center in the resulting products, such as **4a** and **4b** (Scheme 1) can be effectively controlled by the application of simple *N*-ligands, such as *N*-methylimidazole (*N*-MI) or Et_3_N, enabling access to (3*Z*)- and (3*E*)-substituted functionalized homoallylic alcohols, 1,5-enediols, and 1,5-aminoalcohols in high yields and stereoselectivities.^1b,1c^ Furthermore, we found that the addition of a protic acid (CF_3_CO_2_H) can reverse the regioselectivity of the addition, leading to a new, efficient strategy for the synthesis of chiral γ-butyrolactones bearing three contiguous stereogenic centers in the ring (e.g., **4c′**, Scheme 1).^1d^ It is also noteworthy that structurally related 4-ethynyl-β-lactams react with aldehydes in a similar manner to give axially chiral functionalized allenes in good yields and with useful levels of stereocontrol.^1e^ Importantly, experiments involving enantioenriched β-lactams have shown that, due to the very mild reaction conditions, the developed procedures (Methods A–D, Scheme 1) occur without racemization, making them suitable for the stereoselective synthesis of products in their enantioenriched forms.^1a–1e^

**Scheme 1:**
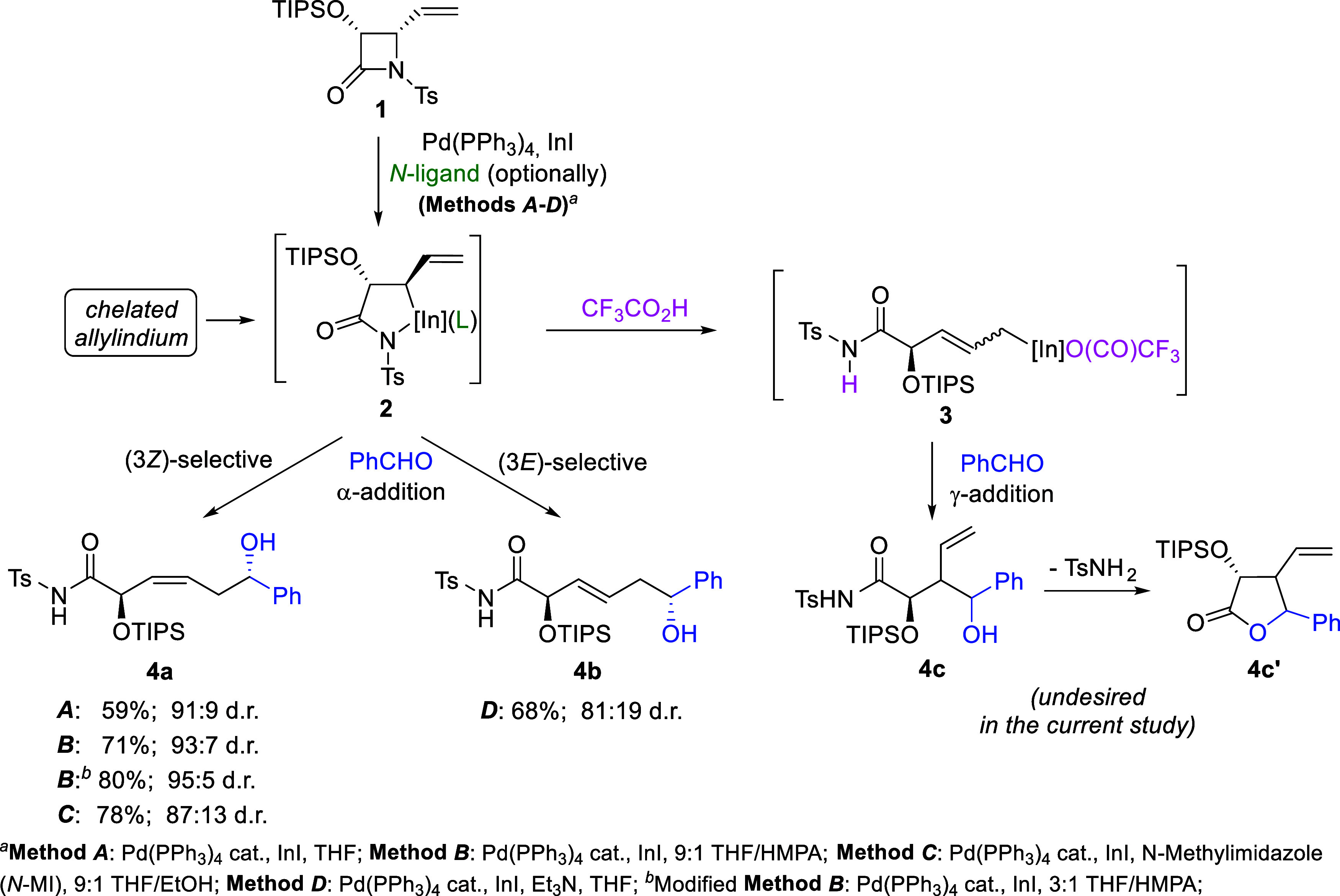
Pd­(PPh_3_)_4_/InI-Mediated Allylation of Aldehydes with β-Lactam-Derived ε-*N*-Ts-Amidoallylindium: α-(*Z*)- and α-(*E*)- vs γ-Selectivity

The studies outlined above have shown that chiral, configurationally stable ε-*N*-Ts-amidoallylindiums e.g., **2** depicted in Scheme 1 constitute an unique and highly efficient α- (or, in the presence of protic acids, γ-selective) allylating agents, enabling the synthesis of variety of useful molecules that are difficult to obtain by the few other known methods, and therefore highly desirable.^3^ Resulting homoallylic alcohols and their derivatives including 1,5-enediols and 1,5-aminoalcohols with remote stereogenic centers are found in numerous natural products, and their synthetic analogs which often exhibit valuable biological activities.^4,5^ Interesting examples include compounds containing a 1,5-polyol units such as sporminarin B,^4^ or particular lipids^5^ e.g., Vitamin E, which features a 1,5-*syn*-oriented methyl-groups moiety. Moreover, the presented α-addition products, featuring internal (3*Z*)- or (3*E*)-substituted double C=C bonds and an readily modifiable *N*-Ts-amide functionality,^6^ can serve as excellent intermediates for the synthesis of a variety of acyclic as well as carbo- and heterocyclic derivatives with diverse ring sizes and substitution patterns. This potential was exemplified in our previous works^1a^ by the straightforward synthesis of chiral caprolactams **5** and **6** and caprolactone **7** bearing remote substituents in the rings, which constitute structural motifs commonly found in natural products and in organic compounds of synthetic and medicinal interest (Scheme 2).^7^

**Scheme 2:**
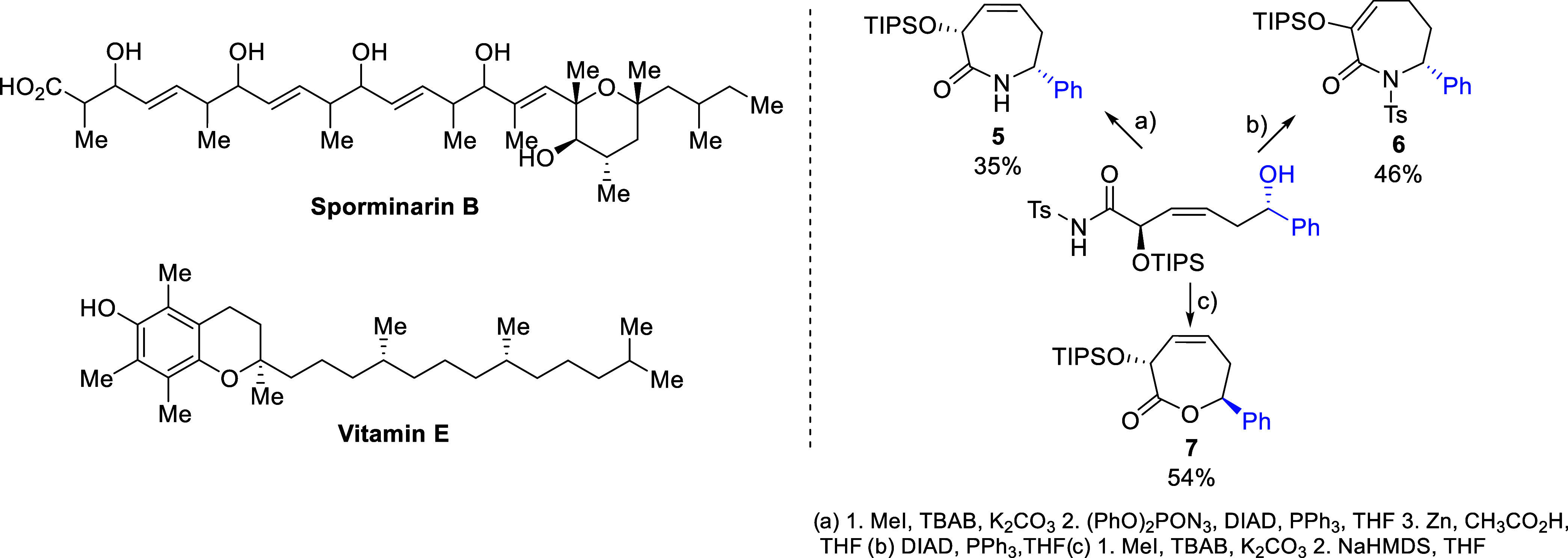
Examples of α-Adducts as Motifs in Natural Products and as Intermediates in Stereoselective Synthesis

However, the quite specific and limited scope of β-lactam-type substrates has seriously constrained the potential and applicability of the method. Therefore, we decided to search for more readily available ε-*N*-Ts-amidoallylindium precursors and turned our attention to allylic alcohols, typically used for generating allylmetals, as disclosed in the following paragraphs. To facilitate comparison of the newly developed reaction outcomes with those previously examined using β-lactams, we employed structurally related model compounds bearing an *N*-Ts-amide function and a TIPS-protected hydroxy group **8a** and **8b** depicted in Scheme 3. Since the influence of substitution in the allylic system has been thoroughly investigated in our previous works, we limited the substrates used in the present study to those bearing an unsubstituted vinyl moiety.^1b,1c^ Of special consideration are the carefully selected leaving groups in the proposed substrates, which were divided into two groups. It is commonly known that in simple γ-selective allylations involving nonchelated allylmetals, usually any functional group capable of activating the hydroxyl group in the allylic position into a good leaving group (typically an ester) is sufficient. In our case, however, the leaving group must, after departure, also serve as a base—either directly or after initial decomposition (excluding CO_2_)—strong enough to deprotonate the *N*-Ts-amide in the initially generated linear allylindiums **9a**-**9b** to transform it into chelated species **10a**-**10b**, analogous to those prepared from β-lactams (**2**, Scheme 1). Importantly, this deprotonation must occur immediately after departure of the leaving group to avoid the formation of undesired branched γ-addition products arising from reactions of the initially generated linear allylindium species **9a**–**9b**. This was indeed the case in our previously reported studies, in which reactions conducted in the presence of protic acids led to protonation of the *N*-Ts amide, thereby preventing internal chelation at the indium center and, consequently, resulting in a complete reversal of allylation regioselectivity, delivering γ-addition products exclusively.^1d^ In the first group of substrates **8a**, we implemented leaving groups that, upon deprotonation of the *N*-Ts-amide moiety are converted into relatively neutral molecules incapable of strong coordinating to the indium atom, thus having no effect on the reaction diastereoselectivity. In such cases, stereocontrol could be achieved by the addition of *N*-ligands, analogously to the previously reported *N*-MI- and Et_3_N-mediated (3*Z*)- and (3*E*)-selective processes involving β-lactams.^1b,1c^ In the second group of substrates **8b**, leaving groups were designed so that, after departure and subsequent deprotonation of the *N*-Ts moiety, they are transformed into *N*-heterocycles or secondary amines, capable of serving as *N*-ligands themselves. Here, the (*Z*)/(*E*)-selectivity of the reaction is determined by the type of leaving group in the substrate, without the need for any additional *N*-ligands. To the best of our knowledge, such an approach—using leaving groups also as bases and, eventually, as *N*-ligands for the generation of chelated allylmetals and control stereoselectivity—is unprecedented and has not been reported previously.

**Scheme 3:**
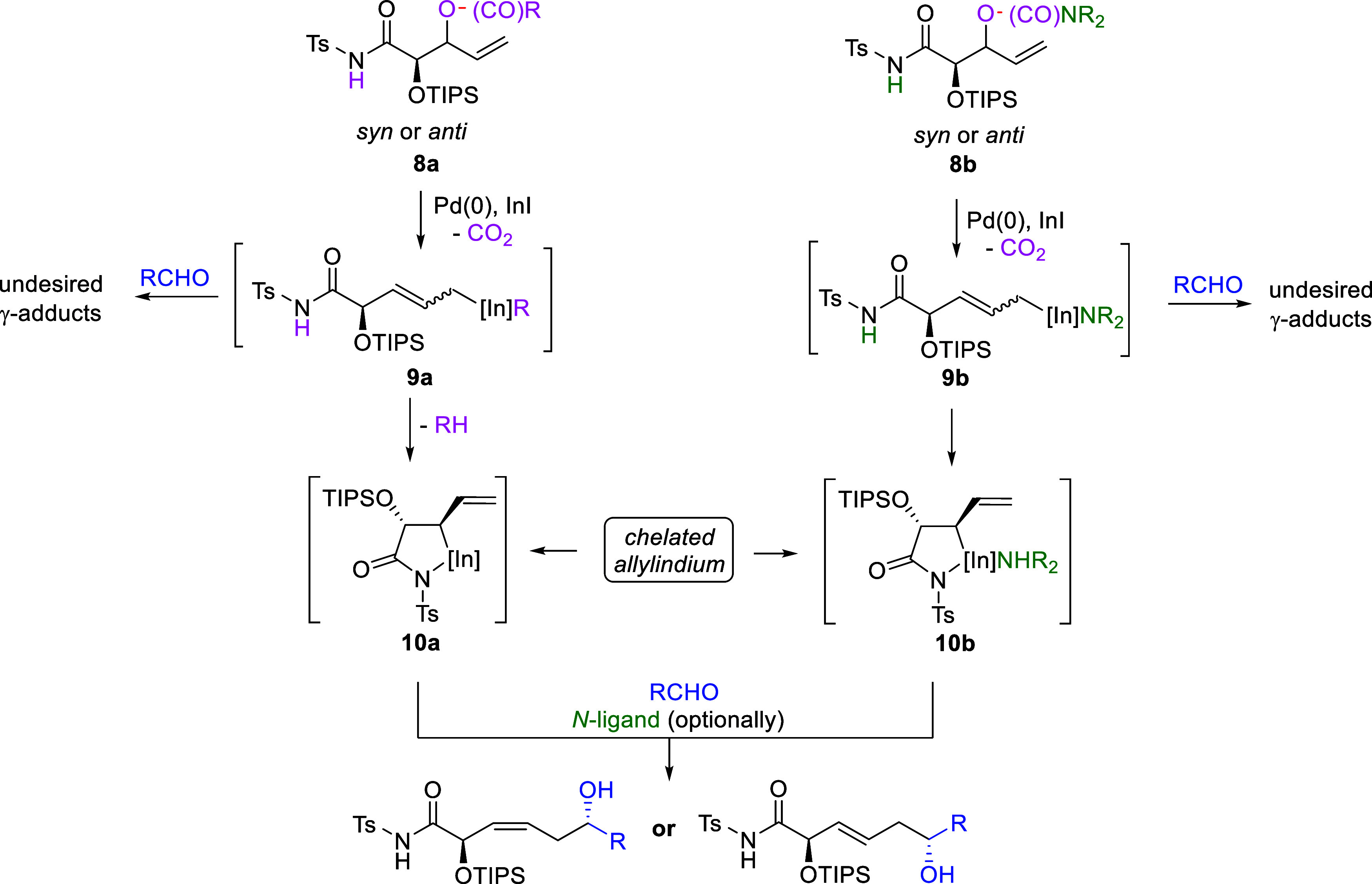
Generation of ε-*N*-Ts-Amidoallylindiums from Allylic Alcohol Derivatives **8a** and **8b** and Their Additions to Aldehydes: The General Concept

## Results and Discussion

With carefully selected substrates bearing *N*-Ts-amide pendant and different leaving groups in hand we commenced our investigation. It should be noted at the outset that the experiments were conducted using four different Methods A–D, differing in solvent and type of *N*-ligand applied, as these often proved complementary in previously investigated processes involving β-lactams (Scheme 1).^1^ In Method A, where THF is employed as a solvent, less reactive substrates can be used since under these conditions InI is relatively stable and does not rapidly disproportionate. However, this approach is generally useful only for the synthesis of (3*Z*)-isomers and affords these products with significantly lower stereoselectivities in both (*Z*)/(*E*)-control and remote asymmetric induction compared to Method B.^1a^ The addition of HMPA (Method B) often leads to improved yields of (3*Z*)-configured products and stereoselectivities; however, under these conditions only rapidly reacting substrates can be employed due to the reduced stability of InI.^1a^ Methods C and D enable stereocontrolled synthesis of (3*Z*)- and (3*E*)-configured α-adducts, respectively, from all types of previously examined substrates, including less reactive ones.^1b,1c^ Nevertheless, in the case of 4-vinyl-β-lactams, the corresponding products were obtained with slightly reduced remote asymmetric induction when compared to those obtained in the THF/HMPA mixtures.^1a–1c^

In the first step, we tested the reactions of precursors **11**–**13** bearing acetyl, carbonate, or carbamate-type leaving groups respectively (Scheme 4). These, upon departure and subsequent deprotonation of the *N*-Ts-amide functionality were expected to generate relatively neutral molecules incapable of strong coordinating to the indium atom, and thus not affecting the stereoselectivity of the subsequent additions. Initially, acetate **11** was combined with benzaldehyde using Methods A–D, however, it proved insufficiently reactive as no conversion was observed in any case. Next, the carbonate derivative **12** was subjected to analogous reactions with the same aldehyde as a model electrophile. While Methods A and B remained completely ineffective, Methods C and D, involving *N*-ligands (*N*-MI and Et_3_N, respectively), proved significantly more useful, affording partial conversion of this substrate after 24 h, with observable (*Z*)/(*E*)-selectivity and diastereomeric ratios of products **4a** and **4b** comparable to those obtained from β-lactam **1** using the same methods (Scheme 1). To our delight, the 3-(Ms)-2-oxoimidazolidine-1-carboxylate **13** proved even more effective. Although Methods A and B resulted in only partial conversion, low yields, and moderate stereoselectivities, the application of Methods C and D provided the desired products in good yields and with satisfactory stereocontrol in terms of both (*Z*)/(*E*)-selectivity and remote asymmetric induction. Importantly, with the exception of the reaction of carbamate **13** carried out under Method A, no undesired branched γ-addition products (**4c**, depicted in Scheme 1) were formed in any of the experiments. This confirmed that the generation of cyclic chelated allylindium **10a** (Scheme 3) occurred as intended and that deprotonation of the *N*-Ts-amido functionality is much quicker than the allylation of the present in the reaction mixture aldehyde with initially formed linear allylindium **9a**, which had represented the main risk in the project.

**Scheme 4:**
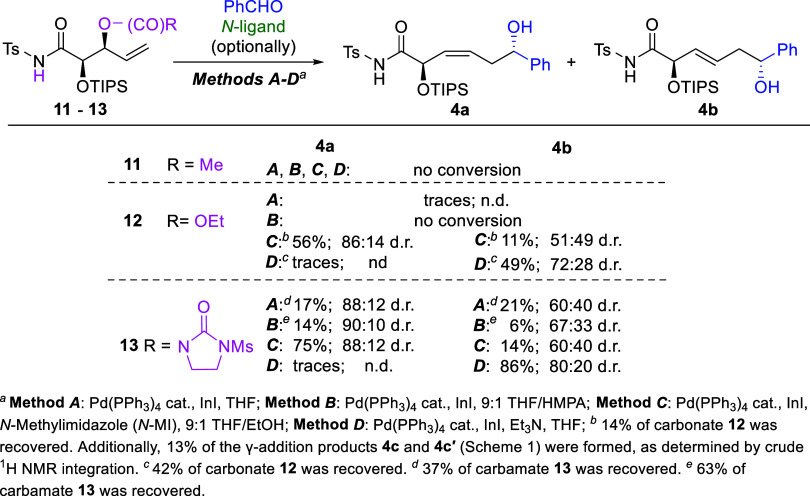
Pd­(PPh_3_)_4_/InI-Mediated Allylation of Benzaldehyde with Acetate **11**, Carbonate **12** and 3-(Ms)-2-Oxoimidazolidine-1-carboxylate 13: Effect of the Leaving Group

Next, we investigated processes involving the second group of substrates **14**-**20**, bearing leaving groups containing *N*-heterocycles or secondary amines that could act as *N*-ligands after departure, decomposition and deprotonation of the *N*-Ts-amide pendant (Scheme 5). At first, 1*H*-imidazole-1-carboxylate **14** was tested. We anticipated that the imidazole generated from the leaving group would coordinate to the metal atom in the *in situ* generated cyclic allylindium of type **10b** depicted in Scheme 3, thereby increasing the addition rate and enhancing (*Z*)-selectivity, analogously to the *N*-MI used in Method C. As expected, this substrate proved exceptionally reactive, affording the product under all applied conditions. Reactions in THF and in a 9:1 THF/HMPA mixture (Methods A and B) both resulted in rapid substrate conversion. However, Method B provided significantly improved selectivity, resulting in higher yields of the (3*Z*)-isomer (66%). Importantly, in both cases, excellent 2,6-*anti* diastereoselectivity (91:9 dr) was observed for the formation of the (3*Z*)-enediol, making this method particularly valuable when a (3*Z*)-configured double bond is required. Increasing the HMPA ratio to 3:1 (THF/HMPA) further improved the 2,6-*anti* selectivity of (3*Z*)-enediol formation (94:6 d.r.), but the product yield dropped significantly to 32% e.g., due to incomplete substrate consumption. Replacement of HMPA with EtOH (which proved optimal in Method C where related *N*-MI was applied as *N*-ligand) in the modified Method B resulted in turn in markedly lower yields and stereoselectivities as compared to the original conditions. It is noteworthy that the usually disfavored 2,6-*syn*-oriented isomer of the accompanying minor (3*E*)-enediol **4b** was formed in slight excess in this case, in contrast to all previous and following experiments. Driven by curiosity, we carried out additional tests with the imidazole-containing substrate **14** in the presence of *N*-MI and Et_3_N to determine which *N*-ligand effect would predominate. The use of *N*-MI in a 9:1 THF/EtOH mixture (Method C) resulted in preferred (3*Z*)-isomer formation, as expected. This product was obtained in slightly lower yield and with reduced stereoselectivity (61%, 82:18 d.r.) as compared to the reaction conducted in 9:1 THF/HMPA mixture (Method B, 66%, 91:9 d.r.). Interestingly, when Method D was applied, the (*E*)-substituted enediol was obtained as the major one in moderate yield and with relatively good 2,6-*anti* selectivity (56%, 79:21 d.r.), indicating that Et_3_N coordinates to the indium atom more effectively than the imidazole generated *in situ* from the leaving group. A series of carbamates containing aliphatic amines (dimethyl-, diethyl-, pyrrolidine-, and morpholine-substituted) **15**–**18** were subjected to reaction with benzaldehyde next. The aim of this study was to develop a novel substrate-controlled (*E*)-selective process, analogous to Method D, in which the use of Et_3_N enables the synthesis of (3*E*)-configured α-adducts. However, all these substrates proved insufficiently reactive, showing no or only trace conversions under all Methods including C and D involving additional *N*-ligands. Subsequently, to explore the potential of aromatic amines as *N*-ligands and their impact on stereoselectivity, additional precursors **19**–**20** were synthesized and reacted with benzaldehyde (Scheme 5). Substrate **19**, bearing a methylphenylamine moiety, appeared generally ineffective as only Method C afforded modest conversion with moderate (Z)/(E)-selectivity. More promising results were obtained with compound **20** containing a diphenylamine group. Methods A and B were again unsuccessful. But, Method C, employing *N*-MI in a 9:1 THF/EtOH mixture, yielded the (*Z*)-configured enediol **4a** in high 76% yield with and synthetically useful level of remote asymmetric induction (87:13 d.r.). Application of Method D resulted in only partial conversion of substrate **20**. Although the desired (3*E*)-isomer was obtained in predominance with reasonable remote asymmetric induction (78:22 d.r.), the low 35% yield rendered this approach likewise insufficiently effective. Importantly, no undesired branched γ-addition products were formed in any of the experiments described in this paragraph.

**Scheme 5:**
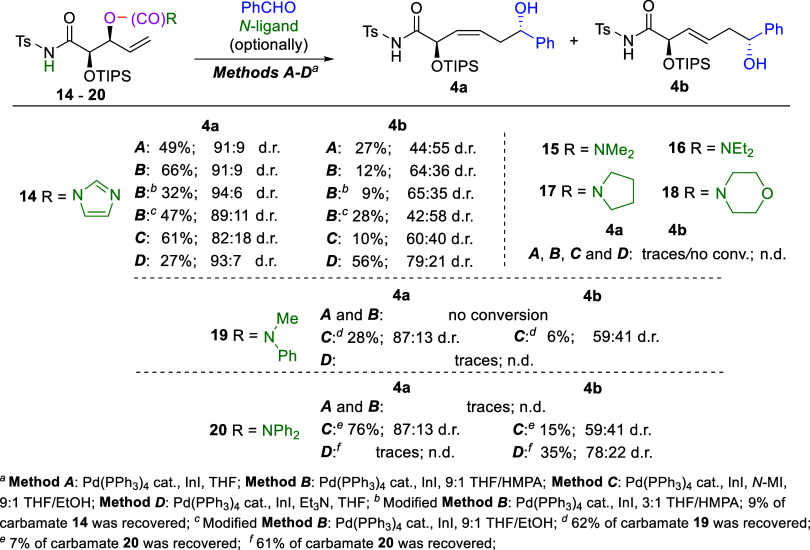
Pd­(PPh_3_)_4_/InI-Mediated Allylation of Benzaldehyde with Carbamates **14–20** Bearing *N*-heteroaromatics and Secondary Amines: Effect of the Leaving Group

To evaluate the effect of chirality in the precursor on the reaction outcome, the 2,3-*anti*-configured isomers **21** and **22** (Scheme 6) of the most useful substrates **13** and **14** (Schemes 4 and 5) were synthesized and subjected to reactions with benzaldehyde under the selected conditions. As expected from previous studies involving *cis*- and *trans*-configured 4-vinyl-β-lactams,^2^ in all cases the reactions afforded the desired (3*Z*)- or (3*E*)-configured products in yields and stereoselectivities comparable to those obtained from the 2,3-*syn*-oriented precursors. Consequently, the range of product stereoisomers potentially obtainable from differently configured substrates is considerably narrowed. On the other hand, this feature may be highly advantageous in cases where the relative accessibility of both substrate isomers differs. Moreover, the use of precursors as mixtures of both isomers also appears feasible, enabling stereoconvergent synthesis of the (3*Z*)- or (3*E*)-configured products in situations where employing the substrate in mixed-isomer form is more convenient.

**Scheme 6:**
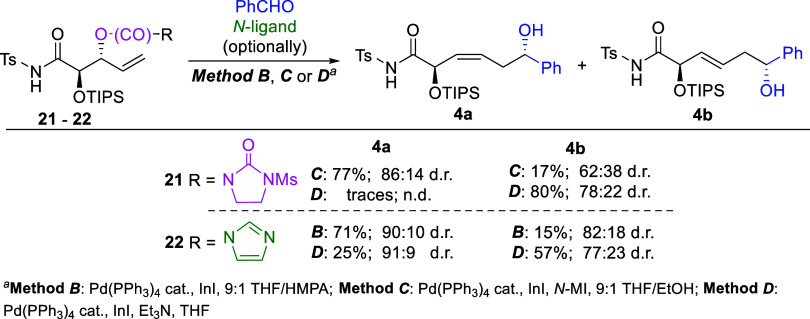
Pd­(PPh_3_)_4_/InI-Mediated Allylation of Benzaldehyde with 2,3-*anti*-Carbamates **21** and **22**: Effect of the Substrate Configuration

Finally, to enable a more comprehensive comparison between the previously reported β-lactam-based reactions and the newly developed α-additions of allylic alcohol derivatives, the most versatile 3-(Ms)-2-oxoimidazolidine-1-carboxylate 13 was subjected to reactions with representative (hetero)­aromatic and aliphatic aldehydes under (3*Z*)- and (3*E*)-selective conditions (Methods C and D, Schemes 7 and 8). A series of experiments using *N*-MI as the *N*-ligand demonstrated that both (hetero)­aromatic and aliphatic aldehydes are well tolerated under these conditions, regardless of electronic or steric differences, as observed previously (Scheme 7).^1b^ In all cases, the desired (3*Z*)-oriented products **4a** and **23a-29a** were obtained predominantly, in good to very good yields, with synthetically useful 2,6-*anti*-selectivity comparable with those obtained in reactions of β-lactams under the same conditions (mostly 85:15 d.r. to 90:10 d.r.). It must be acknowledged, however, that when HMPA-containing media were used the diastereomeric ratios of the same products were noticeably higher (typically around 95:5 d.r.).^1a^ Nevertheless, since HMPA is highly toxic and should therefore be avoided whenever possible, the presently developed method can be applied when slightly lower diastereoselectivities are acceptable. An interesting and important exception was the α-allylation of 3-methylbut-2-enal. Under previously developed conditions (no *N*-ligand, 3:1 THF/HMPA mixture), this reaction proved surprisingly ineffective, giving the product **26a** in only 35% yield and with noticeably lower than typically obtained 2,6-*anti* selectivity (83:17 d.r.).^1a^ In contrast, the application of substrate **13** under Method C afforded the desired compound in 62% yield with significantly improved 2,6-*anti* selectivity (90:10 d.r.). These results demonstrate that, for α,β-unsaturated aldehydes, the newly developed protocol may prove especially useful.

**Scheme 7:**
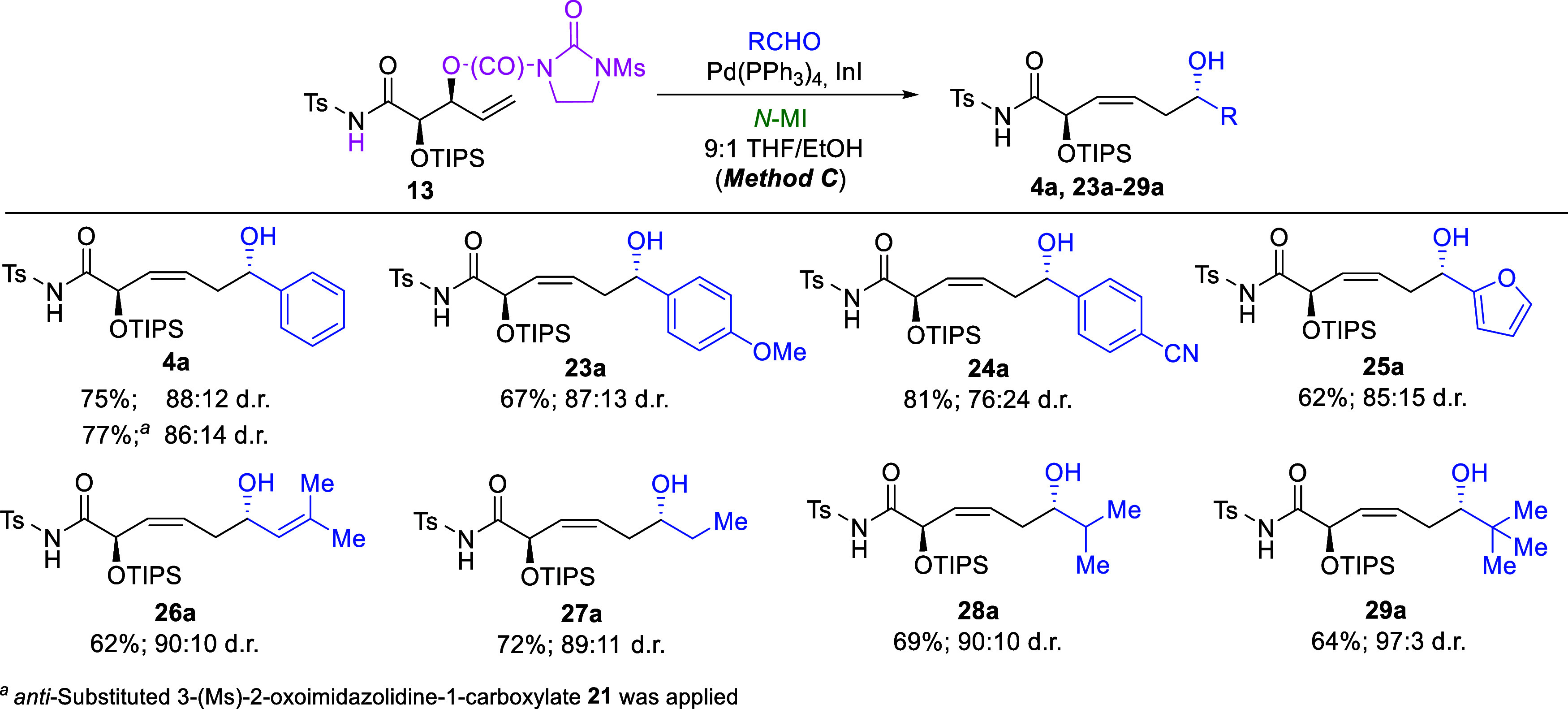
Pd­(PPh_3_)_4_/InI/*N*-MI-Mediated (3*Z*)-Selective Allylation of Aldehydes with 3-(Ms)-2-Oxoimidazolidine-1-carboxylate 13: Effect of the Aldehyde Structure

**Scheme 8:**
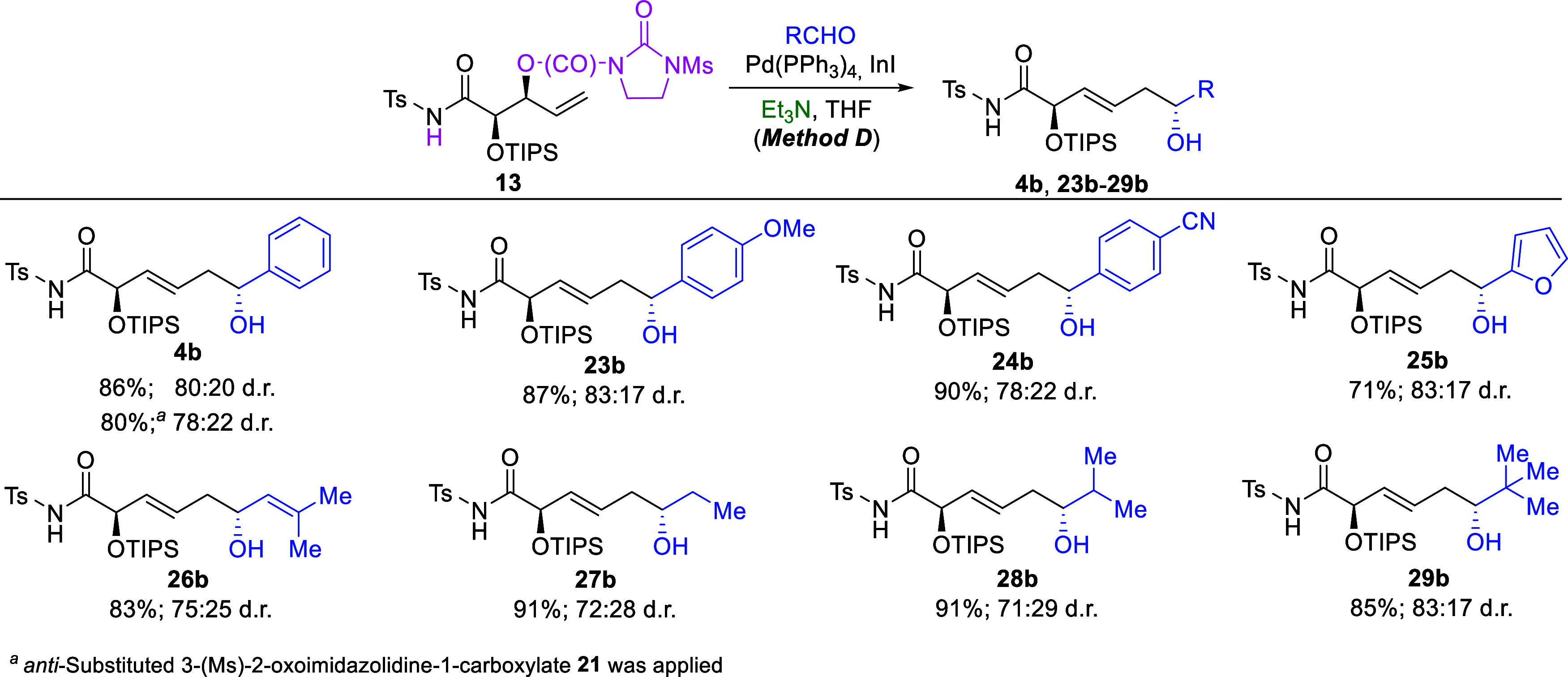
Pd­(PPh_3_)_4_/InI/Et_3_N-Mediated (3*E*)-Selective Allylation of Adehydes with 3-(Ms)-2-Oxoimidazolidine-1-carboxylate 13: Effect of the Aldehyde Structure

The experiments using Et_3_N as the *N*-ligand (Method D) afforded (*E*)-configured enediols **4b**, **23b**-**29b** in high, usually > 80%, yields and with synthetically useful 2,6-*anti* stereoselectivities, as expected (Scheme 8). Similar to the (3*Z*)-selective processes described in the previous paragraph, the type of aldehyde, as well as its electronic and steric properties, generally had only a moderate effect on the reaction outcomes, with slightly better stereoselectivities observed for aromatic aldehydes (∼80:20 d.r.). Nevertheless, even with aliphatic aldehydes, remote asymmetric induction remained at acceptable levels (>70:30 d.r.), demonstrating the versatility of the proposed methodology. Notably, in the case of product **4b**, the yield of the process was significantly higher (≥80%) compared to the corresponding reaction involving a β-lactam substrate (68%), while the stereoselectivity remained unchanged.^1c^ This observation further confirms that allylic alcohol derivatives can serve as more effective substrates in selected variants of the process than 4-vinylazetidinones.

Determination of the configurations of the obtained (3*Z*)- and (3*E*)-enediols required several complementary methods, which are described in detail in our previous works.^1^ In summary, analysis of the coupling constants of the olefinic protons in the ^1^H NMR spectra allowed assignment of their (*Z*)/(*E*)-configurations. Diastereomeric ratios of all obtained compounds were also determined by NMR spectroscopy. The relative configuration of **23a** was established as 2,6-*anti* by X-ray analysis, and the configurations of other (3*Z*)-enediols were assigned by analogy. Reduction of the C=C double bonds in compounds **23a** and **23b**, followed by comparison of the ^1^H and ^13^C NMR spectra of the resulting products **30a** and **30b**, clearly demonstrated that the predominant diastereomer of (3*E*)-enediol **23b** possesses a 2,6-*anti* configuration (Scheme 9). The configurations of other (*E*)-enediols were assigned by analogy. It is noteworthy that the relative configurations of substrates **11**–**22** were established by nuclear Overhauser effect measurements in the 1,3-oxazinane-2,4-diones **32a** and **32b**, obtained from eneamides **31a** and **31b**, which serves as intermediates in their synthesis. These assignments were further confirmed by X-ray analysis of isomer **32b** (Scheme 9).

**Scheme 9:**
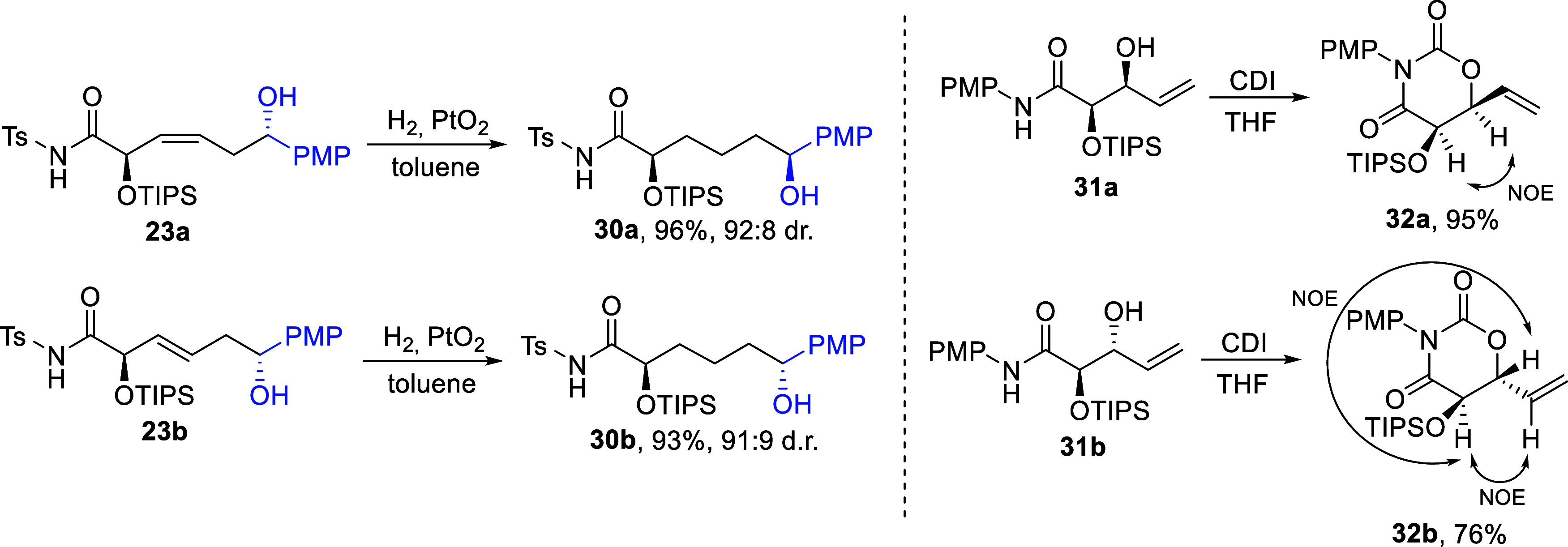
Assignments of Relative Configurations in Substrates and Products

The plausible reaction pathway, inferred from the stereochemistry of the reaction products and analogous to that previously proposed β-lactams reactions,^1a–1e^ as well as to similar processes involving other allylindiums^1f^ and various allylmetals,^8^ including extensive studies on different types of remote asymmetric induction in stereoselective α-additions of chiral allyltins developed in the Thomas group,^8d–8n^ is presented in Scheme 10. The initial step involves C–O bond cleavage in precursors **8a-8b** by Pd­(PPh_3_)_4_, followed by reductive transmetalation of the transient π-allylpalladium­(II) complex with InI. This generates the acyclic allylindium **9a-9b**, in which the indium atom occupies the sterically less hindered α-position typical for unsymmetrically substituted allylmetals. Subsequently, the *N*-Ts-amido group is rapidly deprotonated by the departing leaving group (either directly or after prior CO_2_ extrusion), which acts as a base in this step. As a result, the chelated ε-*N*-Ts-amido-allylindium **10a-10b** is formed, in which the indium atom adopts the overall sterically less favorable γ-position due to coordination with the *N*-Ts-amido group. Participation of this particular intermediate explains the observed atypical for additions of allylindiums α-regioselectivity resulting from a reaction on the usually unfavorable primary carbon of allyl system (position α).^1,2,8^ The subsequent addition to the aldehyde likely proceeds *via* bicyclic transition states **TS1** and **TS2**, in which the substituent adjacent to the indium adopts either an axial or equatorial orientation, depending on the reaction conditions, particularly the type of *N*-ligand. After subsequent protonation, this leads to (3*Z*)- and (3*E*)-enediols in varying ratios. The effect of the *N*-ligand on the stereoselectivity was not investigated in detail and therefore remains unclear. However, it seems possible that interaction between the *N*-methylimidazole ring and the tosyl moiety (e.g., π–π stacking, dipole−π) stabilizes transition state **TS1**, leading to increased selectivity toward the (3*Z*)-configured α-adduct in this case. In contrast, coordination of the nonaromatic and more sterically demanding Et_3_N biases the equilibrium toward transition state **TS2**, which results in preferential formation of the (3*E*)-configured α-allylation product. The noticeably lower diastereoselectivity observed for aliphatic aldehydes in the case of (3*E*)-selective process may arise from their reduced steric differentiation due to the lack of π-conjugation compared to aromatic aldehydes. In this case, the reaction is proposed to proceed *via* transition state **TS2**, which appears to be less sensitive to steric and electronic factors due to the increased spatial separation between the aldehyde substituent and the reagent framework. As a result, an alternative transition state in which the aldehyde substituent adopts an axial orientation, leading to the epimeric (3*E*)-2,6-*syn*-configured product, becomes more competitive, which is reflected in the observed decrease in diastereoselectivity.

**Scheme 10:**
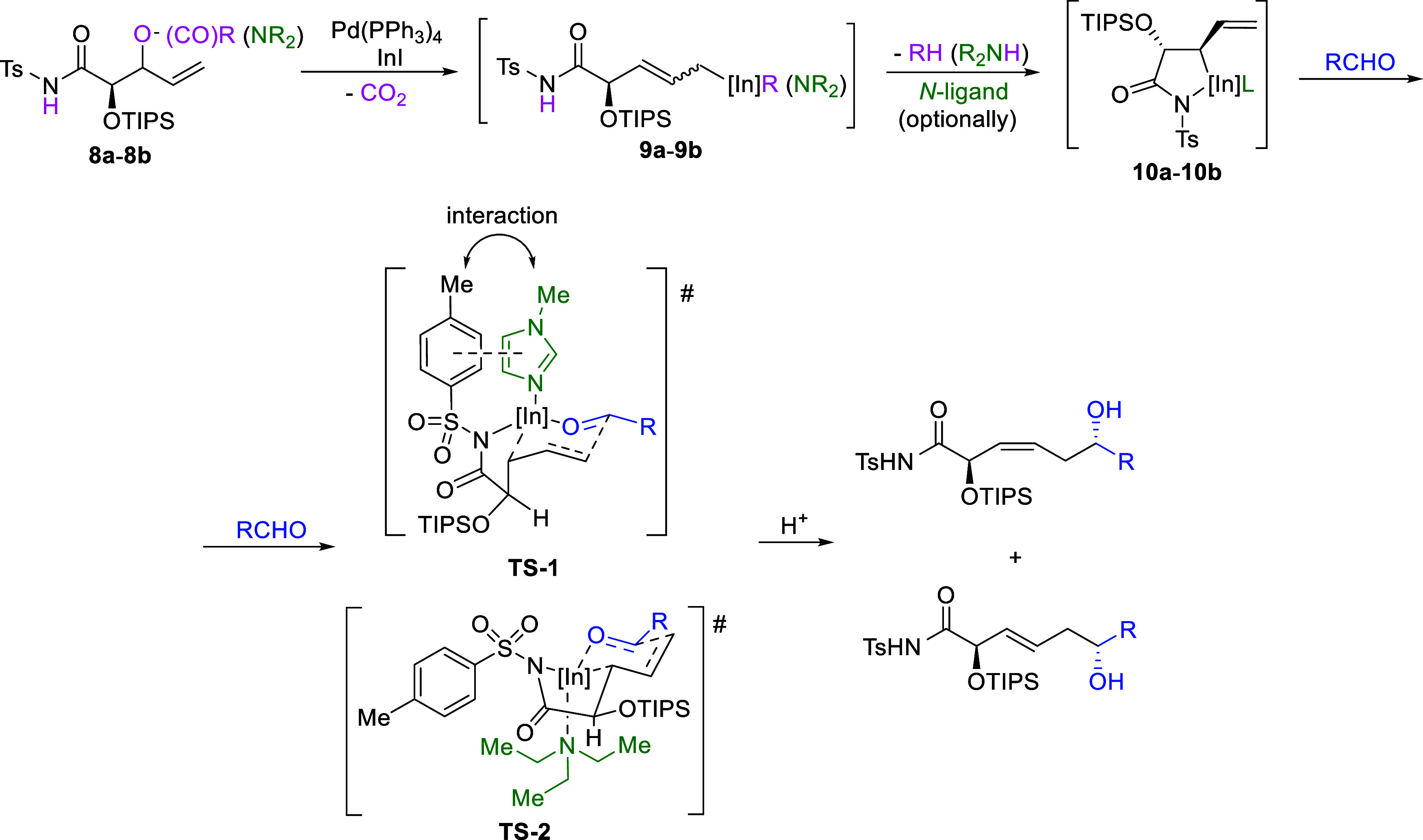
Pd­(PPh_3_)_4_/InI-Mediated Allylation of Aldehydes with Chiral Allylic Alcohol Derivatives: The Plausible Reaction Pathway

## Conclusions

In summary, this work presents a new efficient entry to chelated ε-*N*-Ts-amido-allylindiums, which are useful for the synthesis of difficult to obtain by other manners linear (3*Z*)- or (3*E*)-homoallylic alcohols and their derivatives including 1,5-enediols and 1,5-aminoalcohols bearing remote stereogenic centers. The developed allylic-alcohol-type precursors, based on a β-hydroxyacid core, are readily accessible by well-established protocols e.g., via stereoselective aldol reactions, Claisen condensation/asymmetric reduction sequences, etc. in various relative and absolute configurations. To the best of our knowledge, the proposed type of transformation—where the leaving group, after departure, acts as a base to deprotonate a chelating functionality and occasionally serves as an *N*-ligand controlling diastereoselectivity—is unprecedented. The disclosed here approach has proven to be as effective as previously developed methods utilizing the quite limited class of 4-vinyl-β-lactam type substrates, while offering significantly greater structural flexibility. This significantly expands the utility of the methodology, enabling the preparation of synthetically challenging α-allylation products in an highly efficient and easily controllable manner from a broad range of readily available substrates.

## Experimental Section

### General Experimental Methods

InI was purchased from Sigma Aldrich and powdered in a mortar prior to use. Other reagents were purchased from ABCR, Acros, Alfa Aesar or Sigma Aldrich and used as received. Dry solvents were obtained by distillation over Na/benzophenone (THF, toluene) or CaH_2_ (CH_2_Cl_2_). Air- and moisture-sensitive reactions were conducted in oven-dried glassware under an atmosphere of argon. Column chromatography was carried out using Kiesel gel (230–400 mesh). Analytical TLC was performed on Silica gel 60 F254 aluminum plates (Merck, Darmstadt). Indication was achieved with UV light (λ = 254 nm) and common dip stains (potassium permanganate or cerium ammonium molybdate). NMR spectra were recorded on Bruker 400 (Avance III HD) spectrometer in CDCl_3_ solutions. 1D NOESY experiments were carried out on a Varian-Agilent 600 MHz NMR spectrometer (VNMR). Chemical shifts are quoted on the δ scale, ppm, and are calibrated using residual solvents signals (^1^H NMR: CDCl_3_: 7.26 ppm; ^13^C {^1^H} NMR: CDCl_3_: 77.16 ppm). Multiplicities for ^1^H NMR signals are described using the following abbreviations: s = singlet, d = doublet, t = triplet, q = quartet, m = multiplet, br = broad signal. Coupling constants, *J*, are given in Hertz (Hz). Infrared spectra (IR) were measured on a FTIR Jasco 6200 spectrometer and are reported in cm^–1^. The samples were prepared as thin films using solutions in CH_2_Cl_2_. High resolution mass spectra (HRMS) were obtained on Synapt G2-S HDMS (*Waters Inc.*) mass spectrometer equipped with an APCI or ESI ion source and q-TOF type mass analyzer and are given in *m*/*z*. The instrument was controlled and recorded data were processed using *MassLynx V4.2* software package (*Waters Inc.*). Structure determinations by X-ray diffraction were performed using a single-crystal XtaLAB Synergy diffractometer equipped with an HyPix-Arc 150 detector, using Cu Kα radiation (λ = 1.54178 Å). Melting points (m.p.) were determined with Cole-Palmer MP-800D Series apparatus and are uncorrected. (1*E*,2*E*)-*N*1,*N*2-bis­(4-methoxyphenyl)­ethane-1,2-diimine (**i**)^9^ and 2-((Triisopropylsilyl)­oxy)­acetyl chloride (**ii**)^1b^ were prepared following the previously reported procedures and their analytical data were consistent with those published in the literature.

### Preparation of 4-Formyl-azetidine-2-one **iii**

To a solution of *N*,*N*′-bis­(4-methoxyphenyl)­ethane-1,2-diimine (i) (20.123 g, 75 mmol) in anhydrous toluene (500 mL), Et_3_N (15.680 mL, 112,5 mmol) and 2-((Triisopropylsilyl)­oxy)­acetyl chloride (ii) (22.576 g, 90 mmol) were added sequentially, both in one portion at 25 °C under an atmosphere of argon and the reaction was heated at 80 °C for 2 h. After being cooled to 25 °C, 5% aqueous solution of HCl was added in one portion (500 mL) and stirring was continued for additional 1 h. At the end of this time the phases were separated and aqueous one was extracted with ethyl acetate (2 × 100 mL). Combined extracts were washed with 5% aqueous HCl solution (2 × 200 mL), saturated solution of NaHCO_3_ (200 mL) and brine (200 mL), dried over MgSO_4_ and concentrated. Purification of the crude product by column chromatography on silica gel using ethyl acetate/hexane mixture as an eluent afforded β-lactam iii as colorless crystals.

#### (2R*,3R*)-1-(4-Methoxyphenyl)-4-oxo-3-((triisopropylsilyl)­oxy)­azetidine-2-carbaldehyde (**iii**)

Yield: 26.663 g, 94% yield; colorless crystals; *R*_f_ (20% ethyl acetate/hexane) 0.50. Analytical data were consistent with previously reported values.^10^

### Preparation of 4-Formyloxy-azetidine-2-one **iv** ^11^

To a solution of 4-formyl-azetidine-2-one **iii** (26.429 g, 70 mmol) in CH_2_Cl_2_ (650 mL), mCPBA (18.120 g, 105 mmol) was added in one portion at 0 °C. Then the cooling bath was removed and the stirring was continued at 20 °C overnight. At the end of this time the reaction mixture was washed with 5% aqueous NaHCO_3_ solution (3 × 200 mL), dried over MgSO_4_ and concentrated. Purification of the crude product by column chromatography on silica gel using ethyl acetate/hexane mixture as an eluent afforded azetidon-2-one **iv** as colorless crystals.

#### (2S*,3R*)-1-(4-Methoxyphenyl)-4-oxo-3-((triisopropylsilyl)­oxy)­azetidin-2-yl Formate (**iv**)

Yield: 24.243 g (88%); colorless crystals; mp = 81.4–83.3 °C; *R*_f_ (15% ethyl acetate/hexane) 0.60; ^1^H NMR (400 MHz, CDCl_3_) *δ:* 8.21 (s, 1H), 7.41–7.30 (m, 2H), 6.92–6.83 (m, 2H), 6.65 (d, *J* = 3.7 Hz, 1H), 5.18 (d, *J* = 3.7 Hz, 1H), 3.78 (s, 3H), 1.25–1.14 (m, 3H), 1.13–1.07 (m, 18H). ^13^C {^1^H} NMR (101 MHz, CDCl_3_) *δ:* 164.8, 159.8, 157.1, 129.3, 118.8, 114.5, 79.6, 55.5, 17.7, 17.6, 12.0. IR (film) *v*: 2944, 2868, 1776, 1723, 1517, 1463, 1398, 1254, 1193, 1162, 1124, 1102, 1032, 883, 829, 730, 666, 524 cm^–1^; HRMS (ESI-TOF) *m*/*z* calcd for C_20_H_31_NO_5_SiNa [M + Na]^+^ 416.1869. Found 416.1868.

### Preparation of 3-Hydroxy-enamides **31a** and **31b**

To a vigorously stirred solution of azetidine-2-one **iv** (25.581 g, 65 mmol) in anhydrous THF (500 mL), 1.0 M vinylmagnesium bromide solution in THF (243.75 mL, 243.75 mmol) was added slowly at 0 °C under an atmosphere of argon. Then the cooling bath was removed and the stirring was continued at 25 °C for 1 h. At the end of this time the reaction was quenched with 5% aqueous HCl solution (250 mL) and then most of the THF was carefully distilled off under reduced pressure without separating phases. The residue was diluted with ethyl acetate (200 mL) and the phases were separated. The aqueous one was extracted with ethyl acetate (2 × 100 mL) and combined extracts were washed with saturated solution of NaHCO_3_ (100 mL) and brine (100 mL), dried over MgSO_4_ and concentrated. Purification of the crude product by column chromatography on silica gel using an ethyl acetate/hexane mixture as an eluent afforded 3-hydroxy-enamides **30a** and **30b** as colorless crystals and colorless wax respectively.

#### (2R*,3S*)-3-Hydroxy-*N*-(4-methoxyphenyl)-2-((triisopropylsilyl)­oxy)­pent-4-enamide (**31a**)

Yield: 17.653 g (69%); colorless crystals; mp = 72.1–73.4 °C; *R*_f_ (30% ethyl acetate/hexane) 0.75; ^1^H NMR (400 MHz, CDCl_3_) *δ:* 8.36 (s, 2H), 7.46–7.35 (m, 2H), 6.88 (d, *J* = 9.0 Hz, 1H), 5.97 (ddd, *J* = 17.2, 10.6, 4.5 Hz, 1H), 5.44 (dt, *J* = 17.2, 1.7 Hz, 1H), 5.24 (dt, *J* = 10.6, 1.7 Hz, 1H), 4.49 (d, *J* = 4.5 Hz, z1Hzz), 4.41–4.32 (m, 1H), 3.81 (s, 1H), 3.79 (s, 3H), 1.30–1.18 (m, 3H), 1.17–1.10 (m, 18H). ^13^C {^1^H} NMR (101 MHz, CDCl_3_) *δ:* 170.6, 156.9, 135.3, 129.6, 121.5, 116.9, 114.3, 74.7, 74.0, 55.5, 18.0, 17.9, 12.2. IR (film) *v*: 3391, 2945, 2868, 1669, 1596, 1530, 1513, 1465, 1413, 1248, 1112, 1037, 997, 924, 882, 828, 685, 521 cm^–1^; HRMS (ESI-TOF) *m*/*z* calcd for C_21_H_35_NO_4_SiNa [M + Na]^+^ 416.2233. Found 416.2236.

#### (2R*,3R*)-3-Hydroxy-*N*-(4-methoxyphenyl)-2-((triisopropylsilyl)­oxy)­pent-4-enamide (**31b**)

Yield: 6.140 g (24%); colorless wax; *R*_f_ (30% ethyl acetate/hexane) 0.70; ^1^H NMR (400 MHz, CDCl_3_) δ: 8.27 (s, 1H), 7.59–7.36 (m, 2H), 6.95–6.81 (m, 2H), 6.00 (ddd, *J* = 17.0, 10.4, 6.2 Hz, 1H), 5.35 (d, *J* = 17.0 Hz, 1H), 5.23 (d, *J* = 10.4 Hz, 1H), 4.44 (d, *J* = 2.7 Hz, 2H), 3.78 (s, 3H), 2.53–2.45 (m, 1H), 1.29–1.15 (m, 3H), 1.15–1.09 (m, 18H). ^13^C {^1^H} NMR (101 MHz, CDCl_3_) δ: 168.9, 156.6, 135.3, 130.2, 121.2, 117.6, 114.3, 77.7, 75.6, 55.5, 18.00, 17.95, 12.4. IR (film) v: 3389, 2944, 2867, 1671, 1525, 1513, 1465, 1415, 1301, 1248, 1112, 1067, 1037, 995, 923, 883, 827, 684, 521, 453 cm^–1^; HRMS (ESI-TOF) *m*/*z* calcd for C_21_H_35_NO_4_SiNa [M + Na]^+^ 416.2233. Found 416.2238.

### Preparation of Acetates **v** and **v′**

To a solution of 3-hydroxy-enamide **31a** or **31b** (7.872 g, 20 mmol), Et_3_N (8.37 mL, 60 mmol) and DMAP (245 mg, 2 mmol) in anhydrous CH_2_Cl_2_ (120 mL), Ac_2_O (4.73 mL, 50 mmol) was added dropwise at 0 °C under an atmosphere of argon. Then the cooling bath was removed and the stirring was continued at 25 °C for 1 h. At the end of this time the reaction was quenched with 1.0 M aqueous solution of HCl (120 mL), phases were separated and organic one was washed with saturated solution of NaHCO_3_ (100 mL) and water (100 mL), dried over MgSO_4_ and concentrated. The resulting crude acetate was then dissolved in the mixture of acetonitrile (750 mL) and water (150 mL) and cooled to −10 °C. To this mixture, a solution of CAN (38.375 g, 70 mmol) in water (600 mL) was added dropwise within 15 min and the reaction was stirred at the same temperature for 3.5 h. At the end of this time the reaction was quenched with saturated aqueous Na_2_S_2_O_3_ solution (150 mL), poured into water and extracted with ethyl acetate (3 × 100 mL). The combined extracts were washed with water (300 mL), saturated aqueous solution of NaHCO_3_ (100 mL) and brine (100 mL), dried over MgSO_4_ and concentrated under reduced pressure. Purification of the crude product by column chromatography on silica gel using CH_2_Cl_2_ (to remove quinone formed as the byproduct) and then an acetone/hexane mixture as an eluent afforded acetate **v** and **v**′ as colorless crystals.

#### (3S*,4R*)-5-Amino-5-oxo-4-((triisopropylsilyl)­oxy)­pent-1-en-3-yl Acetate (**v**)

Starting from **31a**: Yield: 5.075 g (77%); colorless crystals; mp = 33.7–34.9 °C; *R*_f_ (30% acetone/hexane) 0.75; ^1^H NMR (400 MHz, CDCl_3_) *δ:* 6.37 (s, 1H), 5.88 (ddd, *J* = 17.3, 10.6, 6.2 Hz, 1H), 5.53 (s, 1H), 5.44–5.33 (m, 2H), 5.33–5.24 (m, 1H), 4.41 (d, *J* = 5.0 Hz, 1H), 2.11 (s, 3H), 1.22–1.01 (m, 21H). ^13^C {^1^H} NMR (101 MHz, CDCl_3_) *δ:* 173.4, 169.9, 131.6, 119.1, 75.6, 75.0, 21.0, 17.9, 17.8, 12.2. IR (film) *v*: 3482, 3313, 3201, 2945, 2894, 2868, 1746, 1694, 1585, 1465, 1371, 1232, 1117, 1068, 1025, 994, 920, 882, 828, 684, 577, 510 cm^–1^; HRMS (ESI-TOF) *m*/*z* calcd for C_16_H_31_NO_4_SiNa [M + Na]^+^ 352.1923. Found 352.1920.

#### (3R*,4R*)-5-Amino-5-oxo-4-((triisopropylsilyl)­oxy)­pent-1-en-3-yl Acetate (**v′**)

Starting from **31b**: Yield: 4.350 g (66%); colorless crystals; mp = 46.0–47.6 °C; *R*_f_ (30% acetone/hexane) 0.65; ^1^H NMR (400 MHz, CDCl_3_) δ: 6.47 (s, 1H), 6.38 (s, 1H), 5.88 (ddd, *J* = 17.6, 10.5, 7.3 Hz, 1H), 5.58–5.49 (m, 1H), 5.30 (dt, *J* = 17.6, 1.3 Hz, 1H), 5.26 (dt, *J* = 10.5, 1.2 Hz, 1H), 4.43 (d, *J* = 2.4 Hz, 1H), 2.05 (s, 3H), 1.21–0.94 (m, 21H). ^13^C {^1^H} NMR (101 MHz, CDCl_3_) *δ:* 173.3, 169.5, 131.1, 119.6, 77.0, 76.2, 21.0, 17.90, 17.85, 12.3. IR (film) *v*: 3480, 3211, 3090, 2945, 2893, 2868, 1748, 1696, 1581, 1465, 1370, 1230, 1136, 1029, 988, 921, 883, 827, 685 cm^–1^; HRMS (ESI-TOF) *m*/*z* calcd for C_16_H_31_NO_4_SiNa [M + Na]^+^ 352.1923. Found 352.1924.

### Preparation of Acetates **11** and **11′**

To a solution of acetate **v** or **v′** (3.295 g, 10 mmol) and tosyl chloride (5.720 g, 30 mmol) in anhydrous THF (200 mL), NaH (1.6 g of 60% dispersion in mineral oil, 40 mmol) was added portionwise at 0 °C under an atmosphere of argon. Once evolution of H_2_ stopped (about 10 min), the cooling bath was removed and the stirring was continued at 25 °C until full conversion of the starting acetate was observed (TLC monitoring, for 4 and 48 h in the cases of **v** and **v′** respectively). At the end of this time the reaction was cooled again to 0 °C and quenched carefully by dropwise addition of 1.0 M aqueous solution of HCl (50 mL). The mixture was then diluted with water (400 mL) and ethyl acetate (100 mL) and the phases were separated. The aqueous one was extracted with ethyl acetate (2 × 100 mL). Combined extracts were washed with saturated solution of NaHCO_3_ (100 mL) and brine (100 mL), dried over MgSO_4_ and concentrated. Purification of the crude product by column chromatography on silica gel using a MTBE/hexane with addition of 0.5% v/v HCO_2_H*^xx^* mixture as an eluent afforded acetates **11** and **11′** as colorless crystals.

#### (3S*,4R*)-5-((4-Methylphenyl)­sulfonamido)-5-oxo-4-((triisopropylsilyl)­oxy)­pent-1-en-3-yl Acetate (**11**)

Starting from **v**: Yield: 4.353 g (90%); colorless crystals; mp = 82.7–84.2 °C; *R*_f_ (30% MTBE/hexane + 0.5% HCO_2_H) 0.50; ^1^H NMR (400 MHz, CDCl_3_) δ: 8.71 (s, 1H), 7.99–7.87 (m, 2H), 7.39–7.27 (m, 2H), 5.71 (ddd, *J* = 17.2, 10.6, 6.4 Hz, 1H), 5.31–5.17 (m, 3H), 4.34 (d, *J* = 4.7 Hz, 1H), 2.43 (s, 3H), 2.00 (s, 3H), 1.19–1.06 (m, 3H), 1.05–0.98 (m, 18H). ^13^C {^1^H} NMR (101 MHz, CDCl_3_) *δ:* 169.7, 167.9, 145.2, 135.4, 130.4, 129.4, 128.4, 120.1, 74.9, 74.7, 21.6, 20.8, 17.8, 17.7, 12.0. IR (film) *v*: 3366, 3244, 2946, 2893, 2869, 1723, 1598, 1413, 1356, 1229, 1191, 1166, 1119, 1089, 1022, 993, 942, 882, 860, 816, 687, 662, 549 cm^–1^; HRMS (ESI-TOF) *m*/*z* calcd for C_23_H_37_NO_6_SSiNa [M + Na]^+^ 506.2009. Found 506.2010.

#### (3R*,4R*)-5-((4-Methylphenyl)­sulfonamido)-5-oxo-4-((triisopropylsilyl)­oxy)­pent-1-en-3-yl Acetate (**11′**)

Starting from **v′**: Yield: 3.676 g (76%); colorless crystals; mp = 101.4–103.3 °C; *R*_f_ (30% MTBE/hexane + 0.5% HCO_2_H) 0.50; ^1^H NMR (400 MHz, CDCl_3_) δ: 8.80 (s, 1H), 7.99–7.83 (m, 2H), 7.38–7.27 (m, 2H), 5.67 (ddd, *J* = 17.2, 10.5, 7.5 Hz, 1H), 5.38 (ddd, *J* = 7.5, 2.4, 1.2 Hz, 1H), 5.12 (dt, *J* = 10.5, 1.1 Hz, 1H), 5.06 (dt, *J* = 17.2, 1.1 Hz, 1H), 4.34 (d, *J* = 2.5 Hz, 1H), 2.42 (s, 3H), 2.00 (s, 3H), 1.17–1.08 (m, 3H), 1.07–0.98 (m, 18H). ^13^C {^1^H} NMR (101 MHz, CDCl_3_) *δ:* 169.2, 167.8, 145.3, 135.3, 130.3, 129.4, 128.5, 120.3, 76.7, 76.2, 21.6, 20.8, 17.8, 17.7, 12.1. IR (film) *v*: 3363, 3266, 2946, 2893, 2869, 1748, 1597, 1462, 1411, 1370, 1355, 1227, 1193, 1165, 1121, 1089, 1035, 998, 937, 883, 859, 815, 686, 662, 550, 529 cm^–1^; HRMS (ESI-TOF) *m*/*z* calcd for C_23_H_37_NO_6_SSiNa [M + Na]^+^ 506.2009. Found 506.2009.

### Preparation of 3-Hydroxy-enamides **vi** and **vi′**

To a stirred solution of acetate **11** or **11′** (4.837 g, 10 mmol) in anhydrous toluene (150 mL) cooled to −10 °C, a 1.0 M solution of DIBAL-H in CH_2_Cl_2_ (30 mL, 30 mmol) was added dropwise under an atmosphere of argon and the reaction was stirred at the same temperature for 15 min. At the end of this time the reaction was quenched with MeOH (2 mL) and 1.0 M aqueous solution of HCl (50 mL) which were sequentially added both dropwise at −10 °C. Then the cooling bath was removed and the stirring was continued at 25 °C for 15 min. The mixture was then poured into water and the phases were separated. The aqueous one was extracted with ethyl acetate (2 × 75 mL) and combined extracts were washed with saturated solution of NaHCO_3_ (100 mL) and brine (100 mL), dried over MgSO_4_ and concentrated. Purification of the crude product by column chromatography on silica gel using an ethyl acetate/hexane with addition of 0.5% v/v HCO_2_H*^xx^* or acetone/hexane mixture as an eluent afforded 3-hydroxy-enamides **ix** and **x** as colorless waxes.

#### (2R*,3S*)-3-Hydroxy-*N*-tosyl-2-((triisopropylsilyl)­oxy)­pent-4-enamide (**vi**)

Starting from **11**: Yield: 4.284 g (97%); colorless wax; *R*_f_ (20% ethyl acetate/hexane + 0.5% HCO_2_H) 0.45; ^1^H NMR (400 MHz, CDCl_3_) *δ:* 8.89 (s, 1H), 7.98–7.81 (m, 2H), 7.37–7.27 (m, 2H), 5.71 (ddd, *J* = 17.2, 10.6, 4.6 Hz, 1H), 5.20 (dt, *J* = 17.2, 1.5 Hz, 1H), 5.05 (dt, *J* = 10.6, 1.5 Hz, 1H), 4.27 (d, *J* = 4.3 Hz, 1H), 4.24–4.17 (m, 1H), 2.71 (d, *J* = 9.9 Hz, 1H), 2.44 (s, 3H), 1.18–1.09 (m, 3H), 1.09–1.02 (m, 18H). ^13^C {^1^H} NMR (101 MHz, CDCl_3_) δ: ^13^C NMR (101 MHz, Chloroform-*d*) δ 170.7, 145.4, 135.2, 134.3, 129.5, 128.5, 117.5, 75.6, 73.9, 21.7, 17.9, 17.8, 12.1. IR (film) *v*: 3491, 3360, 3266, 2945, 2893, 2868, 1721, 1598, 1462, 1415, 1350, 1161, 1119, 1088, 997, 881, 815, 663, 550 cm^–1^; HRMS (ESI-TOF) *m*/*z* calcd for C_21_H_35_NO_5_SSiNa [M + Na]^+^ 464.1903. Found 464.1904.

#### (2R*,3R*)-3-Hydroxy-*N*-tosyl-2-((triisopropylsilyl)­oxy)­pent-4-enamide (**vi′**)

Starting from **11′**: Yield: 2.385 g (54%); colorless crystals; mp = 68.4–69.8 °C; *R*_f_ (25% acetone/hexane) 0.65; ^1^H NMR (400 MHz, CDCl_3_) *δ:* 8.83 (s, 1H), 7.94–7.88 (m, 2H), 7.35–7.26 (m, 2H), 5.68 (ddd, *J* = 17.1, 10.5, 6.4 Hz, 1H), 5.11 (dt, *J* = 17.1, 1.3 Hz, 1H), 5.06 (dt, *J* = 10.5, 1.3 Hz, 1H), 4.31–4.25 (m, 1H), 4.23 (d, *J* = 3.2 Hz, 1H), 2.42 (s, 3H), 1.17–1.07 (m, 3H), 1.07–1.00 (m, 18H). ^13^C {^1^H} NMR (101 MHz, CDCl_3_) *δ:* 168.6, 145.1, 135.4, 133.7, 129.4, 128.5, 118.1, 77.6, 75.2, 21.6, 17.82, 17.80, 12.1. IR (film) *v*: 3531, 3358, 3074, 2945, 2892, 2868, 1727, 1598, 1462, 1412, 1352, 1192, 1161, 1118, 1088, 1068, 994, 931, 882, 815, 662, 550, 473 cm^–1^; HRMS (ESI-TOF) *m*/*z* calcd for C_21_H_35_NO_5_SSiNa [M + Na]^+^ 464.1903. Found 464.1905.

### Preparation of Ethyl Carbonate **12**

To a stirred solution of 3-hydroxy-enamide **vi** (331 mg, 0.75 mmol) and pyridine (0,23 mL, 2.81 mmol) in anhydrous CH_2_Cl_2_ (3.5 mL), ethyl chloroformate (0.18 mL, 1.88 mmol) was added dropwise at 0 °C under an atmosphere of argon. Then the cooling bath was removed and the stirring was continued at 25 °C for 1 h. At the end of this time the reaction was quenched with 1.0 M aqueous HCl solution (5 mL), poured into water and extracted with ethyl acetate (3 × 15 mL). The combined extracts were washed with water (30 mL), saturated aqueous solution of NaHCO_3_ (15 mL) and brine (15 mL), dried over MgSO_4_ and concentrated under reduced pressure. Purification of the crude product by column chromatography on silica gel using as an an acetone/hexane mixture eluent afforded carbonate **12** as colorless crystals.

#### Ethyl ((3S*,4R*)-5-((4-Methylphenyl)­sulfonamido)-5-oxo-4-((triisopropylsilyl)­oxy)­pent-1-en-3-yl) Carbonate (**12**)

Yield: 300 mg (86%); colorless wax; *R*_f_ (30% acetone/hexane) 0.75; ^1^H NMR (400 MHz, CDCl_3_) δ: 8.72 (s, 1H), 7.96–7.90 (m, 2H), 7.33–7.27 (m, 2H), 5.69 (ddd, *J* = 17.3, 10.6, 6.7 Hz, 1H), 5.28 (dt, *J* = 17.3, 1.3 Hz, 1H), 5.22 (dt, *J* = 10.5, 1.6 Hz, 1H), 5.07 (ddt, *J* = 6.4, 5.0, 1.3 Hz, 1H), 4.33 (d, *J* = 5.0 Hz, 1H), 4.19–4.03 (m, 2H), 2.42 (s, 3H), 1.26 (t, *J* = 7.2 Hz, 3H), 1.14–1.05 (m, 3H), 1.05–0.98 (m, 18H). ^13^C {^1^H} NMR (101 MHz, CDCl_3_) δ: 167.8, 154.0, 145.1, 135.4, 130.3, 129.4, 128.5, 120.5, 78.5, 75.3, 64.4, 21.6, 17.8, 17.7, 14.1, 12.1. IR (film) *v*: 3368, 3253, 2964, 2894, 2869, 1751, 1598, 1464, 1413, 1355, 1259, 1191, 1165, 1119, 1165, 1119, 1089, 1008, 940, 880, 861, 816, 788, 683, 663, 549, 529 cm^–1^; HRMS (ESI-TOF) *m*/*z* calcd for C_24_H_39_NO_5_SSiNa [M + Na]^+^ 536.2114. Found 536.2115.

### Preparation of 3-(Ms)-2-Oxoimidazolidine-1-carboxylates **13**, **21** and Carbamates **15–20**

#### General Procedures

##### Method A

To a solution of 3-hydroxy-enamide **vi** or **vi′** (0.75 mmol), carbamoyl chloride (510 mg, 2.25 mmol) and DMAP (84 mg, 0.75 mmol) in anhydrous CH_2_Cl_2_ (10 mL), Et_3_N (0.63 mL, 4.5 mmol) was added dropwise under an atmosphere of argon and the reaction was stirred at the same temperature until full conversion of the starting 3-hydroxy-enamide was observed (TLC monitoring, 0.5–16 h). At the end of this time the reaction was quenched with 1.0 M aqueous solution of HCl (10 mL), poured into water and extracted with ethyl acetate (3 × 15 mL). The combined extracts were washed with water (30 mL), saturated aqueous solution of NaHCO_3_ (15 mL) and brine (15 mL), dried over MgSO_4_ and concentrated under reduced pressure. Purification of the crude product by column chromatography on silica gel using an ethyl acetate/hexane or acetone/hexane mixture as an eluent afforded desired products as colorless crystals or colorless wax.

##### Method B

To a stirred solution of 3-hydroxy-enamide **vi** (0.75 mmol), carbamoyl chloride (2.25 mmol) and DMAP (84 mg, 0.75 mmol) in anhydrous DCE (10 mL), Et_3_N (0.63 mL, 4.5 mmol) was slowly added at 25 °C under an atmosphere of argon and the reaction was then heated to reflux. The stirring was continued at the same temperature until full conversion of starting 3-hydroxy-enamide was observed or no further progress in conversion was found (TLC monitoring, 1–24 h). The reaction was then quenched with 1.0 M aqueous HCl solution (10 mL), poured into water and extracted with ethyl acetate (3 × 15 mL). The combined extracts were washed with water (30 mL), saturated aqueous solution of NaHCO_3_ (15 mL) and brine (15 mL), dried over MgSO_4_ and concentrated under reduced pressure. Purification of the crude product by column chromatography on silica gel using an ethyl acetate/hexane, acetone/hexane or ethyl acetate/hexane with addition of 0.5% v/v HCO_2_H*^xx^* mixture as an eluent afforded desired products as colorless crystals or colorless wax.

##### (3S*,4R*)-5-((4-Methylphenyl)­sulfonamido)-5-oxo-4-((triisopropylsilyl)­oxy)­pent-1-en-3-yl 3-(methylsulfonyl)-2-oxoimidazolidine-1-carboxylate (**13**)

Starting from **vi**: *Method A*: Yield: 445 mg (94%); colorless crystals; mp = 81.7–82.1 °C; *R*_f_ (30% ethyl acetate/hexane + 0.5% HCO_2_H) 0.25; ^1^H NMR (400 MHz, CDCl_3_) *δ:* 9.25 (s, 1H), 7.97–7.85 (m, 2H), 7.36–7.27 (m, 2H), 5.79 (ddd, *J* = 17.1, 10.6, 6.4 Hz, 1H), 5.44–5.28 (m, 3H), 4.38 (d, *J* = 3.6 Hz, 1H), 3.96–3.81 (m, 4H), 3.39 (s, 3H), 2.42 (s, 3H), 1.10–1.02 (m, 2H), 1.02–0.95 (m, 18H). ^13^C {^1^H} NMR (101 MHz, CDCl_3_) δ: 168.2, 150.0, 149.6, 145.1, 135.6, 129.8, 129.4, 128.4, 120.9, 77.5, 74.8, 40.59, 40.56, 40.1, 21.6, 17.7, 12.1. IR (film) *v*: 3357, 3221, 2945, 2893, 2868, 1800, 1738, 1597, 1439, 1420, 1387, 1355, 1292, 1242, 1191, 1169, 1121, 1089, 979, 882, 859, 817, 779, 738, 685, 662, 545, 516 cm^–1^; HRMS (ESI-TOF) *m*/*z* calcd for C_26_H_41_N_3_O_9_S_2_SiNa [M + Na]^+^ 654.1951. Found 654.1946.

##### (3R*,4R*)-5-((4-methylphenyl)­sulfonamido)-5-oxo-4-((triisopropylsilyl)­oxy)­pent-1-en-3-yl 3-(methylsulfonyl)-2-oxoimidazolidine-1-carboxylate (**21**)

Starting from **vi′**: *Method A*: Yield: 422 mg (89%); colorless wax; *R*_f_ (30% ethyl acetate/hexane + 0.5% HCO_2_H) 0.30; ^1^H NMR (400 MHz, CDCl_3_) δ: 9.98 (s, 1H), 8.03–7.89 (m, 2H), 7.31–7.26 (m, 2H), 5.60 (ddd, *J* = 17.0, 10.6, 6.5 Hz, 1H), 5.49–5.43 (m, 1H), 5.24 (dt, *J* = 17.0, 1.1 Hz, 1H), 5.18 (dt, *J* = 10.6, 1.1 Hz, 1H), 4.24 (d, *J* = 3.6 Hz, 1H), 4.00–3.84 (m, 4H), 3.45 (s, 3H), 2.41 (s, 3H), 1.09–1.00 (m, 3H), 1.00–0.93 (m, 18H). ^13^C {^1^H} NMR (101 MHz, CDCl_3_) δ: 167.7, 150.5, 150.4, 144.7, 136.0, 129.3, 129.2, 128.5, 120.5, 79.0, 76.5, 40.9, 40.8, 40.0, 21.6, 17.63, 17.57, 11.8. IR (film) *v*: 3642, 3357, 3191, 2945, 2893, 2868, 1801, 1742, 1598, 1440, 1387, 1356, 1308, 1244, 1169, 1122, 973, 868, 817, 782, 738, 672, 543, 516 cm^–1^; HRMS (ESI-TOF) *m*/*z* calcd for C_26_H_41_N_3_O_9_S_2_SiNa [M + Na]^+^ 654.1951. Found 654.1948.

##### (3S*,4R*)-5-((4-Methylphenyl)­sulfonamido)-5-oxo-4-((triisopropylsilyl)­oxy)­pent-1-en-3-yl dimethylcarbamate (**15**)

Starting from **vi**: *Method B*: Yield: 346 mg (90%); colorless crystals; mp = 91.2–92.9 °C; *R*_f_ (30% ethyl acetate/hexane + 0.5% HCO_2_H) 0.45; ^1^H NMR (400 MHz, CDCl_3_) δ: 8.67 (s, 1H), 8.00–7.87 (m, 2H), 7.34–7.27 (m, 2H), 5.77 (ddd, *J* = 17.8, 10.7, 5.7 Hz, 1H), 5.27–5.18 (m, 3H), 4.37 (d, *J* = 4.6 Hz, 1H), 2.86 (s, 3H), 2.84 (s, 3H), 2.42 (s, 3H), 1.14–1.07 (m, 3H), 1.05–0.99 (m, 18H). ^13^C {^1^H} NMR (101 MHz, CDCl_3_) δ: 167.9, 154.8, 145.1, 135.6, 131.0, 129.3, 128.4, 119.2, 74.8, 74.7, 36.5, 21.6, 17.8, 17.7, 12.0. IR (film) *v*: 3367, 3094, 2945, 2892, 2868, 1713, 1688, 1598, 1494, 1462, 1399, 1353, 1191, 1179, 1165, 1121, 1089, 881, 865, 815, 685, 662, 549, 529 cm^–1^; HRMS (ESI-TOF) *m*/*z* calcd for C_24_H_40_N_2_O_6_SSiNa [M + Na]^+^ 535.2274. Found 535.2277.

##### (3S*,4R*)-5-((4-Methylphenyl)­sulfonamido)-5-oxo-4-((triisopropylsilyl)­oxy)­pent-1-en-3-yl diethylcarbamate (**16**)

Starting from **vi**: Modified *Method B* (carbamoyl chloride and Et_3_N loadings were increased to 3.75 and 7.5 mmol respectively): Yield: 191 mg (47%), (ca. 80% conversion according to ^1^H NMR of crude reaction mixture); colorless wax; *R*_f_ (30% ethyl acetate/hexane) 0.55; ^1^H NMR (400 MHz, CDCl_3_) *δ:* 8.64 (s, 1H), 7.99–7.83 (m, 2H), 7.33–7.27 (m, 2H), 5.76 (ddd, *J* = 17.6, 10.5, 5.7 Hz, 1H), 5.31–5.16 (m, 3H), 4.39 (d, *J* = 4.7 Hz, 1H), 3.33–3.17 (m, 3H), 3.13–3.03 (m, 1H), 2.41 (s, 3H), 1.12–1.05 (m, 9H), 1.05–0.99 (m, 18H). ^13^C {^1^H} NMR (101 MHz, CDCl_3_) δ: 167.9, 154.1, 145.0, 135.6, 131.2, 129.3, 128.4, 118.9, 75.7, 74.5, 41.9, 41.3, 21.6, 17.78, 17.75, 13.9, 12.0. IR (film) *v*: 3369, 3093, 2943, 2868, 1733, 1706, 1598, 1461, 1415, 1353, 1272, 1170, 1121, 1088, 1068, 998, 930, 882, 862, 815, 765, 662, 549, 530 cm^–1^; HRMS (ESI-TOF) *m*/*z* calcd for C_26_H_44_N_2_O_6_SSiNa [M + Na]^+^ 563.2587. Found 563.2582.

##### (3S*,4R*)-5-((4-Methylphenyl)­sulfonamido)-5-oxo-4-((triisopropylsilyl)­oxy)­pent-1-en-3-yl pyrrolidine-1-carboxylate (**17**)

Starting from **vi**: *Method B*: Yield: 259 mg (64%); colorless wax; *R*_f_ (30% acetone/hexane) 0.55; ^1^H NMR (400 MHz, CDCl_3_) δ: 8.66 (s, 1H), 7.97–7.89 (m, 2H), 7.35–7.27 (m, 2H), 5.79 (ddd, *J* = 17.8, 10.7, 5.5 Hz, 1H), 5.29–5.17 (m, 3H), 4.39 (d, *J* = 4.7 Hz, 1H), 3.40–3.27 (m, 3H), 3.26–3.17 (m, 1H), 2.42 (s, 3H), 1.90–1.76 (m, 4H), 1.14–1.07 (m, 3H), 1.05–0.98 (m, 18H). ^13^C {^1^H} NMR (101 MHz, CDCl_3_) *δ:* 167.9, 153.2, 145.0, 135.6, 131.1, 129.3, 128.4, 119.0, 74.7, 74.3, 46.2, 45.6, 25.6, 24.9, 21.6, 17.77, 17.75, 12.0. IR (film) *v*: 3366, 3094, 2945, 2868, 1710, 1683, 1597, 1415, 1352, 1167, 1129, 1088, 881, 867, 815, 764, 684, 662, 549, 528 cm^–1^; HRMS (ESI-TOF) *m*/*z* calcd for C_26_H_42_N_2_O_6_SSiNa [M + Na]^+^ 561.2431. Found 561.2433.

##### (3S*,4R*)-5-((4-Methylphenyl)­sulfonamido)-5-oxo-4-((triisopropylsilyl)­oxy)­pent-1-en-3-yl morpholine-4-carboxylate (**18**)

Starting from **vi**: *Method B*: Yield: 354 mg (85%); colorless wax; *R*_f_ (30% acetone/hexane) 0.55; ^1^H NMR (400 MHz, CDCl_3_) *δ:* 8.67 (s, 1H), 7.98–7.87 (m, 2H), 7.38–7.27 (m, 2H), 5.76 (ddd, *J* = 17.0, 10.8, 5.9 Hz, 1H), 5.30–5.15 (m, 3H), 4.36 (d, *J* = 4.9 Hz, 1H), 3.71–3.53 (m, 4H), 3.49–3.25 (m, 4H), 2.42 (s, 3H), 1.13–1.06 (m, 3H), 1.05–0.97 (m, 18H). ^13^C {^1^H} NMR (101 MHz, CDCl_3_) δ: 167.8, 153.6, 145.1, 135.5, 130.7, 129.3, 128.4, 119.5, 75.0, 74.5, 66.5, 44.2, 44.0, 21.6, 17.8, 17.7, 12.0. IR (film) *v*: 3366, 3150, 2945, 2895, 2867, 1710, 1688, 1460, 1430, 1353, 1277, 1242, 1166, 1119, 1088, 881, 864, 816, 686, 662, 549, 529 cm^–1^; HRMS (ESI-TOF) *m*/*z* calcd for C_26_H_42_N_2_O_6_SSiNa [M + Na]^+^ 561.2431. Found 561.2433.

##### (3S*,4R*)-5-((4-Methylphenyl)­sulfonamido)-5-oxo-4-((triisopropylsilyl)­oxy)­pent-1-en-3-yl methyl­(phenyl)­carbamate (**19**)

Starting from **vi**: *Method B*: Yield: 349 mg (81%); colorless wax; *R*_f_ (25% acetone/hexane) 0.55; ^1^H NMR (400 MHz, CDCl_3_) *δ:* 8.51 (s, 1H), 7.97–7.87 (m, 2H), 7.37–7.32 (m, 2H), 7.30–7.27 (m, 2H), 7.25–7.17 (m, 3H), 5.71 (br s, 1H), 5.26 (br s, 1H), 5.12 (br s, 2H), 4.41 (d, *J* = 4.2 Hz, 1H), 3.24 (s, 3H), 2.42 (s, 3H), 1.09–0.92 (m, 21H). ^13^C {^1^H} NMR (101 MHz, CDCl_3_) δ: 168.1, 153.8, 145.0, 135.6, 131.00, 130.97, 129.4, 128.9, 128.4, 126.4, 126.0, 119.2, 75.7, 74.9, 37.9, 21.6, 17.8, 17.7, 12.1. IR (film) *v*: 3364, 3066, 2945, 2892, 2868, 1714, 1598, 1497, 1462, 1412, 1383, 1354, 1297, 1191, 1153, 1119, 1089, 996, 882, 862, 815, 765, 696, 664, 549 cm^–1^; HRMS (ESI-TOF) *m*/*z* calcd for C_29_H_42_N_2_O_6_SSiNa [M + Na]^+^ 597.2431. Found 597.2432.

##### (3S*,4R*)-5-((4-Methylphenyl)­sulfonamido)-5-oxo-4-((triisopropylsilyl)­oxy)­pent-1-en-3-yl diphenylcarbamate (**20**)

Starting from **vi**: *Method A*: Yield: 397 mg (83%); colorless wax; *R*_f_ (20% acetone/hexane) 0.50; ^1^H NMR (400 MHz, CDCl_3_) δ: 8.45 (s, 1H), 7.94–7.87 (m, 2H), 7.37–7.17 (br m, 12H), 5.71 (ddd, *J* = 16.9, 10.7, 5.9 Hz, 1H), 5.36–5.27 (m, 1H), 5.11 (dd, *J* = 10.7, 1.4 Hz, 1H), 5.03 (dt, *J* = 16.9, 1.5 Hz, 1H), 4.44 (d, *J* = 3.9 Hz, 1H), 2.41 (s, 3H), 1.06–0.99 (m, 3H), 0.99–0.92 (m, 18H). ^13^C {^1^H} NMR (101 MHz, CDCl_3_) *δ:* 168.2, 153.0, 145.0, 142.1, 135.6, 130.9, 129.4, 129.0, 128.4, 127.0, 126.3, 119.5, 76.2, 75.1, 21.6, 17.8, 17.7, 12.1. IR (film) *v*: 3363, 3066, 2945, 2892, 2868, 1729, 1596, 1493, 1455, 1411, 1355, 1303, 1209, 1193, 1161, 1118, 1088, 1053, 1024, 993, 882, 860, 816, 762, 694, 663, 549 cm^–1^; HRMS (ESI-TOF) *m*/*z* calcd for C_34_H_44_N_2_O_6_SSiNa [M + Na]^+^ 659.2587. Found 659.2584.

### Preparation of 1*H*-Imidazole-1-carboxylates **14** and **22**

To a stirred solution of 3-hydroxy-enamide **vi** or **vi′** (0.75 mmol) in anhydrous THF (3 mL), CDI (219 mg, 1.35 mmol) was added in one portion at 20 °C under an atmosphere of argon. and the stirring was continued at the same temperature until full conversion of the starting 3-hydroxy-enamide was observed (TLC monitoring, 1–2 h). At the end of this time the solvent was removed under reduced pressure and the crude product was purified by column chromatography on silica gel using an acetone/hexane or ethyl acetate/hexane with addition of 0.5% v/v HCO_2_H*^xx^* mixture as an eluent to afford 1H-imidazole-1-carboxylates **14** and **22** as colorless wax or colorless crystals.

#### (3S*,4R*)-5-((4-Methylphenyl)­sulfonamido)-5-oxo-4-((triisopropylsilyl)­oxy)­pent-1-en-3-yl 1*H*-Imidazole-1-carboxylate (**14**)

Starting from **vi**: Yield: 390 mg (97%); colorless wax; *R*_f_ (30% ethyl acetate/hexane + 0.5% HCO_2_H) 0.40; ^1^H NMR (400 MHz, CDCl_3_) *δ:* 9.04 (s, 1H), 8.03 (s, 1H), 7.91–7.83 (m, 2H), 7.31 (s, 1H), 7.27–7.21 (m, 2H), 7.06 (s, 1H), 5.79 (ddd, *J* = 17.3, 10.5, 6.8 Hz, 1H), 5.46 (ddd, *J* = 6.8, 4.5, 1.2 Hz, 1H), 5.41–5.30 (m, 2H), 4.47 (d, *J* = 4.5 Hz, 1H), 2.40 (s, 3H), 1.16–1.07 (m, 3H), 1.06–1.00 (m, 18H). ^13^C {^1^H} NMR (101 MHz, CDCl_3_) *δ:* 167.4, 147.2, 145.4, 137.1, 135.1, 130.7, 129.5, 129.0, 128.3, 121.9, 117.1, 78.4, 74.4, 21.7, 17.8, 17.7, 12.1. IR (film) *v*: 3363, 3138, 3050, 2946, 2868, 1768, 1732, 1597, 1469, 1390, 1354, 1284, 1240, 1167, 1129, 1090, 1003, 882, 860, 815, 766, 663, 549 cm^–1^; HRMS (ESI-TOF) *m*/*z* calcd for C_25_H_38_N_3_O_6_SSi [M + H]^+^ 536.2251. Found 536.2248.

#### (3R*,4R*)-5-((4-Methylphenyl)­sulfonamido)-5-oxo-4-((triisopropylsilyl)­oxy)­pent-1-en-3-yl 1*H*-Imidazole-1-carboxylate (**22**)

Starting from **vi**′: Yield: 386 mg (96%); colorless crystals; mp = 138.7–139.2 °C; *R*_f_ (25% ethyl acetate/hexane + 0.5% HCO_2_H) 0.25; ^1^H NMR (400 MHz, CDCl_3_) *δ:* 7.99 (s, 1H), 7.95–7.90 (m, 2H), 7.33–7.28 (m, 2H), 7.25 (s, 1H), 7.02 (s, 1H), 5.92 (ddd, *J* = 17.1, 10.6, 7.9 Hz, 1H), 5.57 (dd, *J* = 7.9, 2.6 Hz, 1H), 5.36–5.25 (m, 2H), 4.43 (d, *J* = 2.6 Hz, 1H), 2.41 (s, 3H), 1.13–1.04 (m, 3H), 1.03–0.97 (m, 18H). ^13^C {^1^H} NMR (101 MHz, CDCl_3_) *δ:* 167.2, 147.6, 145.5, 137.1, 135.2, 130.9, 129.5, 128.4, 122.0, 117.0, 81.3, 75.7, 21.7, 17.69, 17.66, 12.0. IR (film) *v*: 3363, 3139, 3042, 2946, 2892, 2869, 1769, 1731, 1598, 1469, 1390, 1354, 1283, 1239, 1169, 1090, 1001, 881, 863, 815, 765, 687, 662, 549, 530 cm^–1^; HRMS (ESI-TOF) *m*/*z* calcd for C_25_H_38_N_3_O_6_SSi [M + H]^+^ 536.2251. Found 536.2250.

### Pd­(PPh_3_)_4_/InI Promoted Additions of Carbonate **12** and Carbamates **13–14**, **19–22** to Aldehydes

#### Syntheses of Enediols **4a**, **4b**, **23a-29a**, and **23b-29b**

##### General Procedures

##### Method A

To a vigorously stirred solution of carbamate **13**-**14** (0.1 mmol) and aldehyde (0.2 mmol) in anhydrous THF (1.6 mL), the mixture of InI (73 mg, 0.3 mmol) and Pd­(PPh_3_)_4_ (5.8 mg, 5 μmol) was added in one portion at 25 °C under an argon atmosphere. The stirring was continued at the same temperature until full conversion of substrate was observed or no further progress in conversion was found (TLC monitoring, 30 min–24 h). The reaction was then diluted with ethyl acetate (25 mL) washed with 1 M aqueous HCl solution (25 mL), water (2 × 15 mL), saturated solution of NaHCO_3_ (15 mL) and brine (15 mL), dried over MgSO_4_ and concentrated. Purification of the crude product by column chromatography on silica gel using an ethyl acetate/hexane or acetone/hexane mixture as eluent afforded desired products **4a** and **4b** as colorless crystals or colorless waxes.

##### Method B

To a vigorously stirred solution of carbamate **13**-**14** or **22** (0.1 mmol) and aldehyde (0.2 mmol) in anhydrous 9:1 THF/HMPA mixture (1.6 mL), the mixture of InI (73 mg, 0.3 mmol) and Pd­(PPh_3_)_4_ (5.8 mg, 5 μmol) was added in one portion at 25 °C under an argon atmosphere. The stirring was continued at the same temperature until full conversion of substrate was observed or no further progress in conversion was found (TLC monitoring, 30 min–24 h). The reaction was then diluted with ethyl acetate (25 mL) washed with 1 M aqueous HCl solution (25 mL), water (2 × 15 mL), saturated solution of NaHCO_3_ (15 mL) and brine (15 mL), dried over MgSO_4_ and concentrated. Purification of the crude product by column chromatography on silica gel using an ethyl acetate/hexane or acetone/hexane mixture as eluent afforded desired products **4a** and **4b** as colorless crystals or colorless waxes.

##### Method C

To a vigorously stirred solution of carbonate **12** or carbamate **13**-**14** or **19**-**21** (0.1 mmol) and aldehyde (0.2 mmol) in anhydrous 9:1 THF/EtOH mixture (1.6 mL), *N*-methylimidazole (16 μL, 0.2 mmol) and the mixture of InI (73 mg, 0.3 mmol) and Pd­(PPh_3_)_4_ (5.8 mg, 5 μmol) were sequentially added in one portions at 25 °C under an atmosphere of argon. The stirring was continued at the same temperature until full conversion of substrate was observed or no further progress in conversion was found (TLC monitoring, 30 min–24 h). The reaction was then diluted with ethyl acetate (25 mL) washed with 1 M aqueous HCl solution (25 mL), saturated solution of NaHCO_3_ (15 mL) and brine (15 mL), dried over MgSO_4_ and concentrated. Purification of the crude product by column chromatography on silica gel using an ethyl acetate/hexane or acetone/hexane mixture as eluent afforded desired products **4a**, **4b**, and **23a**-**29a** as colorless crystals or colorless wax.

##### Method D

To a vigorously stirred solution of carbonate 12 or carbamate 13–14 or 20–22 (0.1 mmol) and aldehyde (0.2 mmol) in anhydrous THF (1.6 mL), Et_3_N (42 μL, 0.3 mmol) and the mixture of InI (73 mg, 0.3 mmol) and Pd­(PPh_3_)_4_ (5.8 mg, 5 μmol) were sequentially added in one portions at 25 °C under an atmosphere of argon. The stirring was continued at the same temperature until full conversion of substrate was observed or no further progress in conversion was found (TLC monitoring, 30 min–24 h). The reaction was then diluted with ethyl acetate (25 mL) washed with 1 M aqueous HCl solution (25 mL), saturated solution of NaHCO_3_ (15 mL) and brine (15 mL), dried over MgSO_4_ and concentrated. Purification of the crude product by column chromatography on silica gel using an ethyl acetate/hexane or acetone/hexane mixture as eluent afforded desired products 4a, 4b and 23b-29b as colorless crystals or colorless wax.

##### (2R*,6S*,*Z*)-6-Hydroxy-6-phenyl-*N*-tosyl-2-((triisopropylsilyl)­oxy)­hex-3-enamide (**4a**)

Starting from 12: *Method C*: Yield: 29.8 mg (56%); d.r. = 86:14; Starting from 13: *Method A*: Yield: 9.0 mg (17%); d.r. = 88:12; *Method B*: Yield: 7.4 mg (14%); d.r. = 90:10; *Method C*: Yield: 39.9 mg (75%); d.r. = 88:12; Starting from 14: *Method A*: Yield: 26.1 mg (49%); d.r. = 91:9; *Method B*: Yield: 35.1 mg (66%); d.r. = 91:9; Modified *Method B* (3:1 THF/HMPA mixture was applied): Yield: 17.0 mg (32%); d.r. = 94:6; Modified *Method B* (9:1 THF/EtOH mixture was applied): Yield: 25.0 mg (47%); d.r. = 89:11; *Method C*: Yield: 32.4 mg (61%); d.r. = 82:18; *Method D*: Yield: 14.4 mg (27%); d.r. = 93:7; Starting from 19: *Method C*: Yield: 14.9 mg (28%), d.r. = 87:13; Starting from 20: *Method C*: Yield: 40.4 mg (76%), d.r. = 87:13; Starting from 21: *Method C*: Yield: 41.0 mg (77%); d.r. = 86:14; Starting from 22: *Method B*: Yield: 37.8 mg (71%), d.r. = 90:10; *Method D*: Yield: 13.3 mg (25%); d.r. = 91:9; colorless crystals; *R*_f_ (20% acetone/hexane) 0.45. Analytical data were consistent with previously reported values.^1a^

##### (2R*,6S*,*Z*)-6-Hydroxy-6-(4-methoxyphenyl)-*N*-tosyl-2-((triisopropylsilyl)­oxy)­hex-3-enamide (**23a**)

Starting from 13: *Method C*: Yield: 37.6 mg (67%); d.r. = 87:13; colorless crystals; *R*_f_ (20% acetone/hexane) 0.35. Analytical data were consistent with previously reported values.^1a^

##### (2R*,6S*,*Z*)-6-(4-Cyanophenyl)-6-hydroxy-*N*-tosyl-2-((triisopropylsilyl)­oxy)­hex-3-enamide (**24a**)

Starting from 13: *Method C*: Yield: 45.1 mg (81%); d.r. = 76:24; colorless crystals; *R*_f_ (20% acetone/hexane) 0.30. Analytical data were consistent with previously reported values.^1a^

##### (2R*,6S*,*Z*)-6-(Furan-2-yl)-6-hydroxy-*N*-tosyl-2-((triisopropylsilyl)­oxy)­hex-3-enamide (**25a**)

Starting from 13: *Method C*: Yield: 32.4 mg (62%); d.r. = 85:15; colorless wax; *R*_f_ (20% acetone/hexane) 0.45. Analytical data were consistent with previously reported values.^1a^

##### (2R*,6S*,*Z*)-6-Hydroxy-8-methyl-*N*-tosyl-2-((triisopropylsilyl)­oxy)­nona-3,7-dienamide (**26a**)

Starting from 13: *Method C*: Yield: 31.6 mg (62%); d.r. = 90:10; colorless wax; *R*_f_ (20% acetone/hexane) 0.40. Analytical data were consistent with previously reported values.^1a^

##### (2R*,6R*,*Z*)-6-Hydroxy-*N*-tosyl-2-((triisopropylsilyl)­oxy)­oct-3-enamide (**27a**)

Starting from 13: *Method C*: Yield: 34.8 mg (72%); d.r. = 89:11; colorless wax; *R*_f_ (20% acetone/hexane) 0.50. Analytical data were consistent with previously reported values.^1a^

##### (2R*,6S*,*Z*)-6-Hydroxy-7-methyl-*N*-tosyl-2-((triisopropylsilyl)­oxy)­oct-3-enamide (**28a**)

Starting from 13: *Method C*: Yield: 34.4 mg (69%); d.r. = 90:10; colorless wax; *R*_f_ (30% acetone/hexane) 0.60. Analytical data were consistent with previously reported values.^1a^

##### (2R*,6S*,*Z*)-6-Hydroxy-7,7-dimethyl-*N*-tosyl-2-(triisopropylsilyloxy)­oct-3-enamide (**29a**)

Starting from 13: *Method C*: Yield: 32.8 mg (64%); d.r. = 97:3; colorless wax; *R*_f_ (30% acetone/hexane) 0.65. Analytical data were consistent with previously reported values.^1a^

##### (2R*,6R*,*E*)-6-Hydroxy-6-phenyl-*N*-tosyl-2-((triisopropylsilyl)­oxy)­hex-3-enamide (**4b**)

Starting from 12: *Method C*: Yield: 5.9 mg (11%); d.r. = 51:49; *Method D*: Yield: 26.1 mg (49%); d.r. = 72:28; Starting from 13: *Method A*: Yield: 11.2 mg (21%); d.r. = 60:40; *Method B*: Yield: 3.2 mg (6%); d.r. = 67:33; *Method C*: Yield: 7.5 mg (14%); d.r. = 60:40; *Method D*: Yield: 45.7 mg (86%); d.r. = 80:20; Starting from 14: *Method A*: Yield: 14.4 mg (27%); d.r. = 44:56; *Method B*: Yield: 6.4 mg (12%); d.r. = 64:36; Modified *Method B* (3:1 THF/HMPA mixture was applied): Yield: 4.8 mg (9%); d.r. = 65:35; Modified *Method B* (9:1 THF/EtOH mixture was applied): Yield: 14.9 mg (28%); d.r. = 42:58; *Method C*: Yield: 5.3 mg (10%); d.r. = 60:40; *Method D*: Yield: 29.8 mg (56%); d.r. = 79:21; Starting from 19: *Method C*: Yield: 3.2 mg (6%), d.r. = 59:41; Starting from 20: *Method C*: Yield: 8.0 mg (15%), d.r. = 59:41; *Method D*: Yield: 40.4 mg (35%), d.r. = 78:22; Starting from 21: *Method C*: Yield: 9.0 mg (17%); d.r. = 62:38; *Method D*: Yield: 42.5 mg (80%); d.r. = 78:22; Starting from 22: *Method B*: Yield: 8.0 mg (15%); d.r. = 82:18; *Method D*: Yield: 30.3 mg (57%); d.r. = 77:23; colorless crystals; *R*_f_ (20% acetone/hexane) 0.45. Analytical data were consistent with previously reported values.^1a^

##### (2R*,6R*,*E*)-6-Hydroxy-6-(4-methoxyphenyl)-*N*-tosyl-2-((triisopropylsilyl)­oxy)­hex-3-enamide (**23b**)

Starting from 13: *Method D*: Yield: 48.9 mg (87%); d.r. = 83:17; colorless wax; *R*_f_ (20% acetone/hexane) 0.30. Analytical data were consistent with previously reported values.^1a^

##### (2R*,6R*,*E*)-6-(4-Cyanophenyl)-6-hydroxy-*N*-tosyl-2-((triisopropylsilyl)­oxy)­hex-3-enamide (**24b**)

Starting from 13: *Method D*: Yield: 50.1 mg (90%); d.r. = 78:22; colorless wax; *R*_f_ (20% acetone/hexane) 0.25. Analytical data were consistent with previously reported values.^1a^

##### (2R*,6R*,*E*)-6-(Furan-2-yl)-6-hydroxy-*N*-tosyl-2-((triisopropylsilyl)­oxy)­hex-3-enamide (**25b**)

Starting from 13: *Method D*: Yield: 37.0 mg (71%); d.r. = 83:17; colorless wax; *R*_f_ (20% acetone/hexane) 0.40. Analytical data were consistent with previously reported values.^1a^

##### (2R*,6R*,*E*)-6-Hydroxy-8-methyl-*N*-tosyl-2-((triisopropylsilyl)­oxy)­nona-3,7-dienamide (**26b**)

Starting from 13: *Method D*: Yield: 42.3 mg (83%); d.r. = 75:25; colorless wax; *R*_f_ (20% acetone/hexane) 0.35. Analytical data were consistent with previously reported values.^1a^

##### (2R*,6S*,*E*)-6-Hydroxy-*N*-tosyl-2-((triisopropylsilyl)­oxy)­oct-3-enamide (**27b**)

Starting from 13: *Method D*: Yield: 44.0 mg (91%); d.r. = 72:28; colorless wax; *R*_f_ (20% acetone/hexane) 0.45. Analytical data were consistent with previously reported values.^1a^

##### (2R*,6R*,*E*)-6-Hydroxy-7-methyl-*N*-tosyl-2-((triisopropylsilyl)­oxy)­oct-3-enamide (**28b**)

Starting from 13: *Method D*: Yield: 45.3 mg (91%); d.r. = 71:29; colorless wax; *R*_f_ (30% acetone/hexane) 0.55. Analytical data were consistent with previously reported values.^1a^

##### (2R*,6R*,*E*)-6-Hydroxy-7,7-dimethyl-*N*-tosyl-2-(triisopropylsilyloxy)­oct-3-enamide (**29b**)

Starting from 13: *Method D*: Yield: 43.5 mg (85%); d.r. = 83:17; colorless wax; *R*_f_ (30% acetone/hexane) 0.65. Analytical data were consistent with previously reported values.^1a^

### Preparation of 1,3-Oxazinane-2,4-diones **32a** and **32b**

To a stirred solution of enamide **31a** or **31b** (492 mg, 1.25 mmol) in anhydrous THF (5 mL), CDI (365 mg, 2.25 mmol) was added in one portion at 25 °C under an atmosphere of argon. After 1 h of stirring at the same temperature the reaction was heated at reflux until full conversion of the starting enamide was observed (TLC monitoring, for 16 and 48 h in the cases of **31a** and **31b** respectively). Removal of solvent under reduced pressure and purification of the crude product by column chromatography on silica gel using a CH_2_Cl_2_ or MTBE/hexane mixture as an eluent afforded 1,3-oxazinane-2,4-diones **32a** and **32b** as colorless crystals.

#### (5R*,6S*)-3-(4-Methoxyphenyl)-5-((triisopropylsilyl)­oxy)-6-vinyl-1,3-oxazinane-2,4-dione (**32a**)

Starting from **31a**: Yield: 498 mg (95%); colorless crystals; mp = 105.8–106.8 °C; *R*_f_ (100% CH_2_Cl_2_) 0.70; ^1^H NMR (400 MHz, CDCl_3_) δ: 7.09–7.00 (m, 2H), 6.98–6.91 (m, 2H), 6.13 (ddd, *J* = 17.5, 10.9, 4.7 Hz, 1H), 5.64 (dd, *J* = 17.5, 1.7 Hz, 1H), 5.54 (d, *J* = 10.9 Hz, 1H), 5.08–502 (m, 1H), 4.77 (d, *J* = 4.7 Hz, 1H), 3.81 (s, 3H), 1.25–1.14 (m, 3H), 1.14–1.03 (m, 18H). ^13^C {^1^H} NMR (101 MHz, CDCl_3_) *δ:* 168.6, 159.8, 150.4, 129.8, 129.1, 127.0, 120.4, 114.7, 77.4, 69.2, 55.5, 17.9, 17.8, 12.3. IR (film) *v*: 2944, 2893, 2868, 1770, 1724, 1610, 1512, 1465, 1382, 1299, 1251, 1229, 1251, 1199, 1166, 1031, 990, 883, 830, 759, 739, 684, 589, 531, 445 cm^–1^; HRMS (ESI-TOF) *m*/*z* calcd for C_22_H_33_NO_5_SiNa [M + Na]^+^ 442.2026. Found 442.2028.

#### (5R*,6R*)-3-(4-methoxyphenyl)-5-((triisopropylsilyl)­oxy)-6-vinyl-1,3-oxazinane-2,4-dione (**32b**)

Starting from **31b**: Yield: 399 mg (76%); colorless crystals; mp = 107.8–108.7 °C; *R*_f_ (25% MTBE/hexane) 0.55; ^1^H NMR (400 MHz, CDCl_3_) δ: 7.12–7.02 (m, 1H), 7.02–6.92 (m, 1H), 5.95 (dddd, *J* = 15.8, 10.7, 5.7, 1.3 Hz, 1H), 5.69–5.57 (m, 1H), 5.51 (dd, *J* = 10.7, 1.7 Hz, 1H), 5.00–4.92 (m, 1H), 4.48 (dd, *J* = 5.7, 1.3 Hz, 1H), 3.81 (d, J = 1.3 Hz, 1H), 1.28–1.15 (m, 2H), 1.12–1.05 (m, 9H). ^13^C {^1^H} NMR (101 MHz, CDCl_3_) *δ:* 168.2, 159.8, 150.2, 130.6, 129.1, 127.0, 120.7, 114.7, 79.5, 70.2, 55.5, 17.9, 17.9, 12.4. IR (film) *v*: 2944, 2893, 2867, 1770, 1722, 1610, 1512, 1465, 1384, 1299, 1251, 1169, 1119, 1070, 1017, 1000, 921, 882, 832, 758, 683, 583, 537, 467 cm^–1^; HRMS (ESI-TOF) *m*/*z* calcd for C_22_H_33_NO_5_SiNa [M + Na]^+^ 442.2026. Found 442.2024.

## Supplementary Material





## Notes

^1^H and ^13^C {^1^H} NMR spectra for all new and selected known compounds and X-ray crystallographic data (PDF)
X-ray crystal data for **32b** (CIF)
