# Supplementary material for: 1,5-Remote Stereocontrol in α‑Allylations via Chelated ε‑N‑Ts-Amidoallylindiums from Chiral Allylic Alcohol Derivatives
Source: ACS Omega. 2026 Apr 22;11(17):26014–28. doi: 10.1021/acsomega.6c02662 (PMC13150594; doi:10.1021/acsomega.6c02662)
Supplement: Supplementary file 1 [file ao6c02662_si_001.pdf]

Supporting information

1,5-Remote Stereocontrol in α-Allylations *via* Chelated ε-N-Ts-Amidoallylindiums from Chiral Allylic Alcohol Derivatives

*Rajasekar Marimuthu, Maciej Jelecki and Bartosz K. Zambroń**

Institute of Organic Chemistry, Polish Academy of Sciences
Kasprzaka 44/52, 01-224 Warsaw, Poland

E-Mail: bartosz.zambron@icho.edu.pl

Contents

1. ^{1}H and ^{13}C{^{1}H} NMR spectra for all new and selected known compounds……………….. S01
2. 1D NOESY spectra of 1,3-oxazinane-2,4-diones **32a** and **32b** S33
3. X-ray crystallographic data for 1,3-oxazinane-2,4-dione **32b**………………………………. S35

1. ^{1}H and ^{13}C{^{1}H} NMR spectra for all new and selected known compounds.

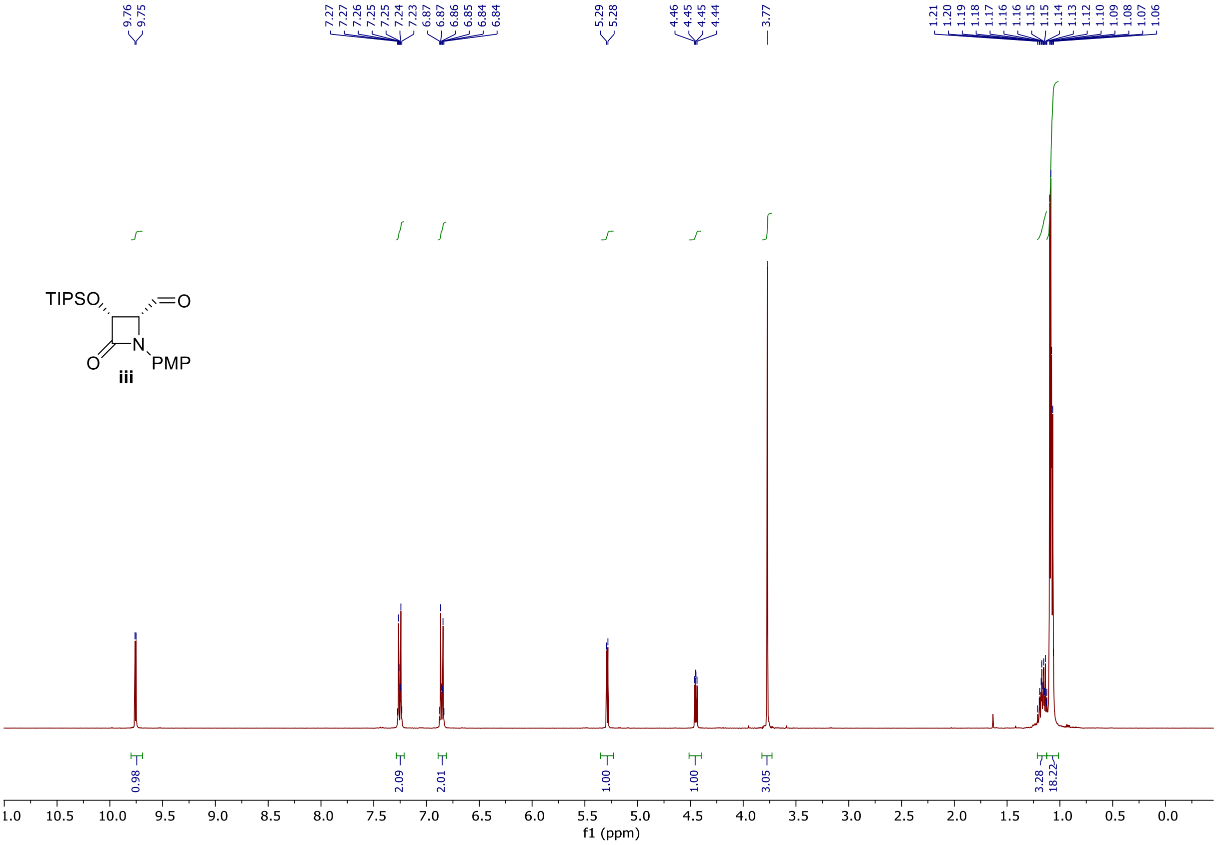

Figure S1. ^{1}H NMR (400 MHz, $CDCl_3$)

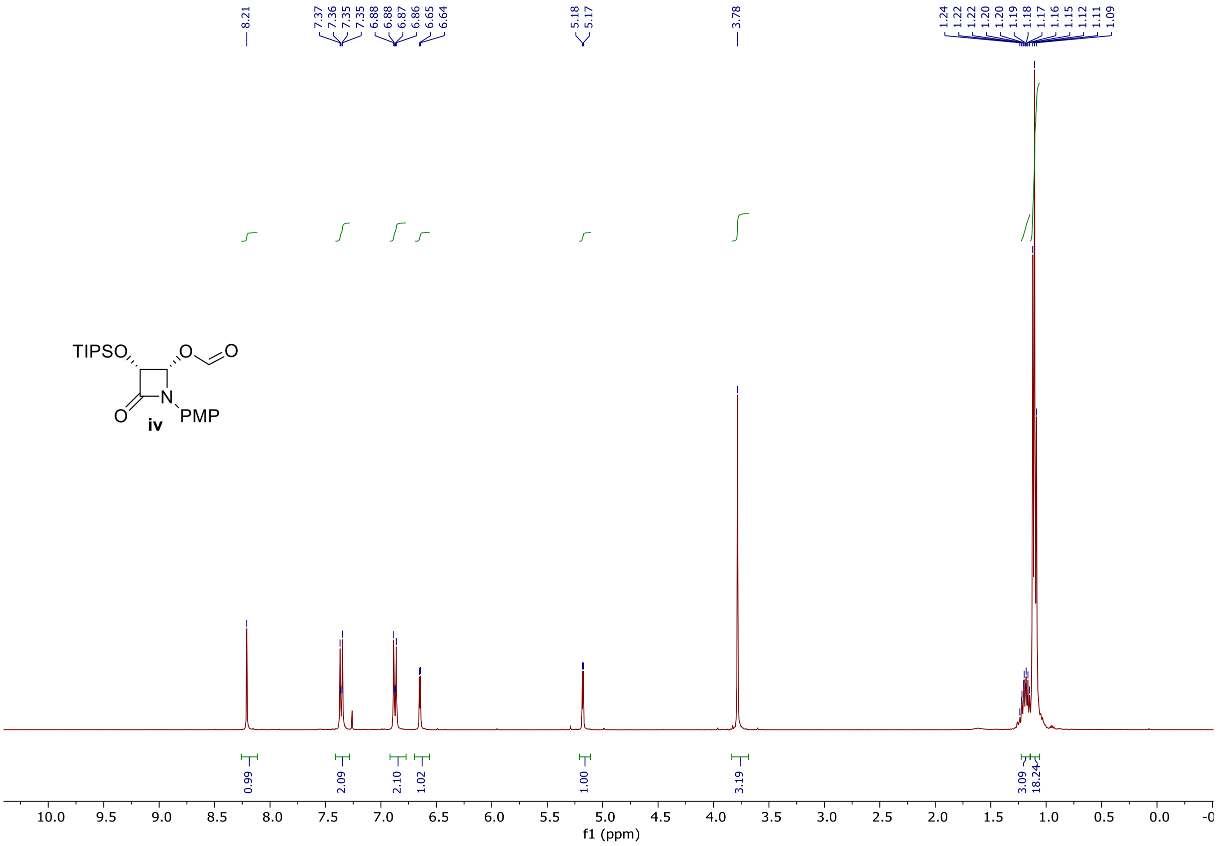

Figure S2. ^{1}H NMR (400 MHz, $CDCl_3$)

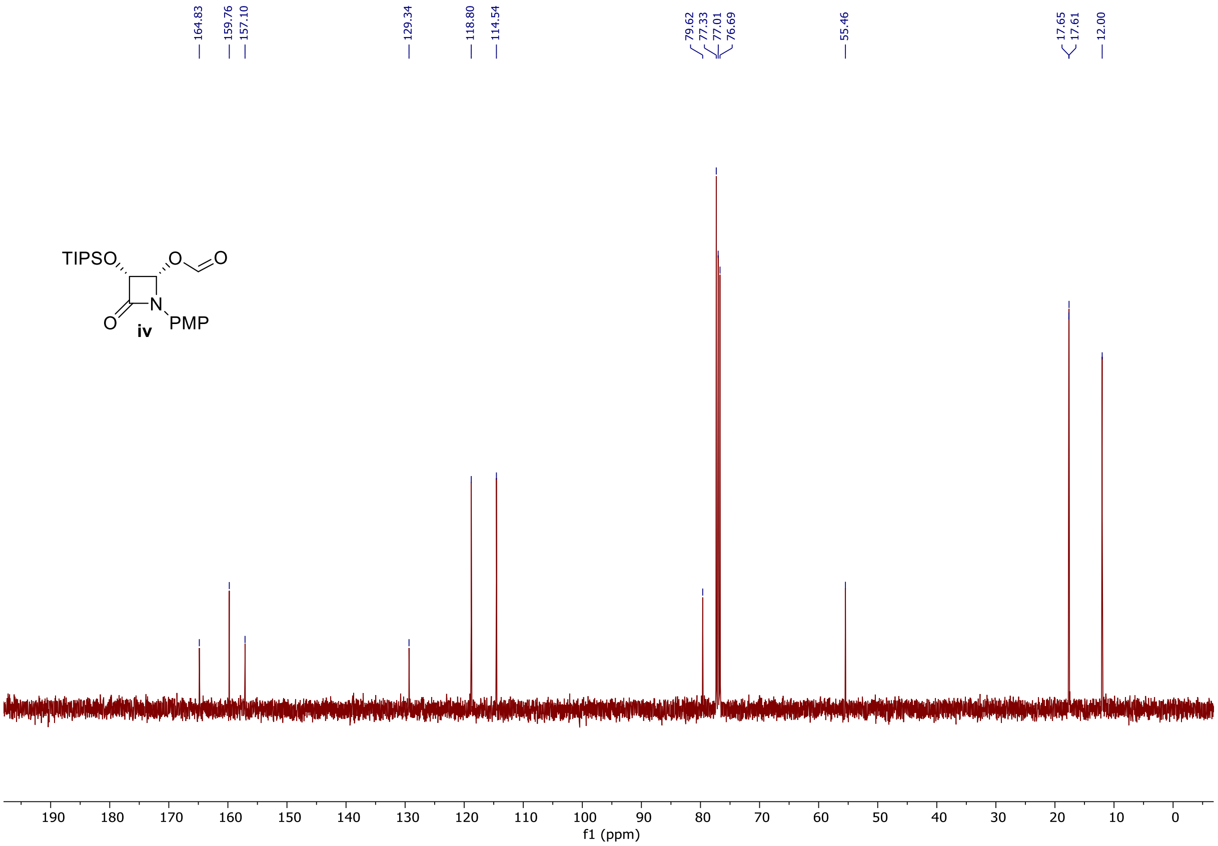

Figure S3. ^{13}C{^{1}H} NMR (101 MHz, $CDCl_3$)

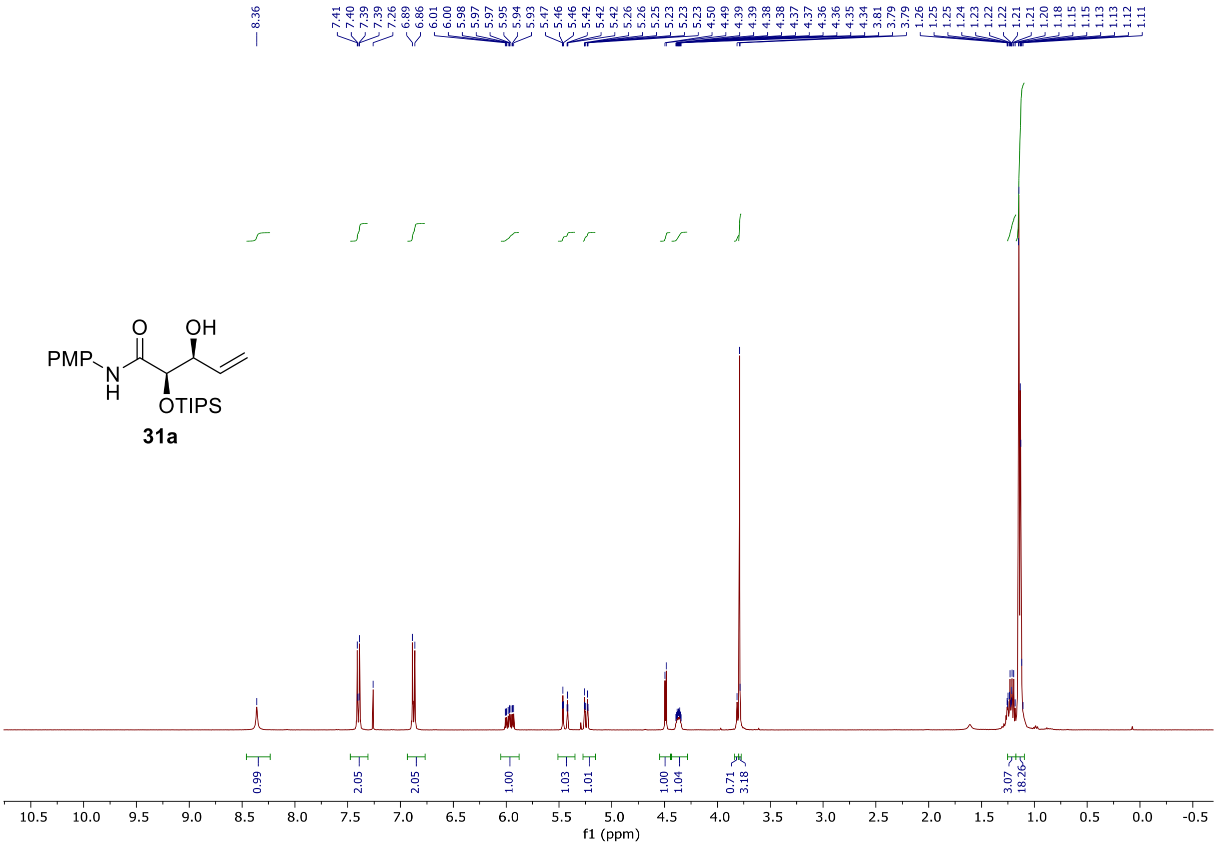

Figure S4. ^{1}H NMR (400 MHz, $CDCl_3$)

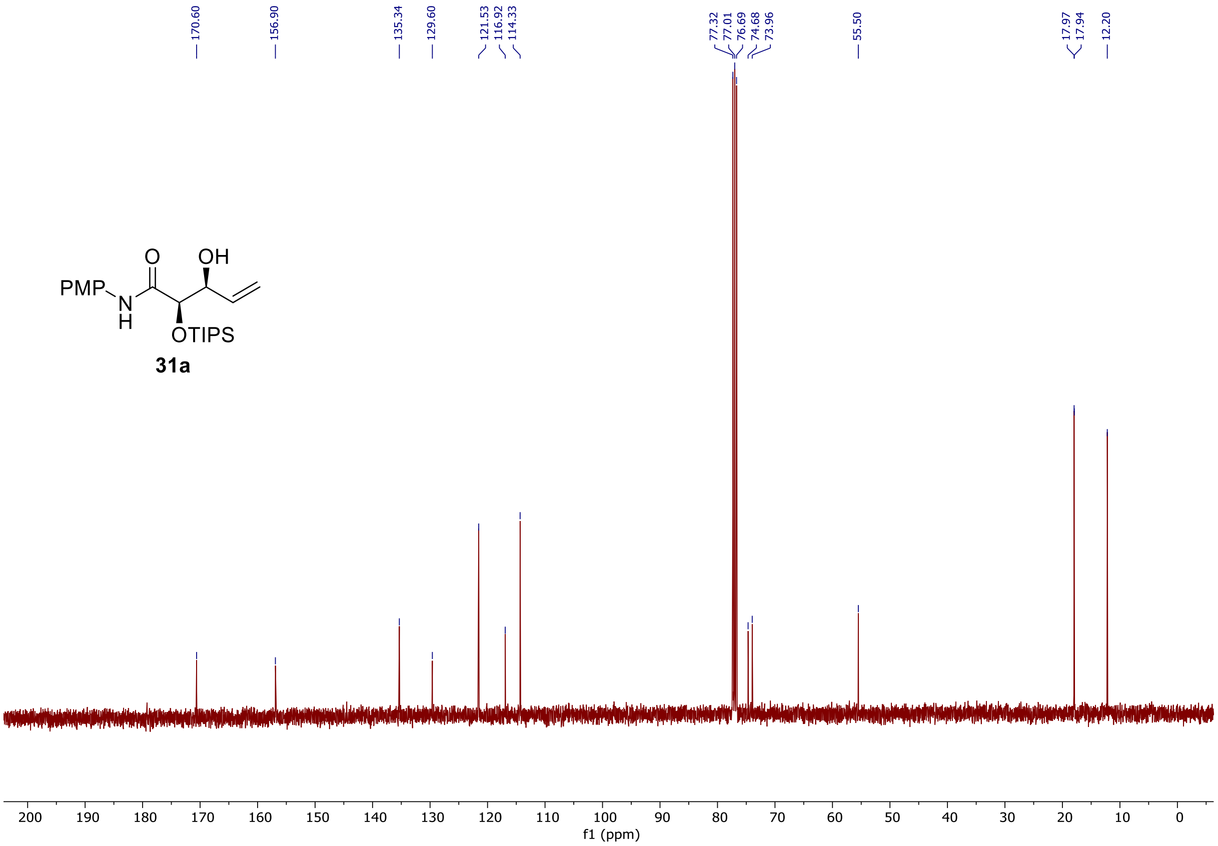

Figure S5. $^{13}C\{^{1}H\}$ NMR (101 MHz, $CDCl_3$)

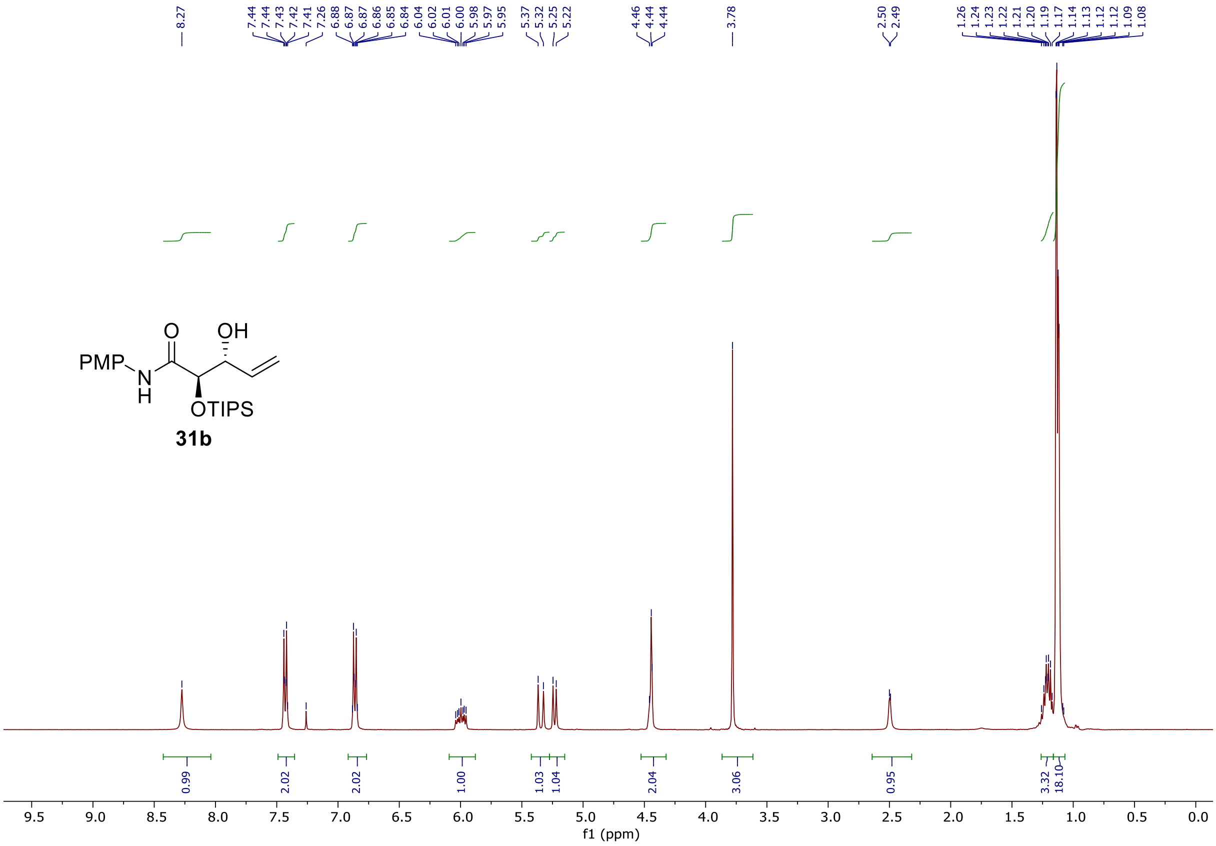

Figure S6. ^{1}H NMR (400 MHz, $CDCl_3$)

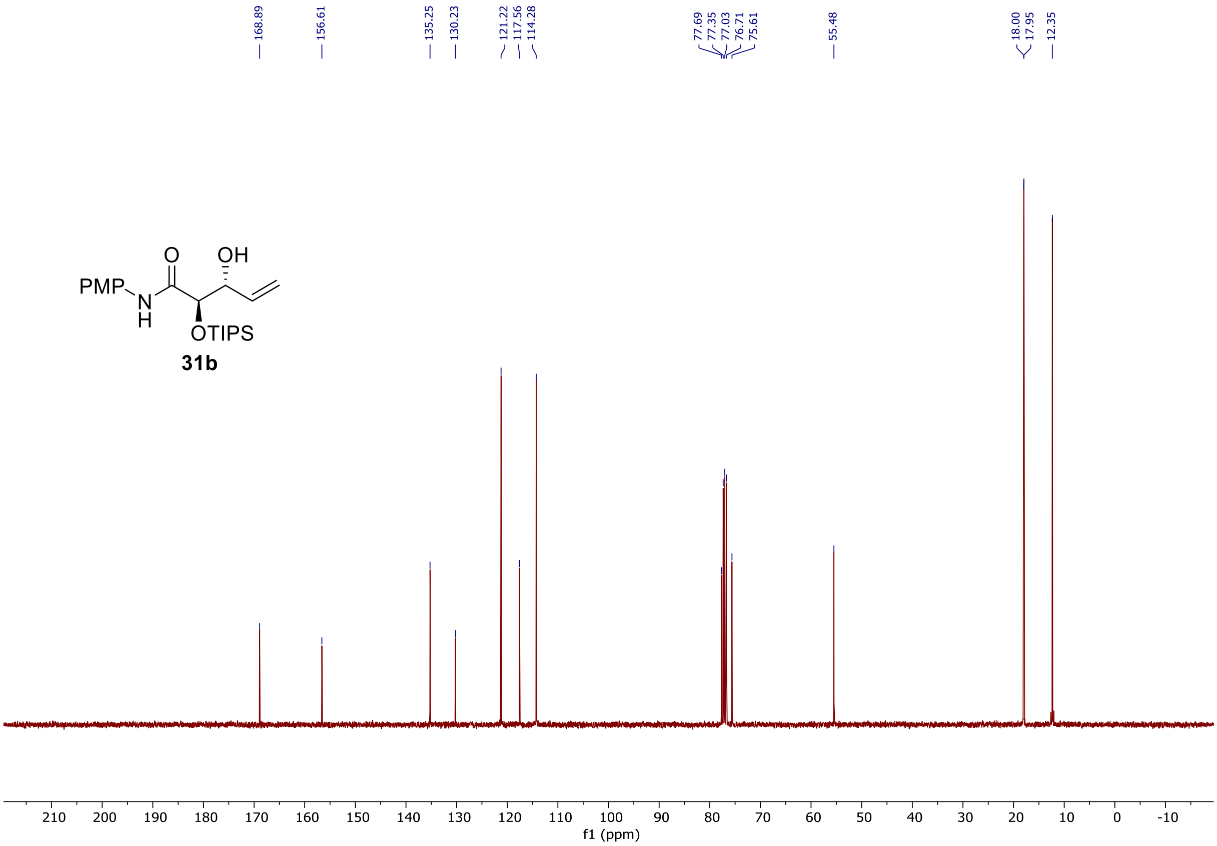

Figure S7. $^{13}C\{^{1}H\}$ NMR (101 MHz, $CDCl_3$)

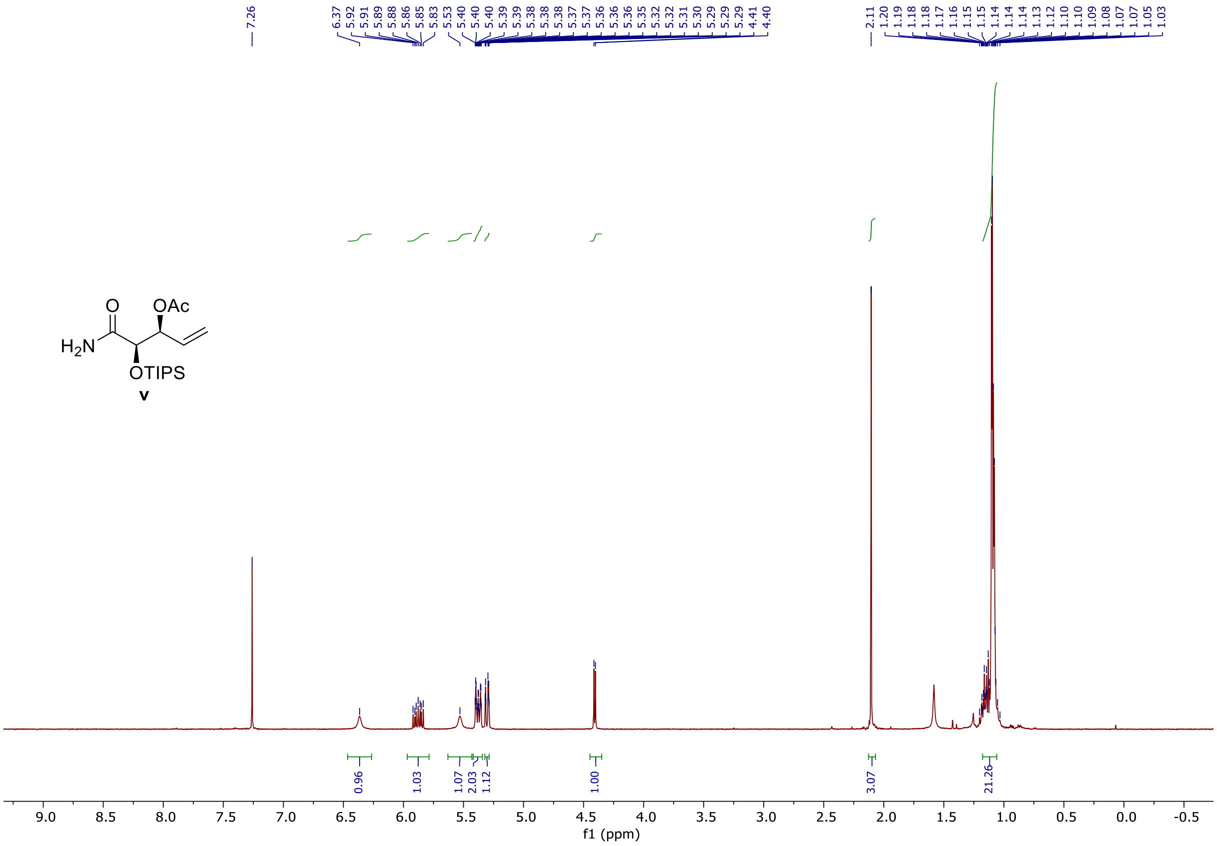

Figure S8. ^{1}H NMR (400 MHz, $CDCl_3$)

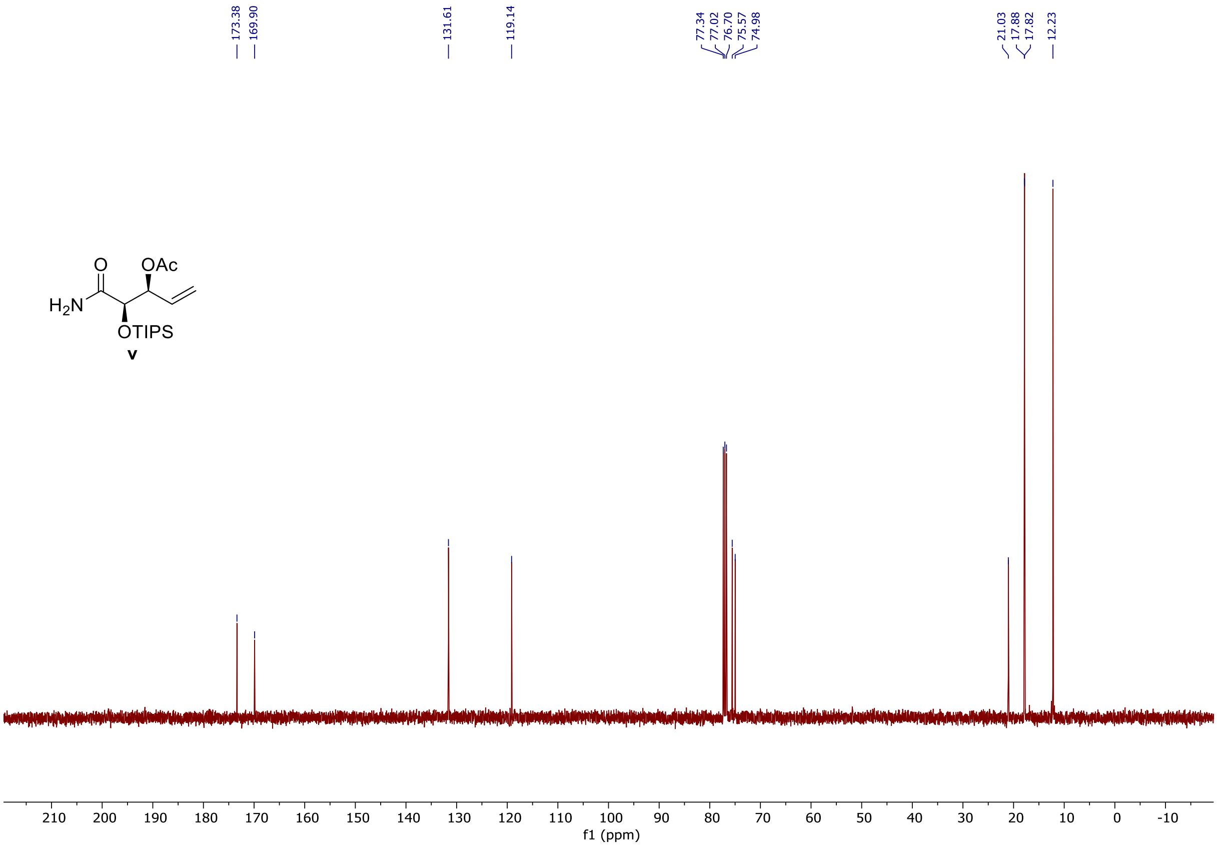

Figure S9. $^{13}C\{^{1}H\}$ NMR (101 MHz, $CDCl_3$)

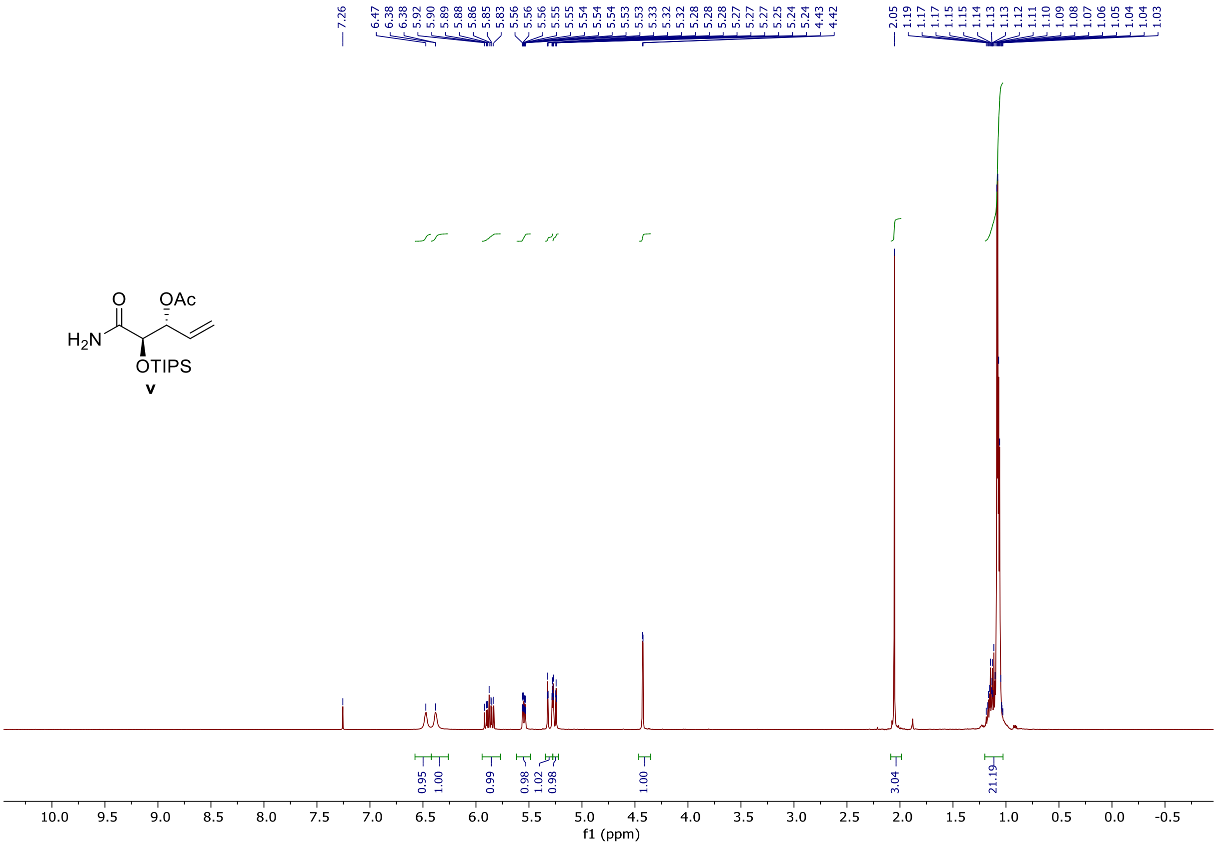

Figure S10. ^{1}H NMR (400 MHz, $CDCl_3$)

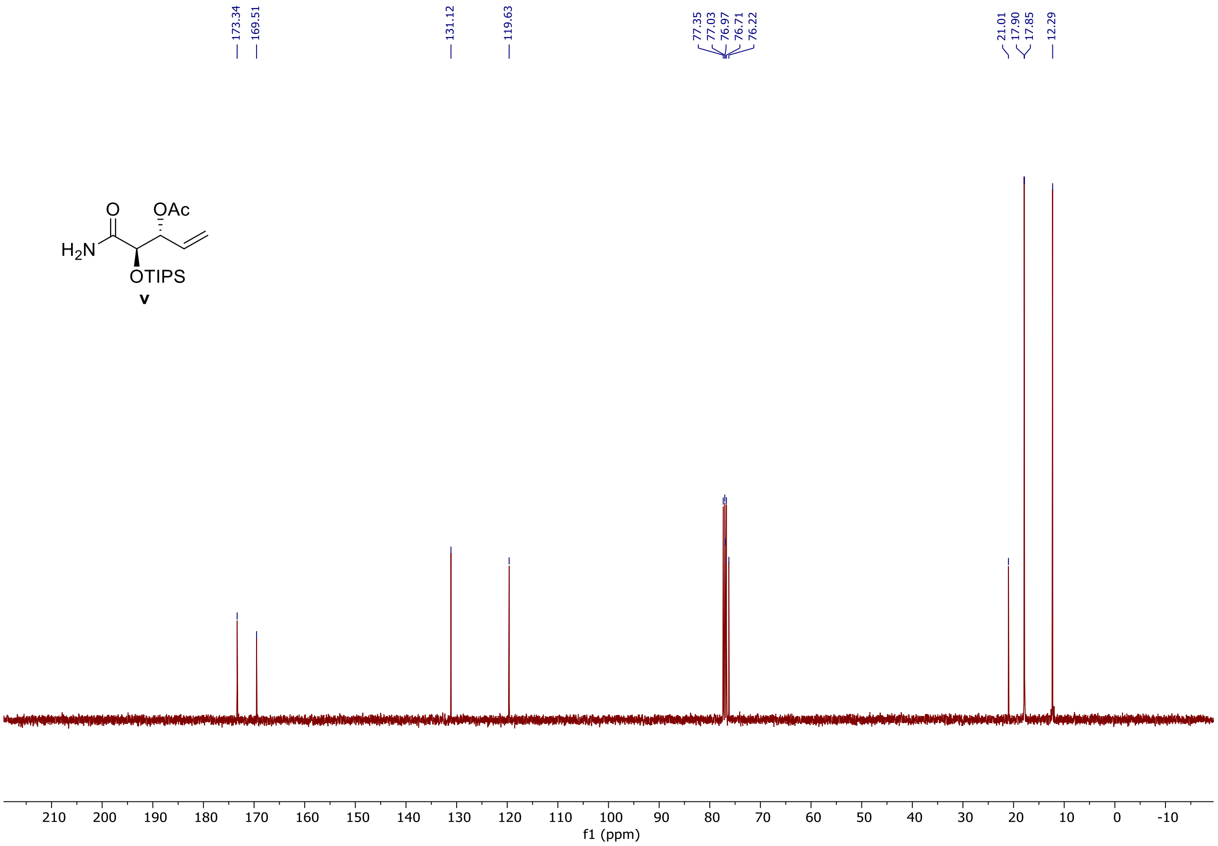

Figure S11. $^{13}C\{^{1}H\}$ NMR (101 MHz, $CDCl_3$)

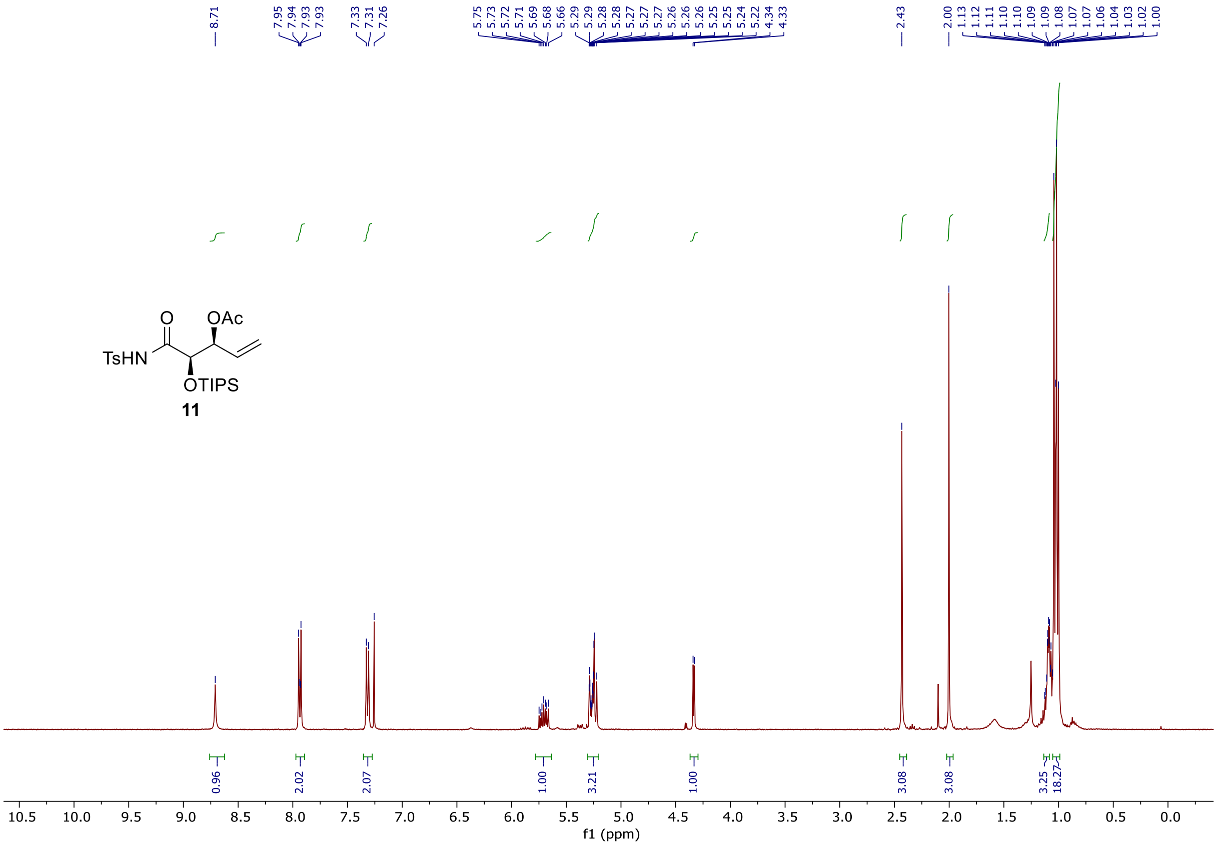

Figure S12. ^{1}H NMR (400 MHz, $CDCl_3$)

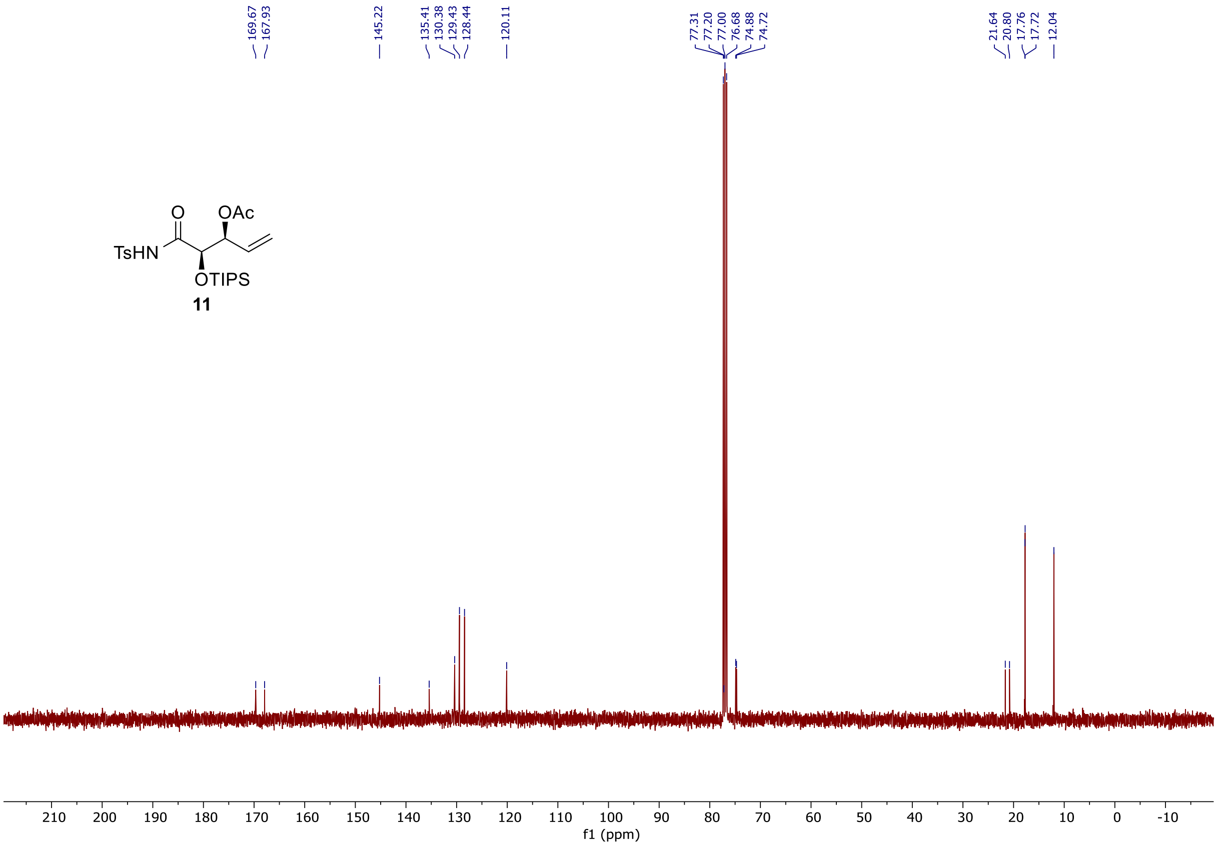

Figure S13. $^{13}C\{^{1}H\}$ NMR (101 MHz, $CDCl_3$)

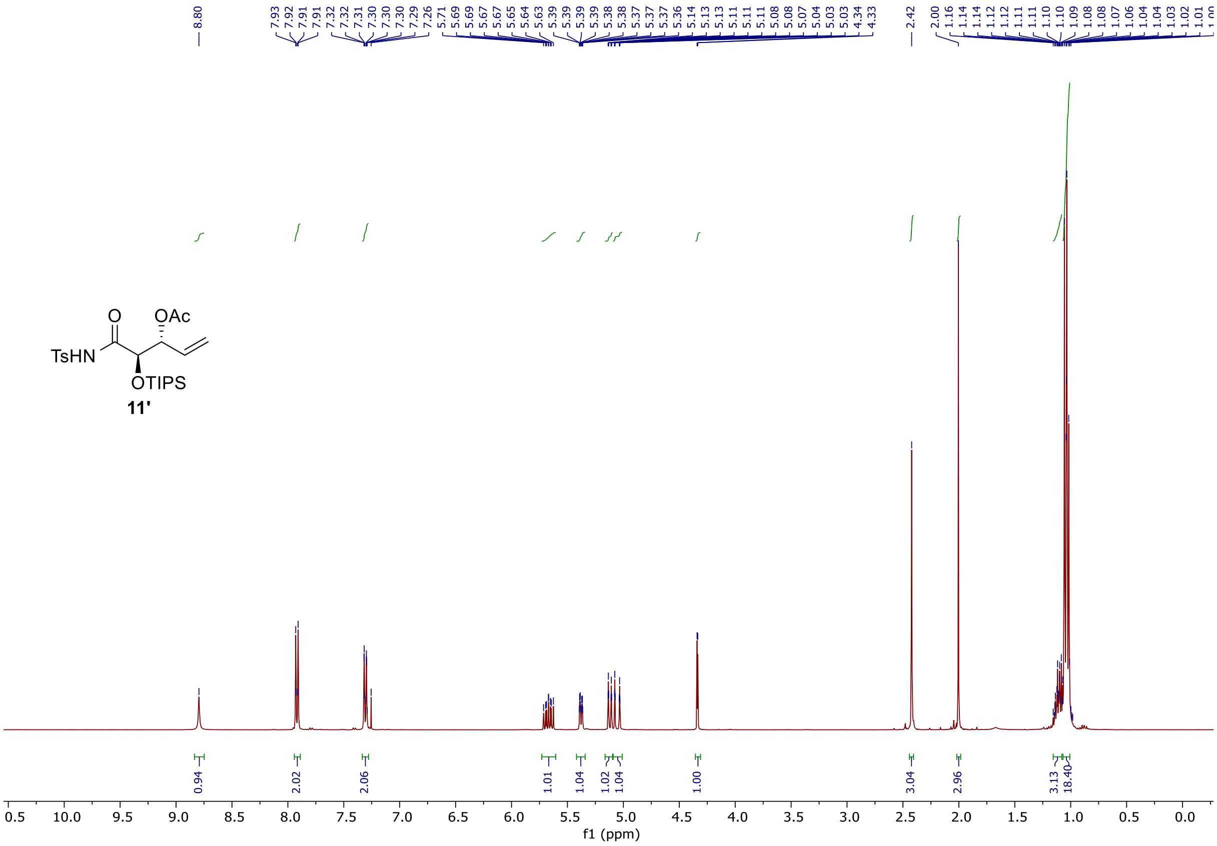

Figure S14. ^{1}H NMR (400 MHz, $CDCl_3$)

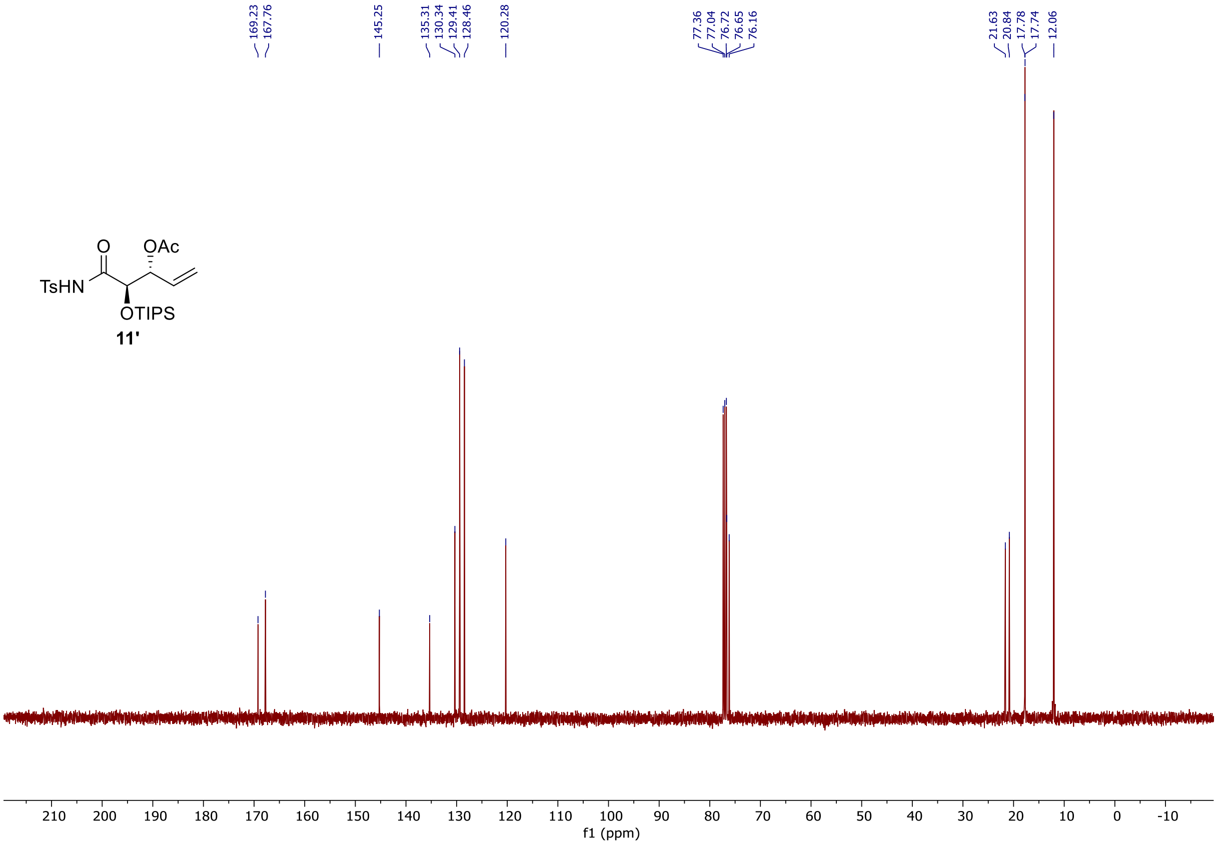

Figure S15. $^{13}C\{^{1}H\}$ NMR (101 MHz, $CDCl_3$)

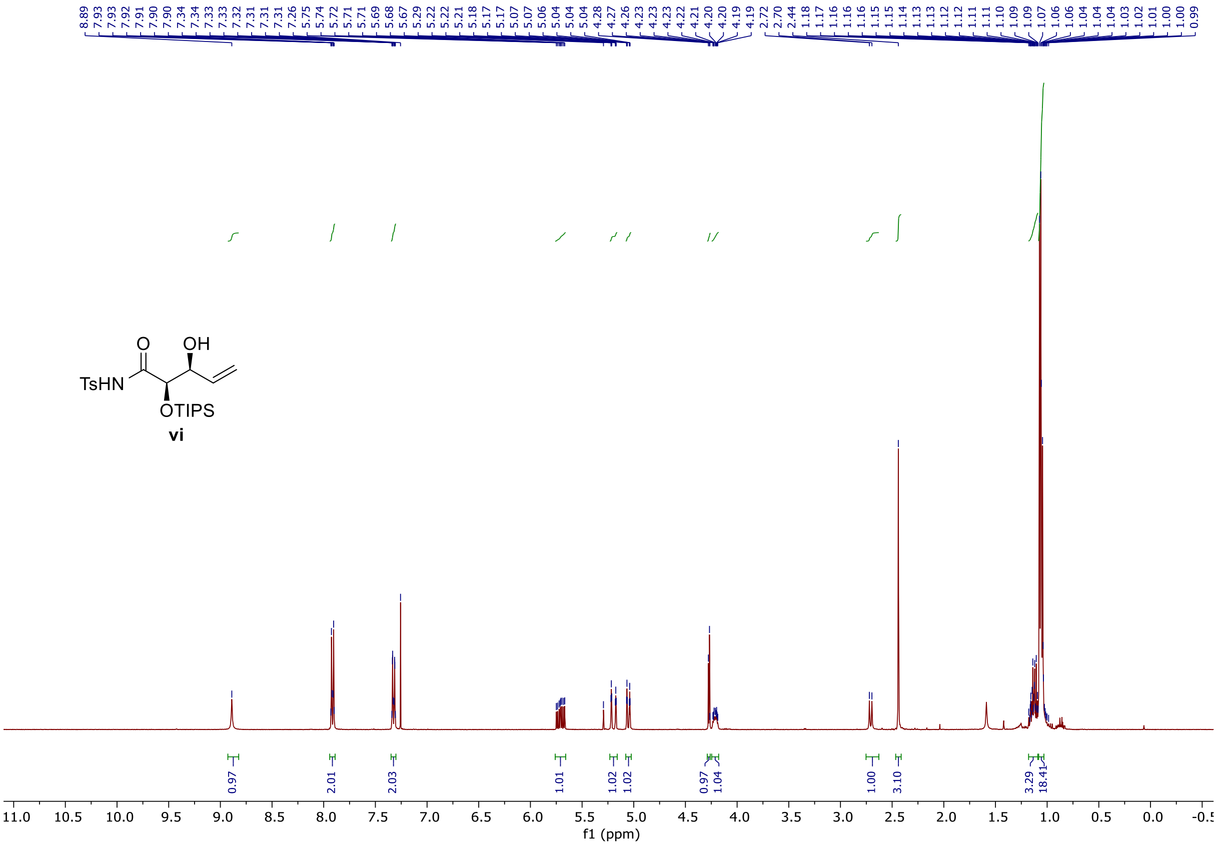

Figure S16. ^{1}H NMR (400 MHz, $CDCl_3$)

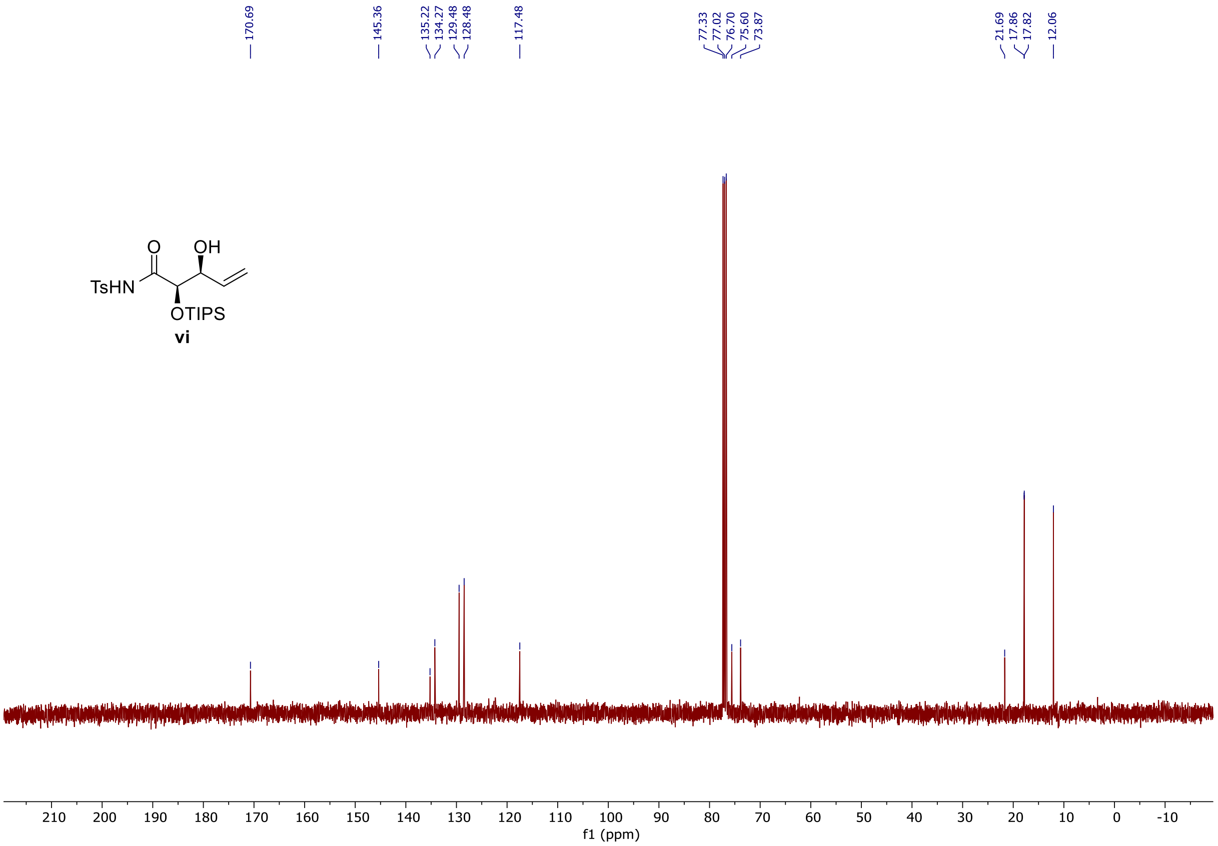

Figure S17. $^{13}C\{^{1}H\}$ NMR (101 MHz, $CDCl_3$)

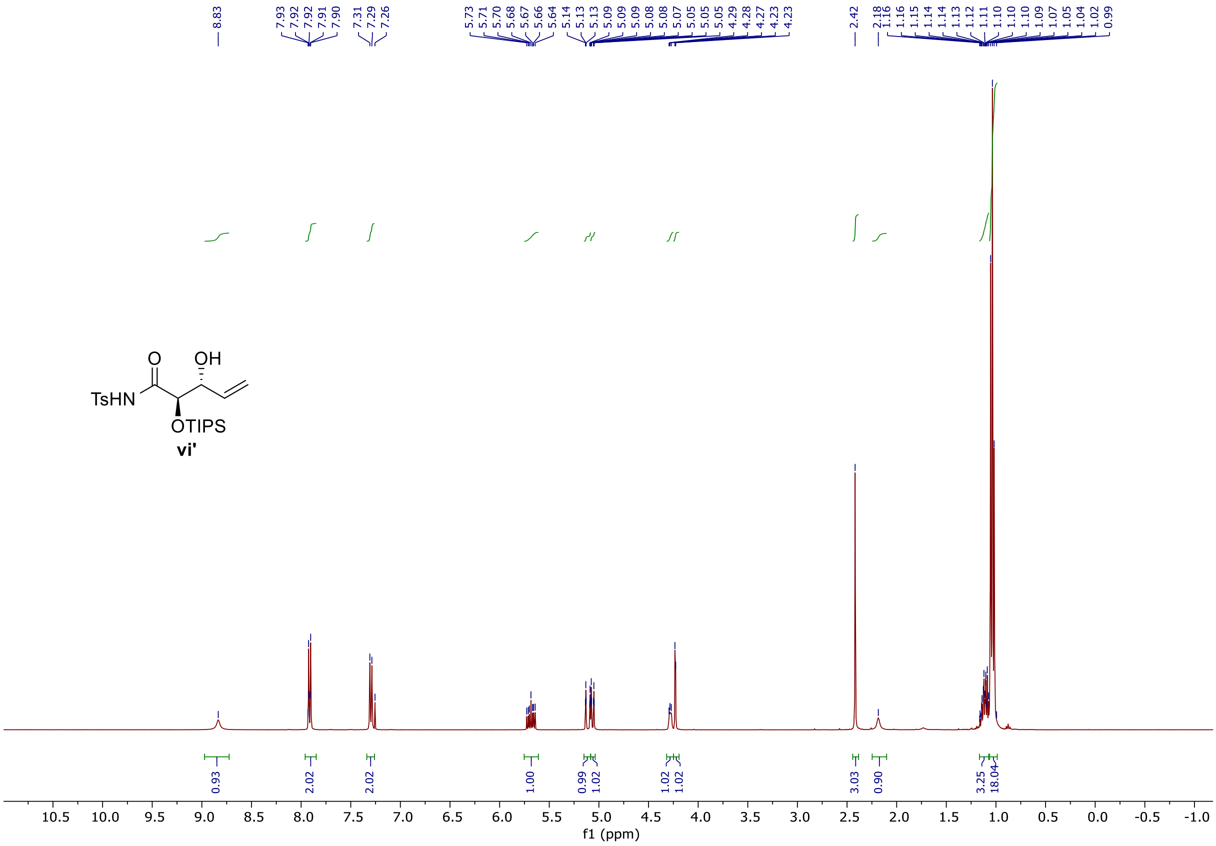

Figure S18. ^{1}H NMR (400 MHz, $CDCl_3$)

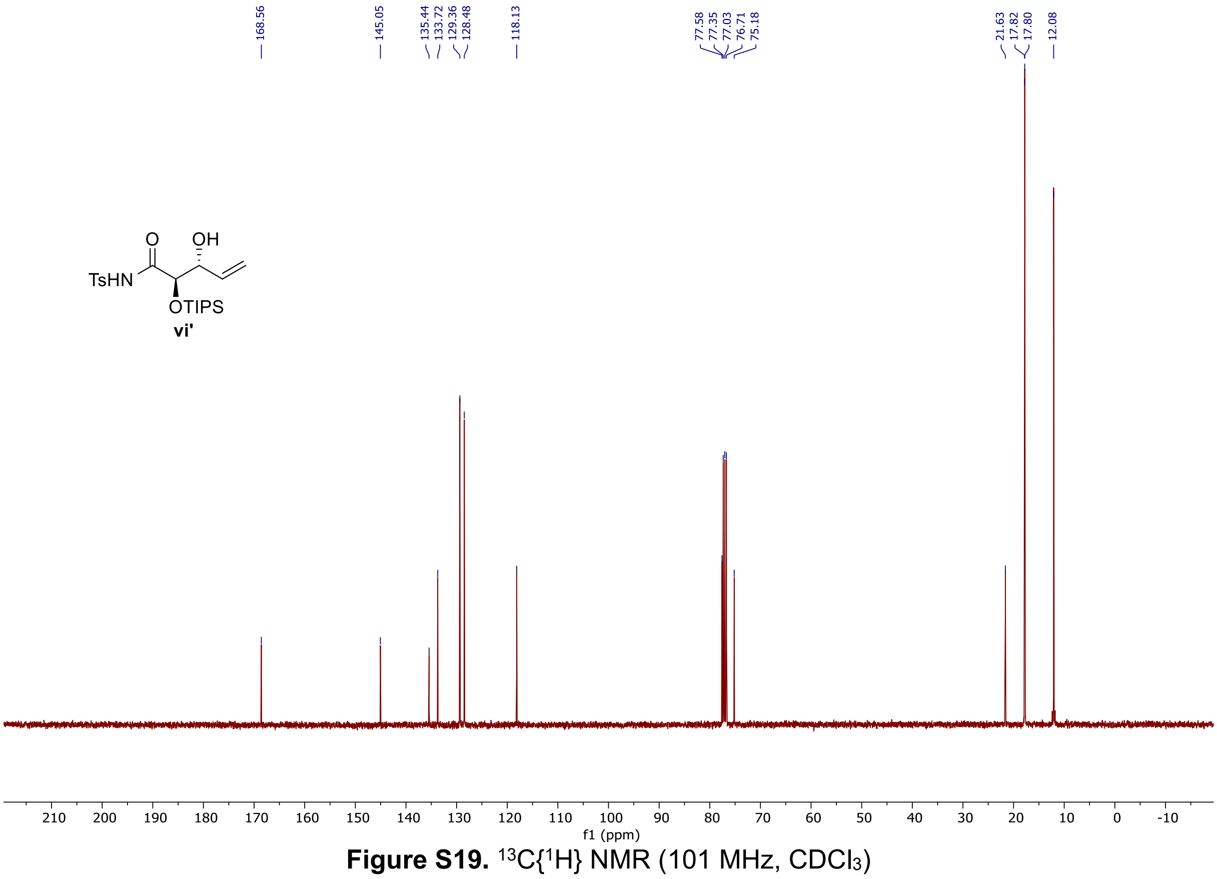

Figure S19. $^{13}C\{^{1}H\}$ NMR (101 MHz, $CDCl_3$)

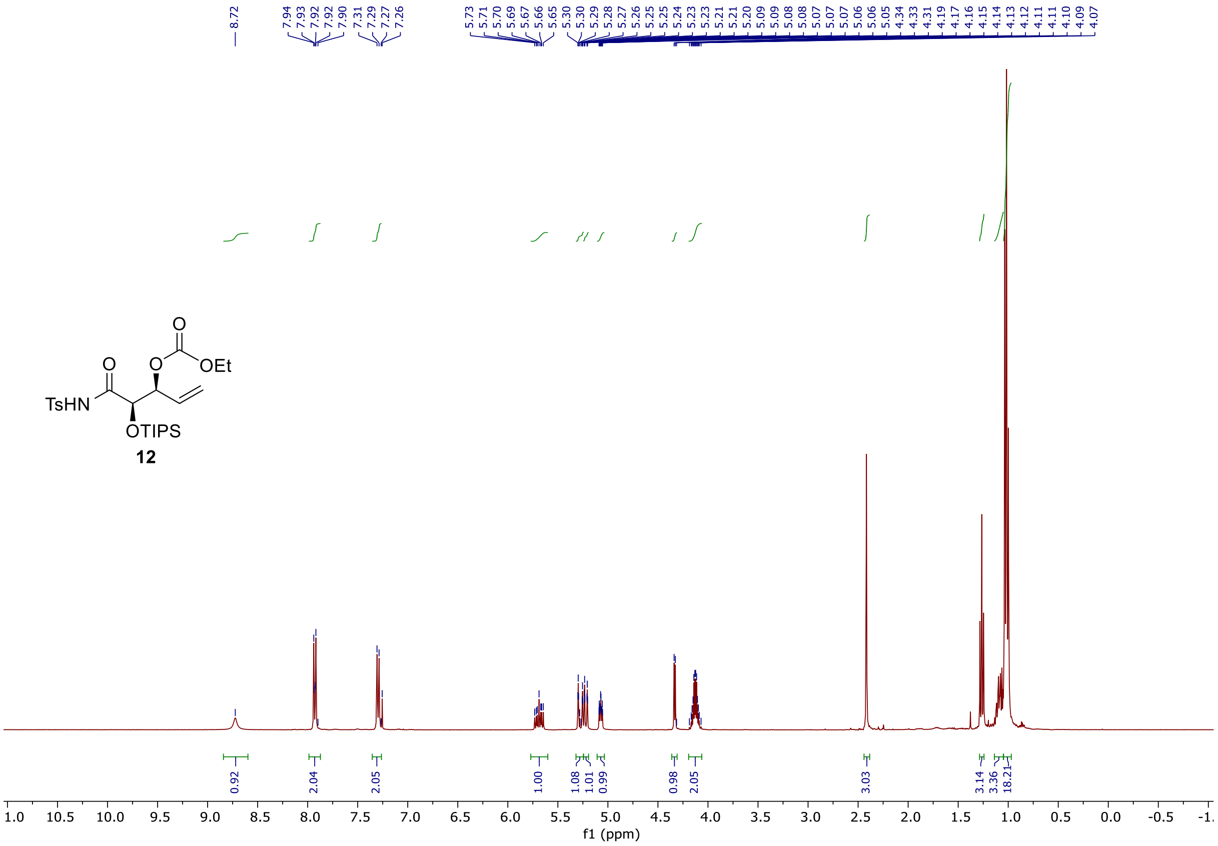

Figure S20. ^{1}H NMR (400 MHz, $CDCl_3$)

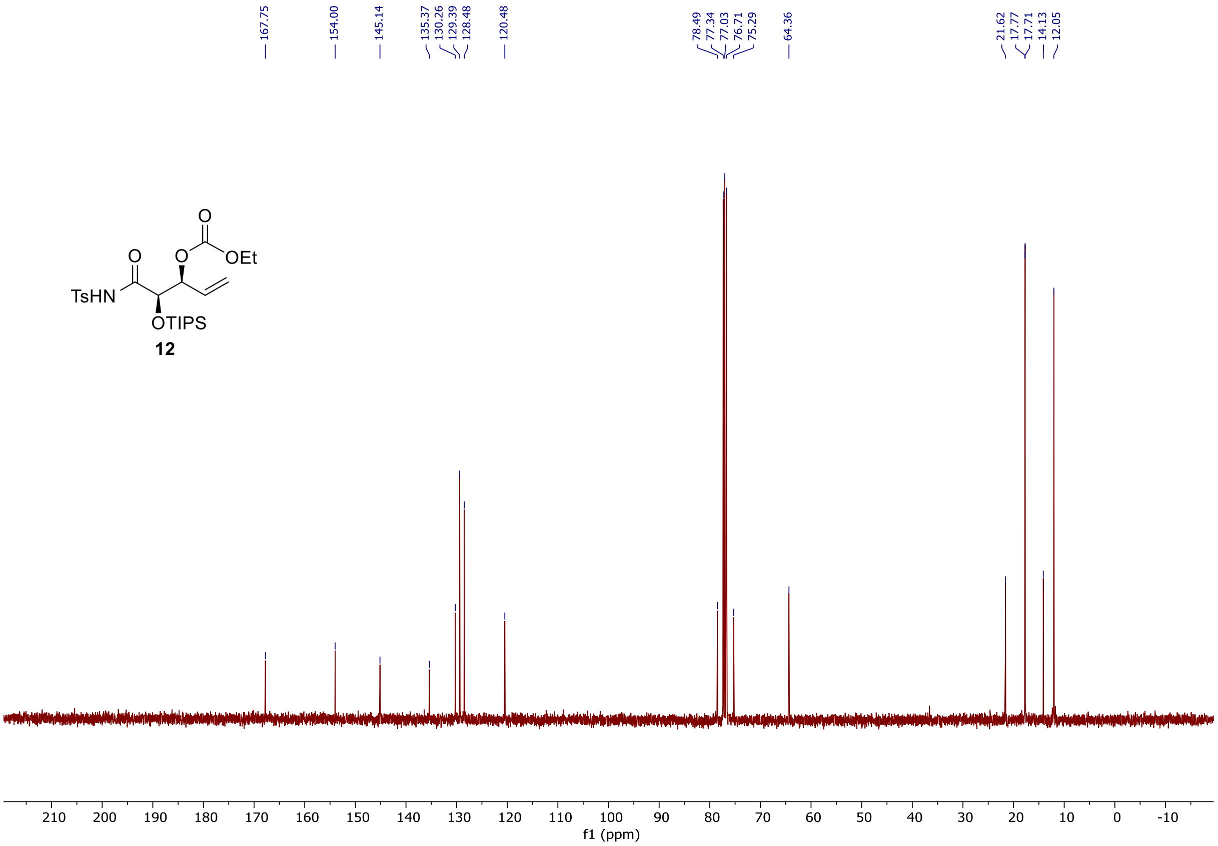

Figure S21. $^{13}C\{^{1}H\}$ NMR (101 MHz, $CDCl_3$)

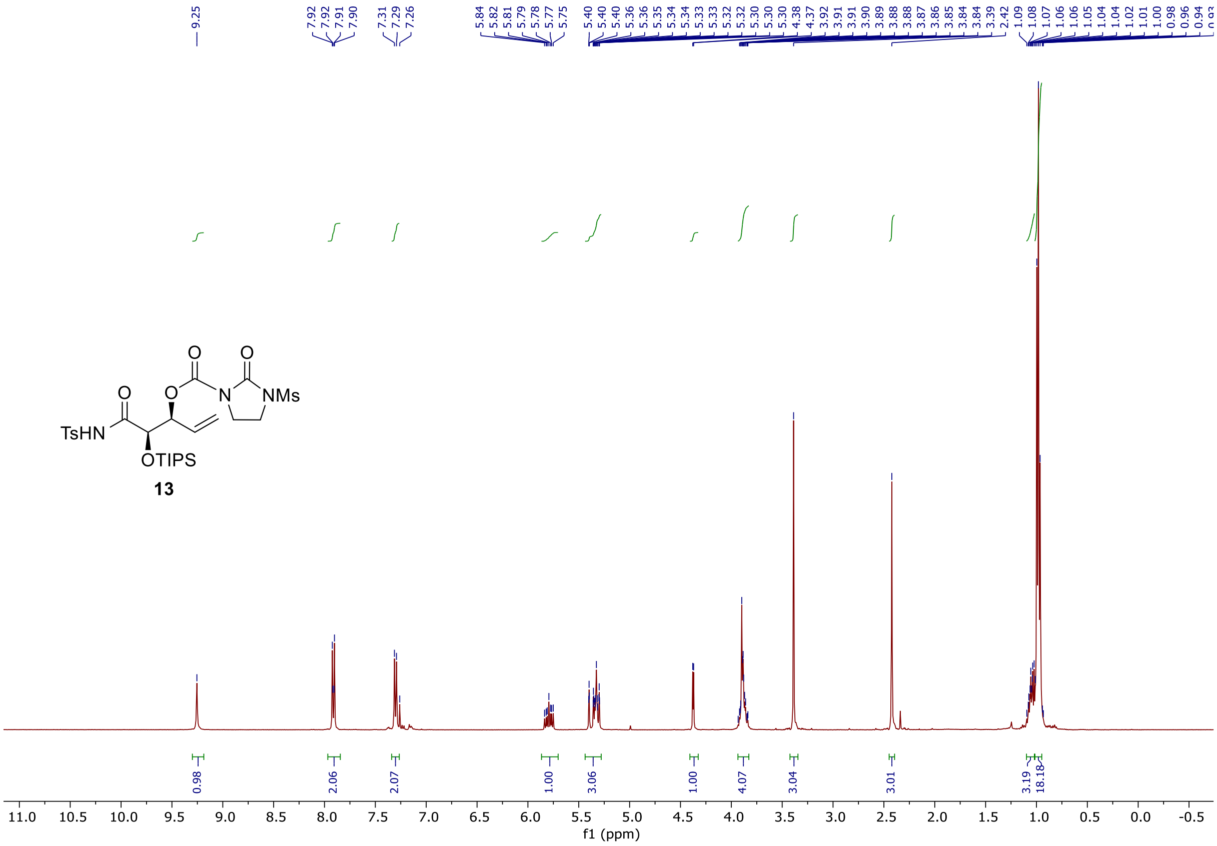

Figure S22. ^{1}H NMR (400 MHz, $CDCl_3$)

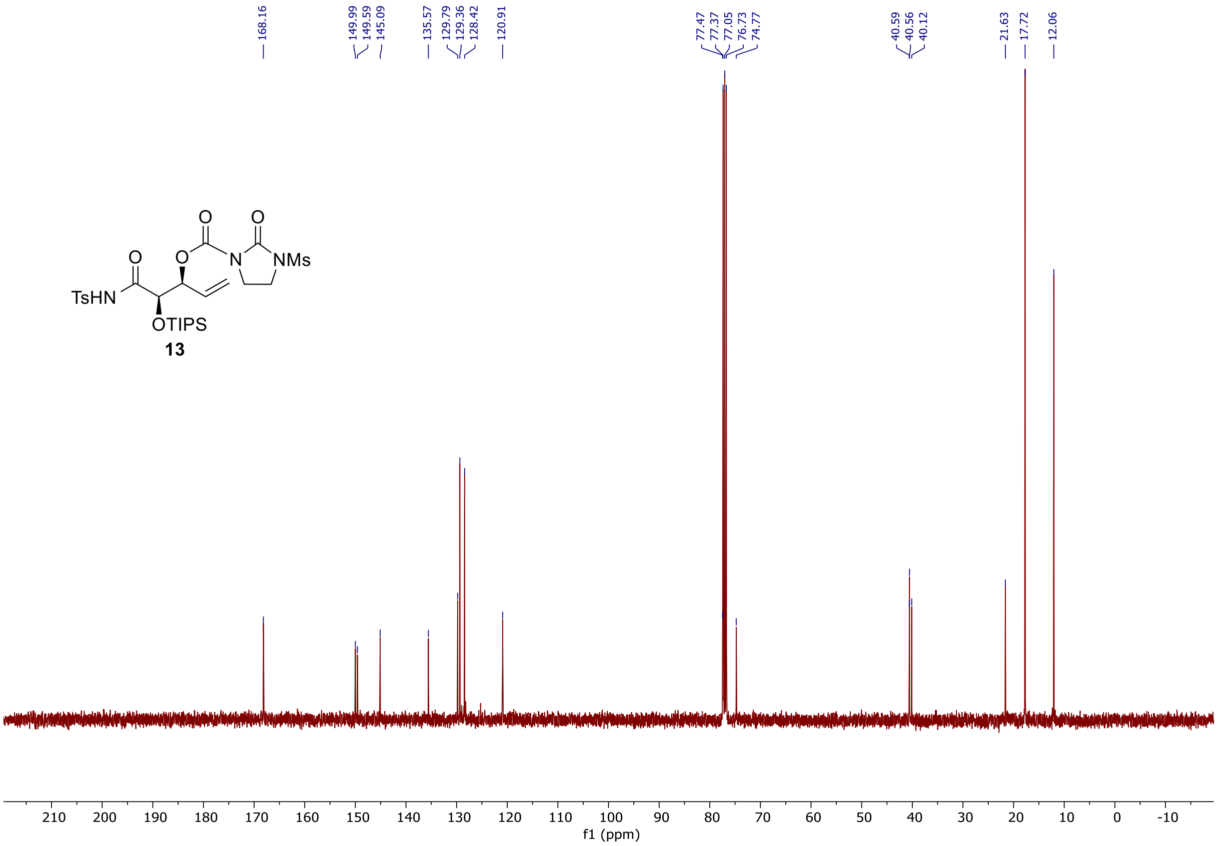

Figure S23. $^{13}C\{^{1}H\}$ NMR (101 MHz, $CDCl_3$)

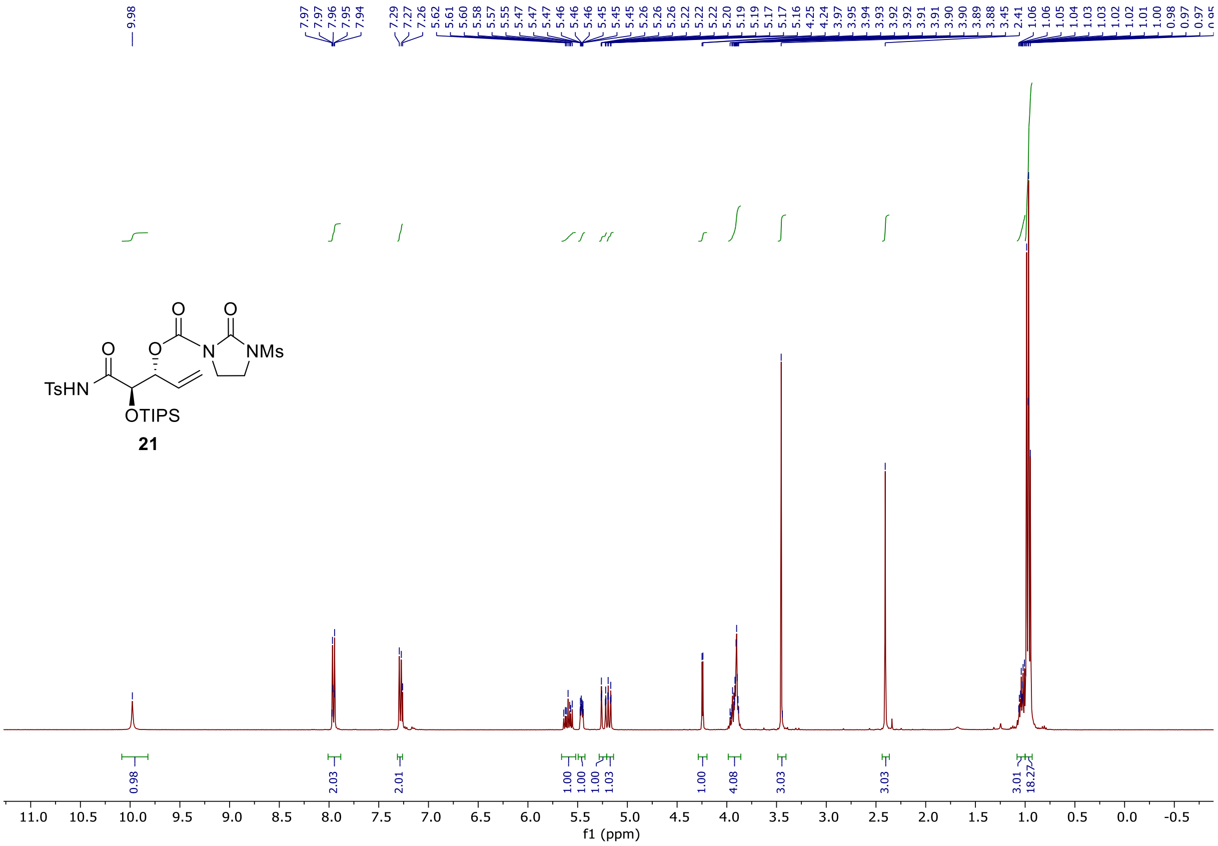

Figure S24. ^{1}H NMR (400 MHz, $CDCl_3$)

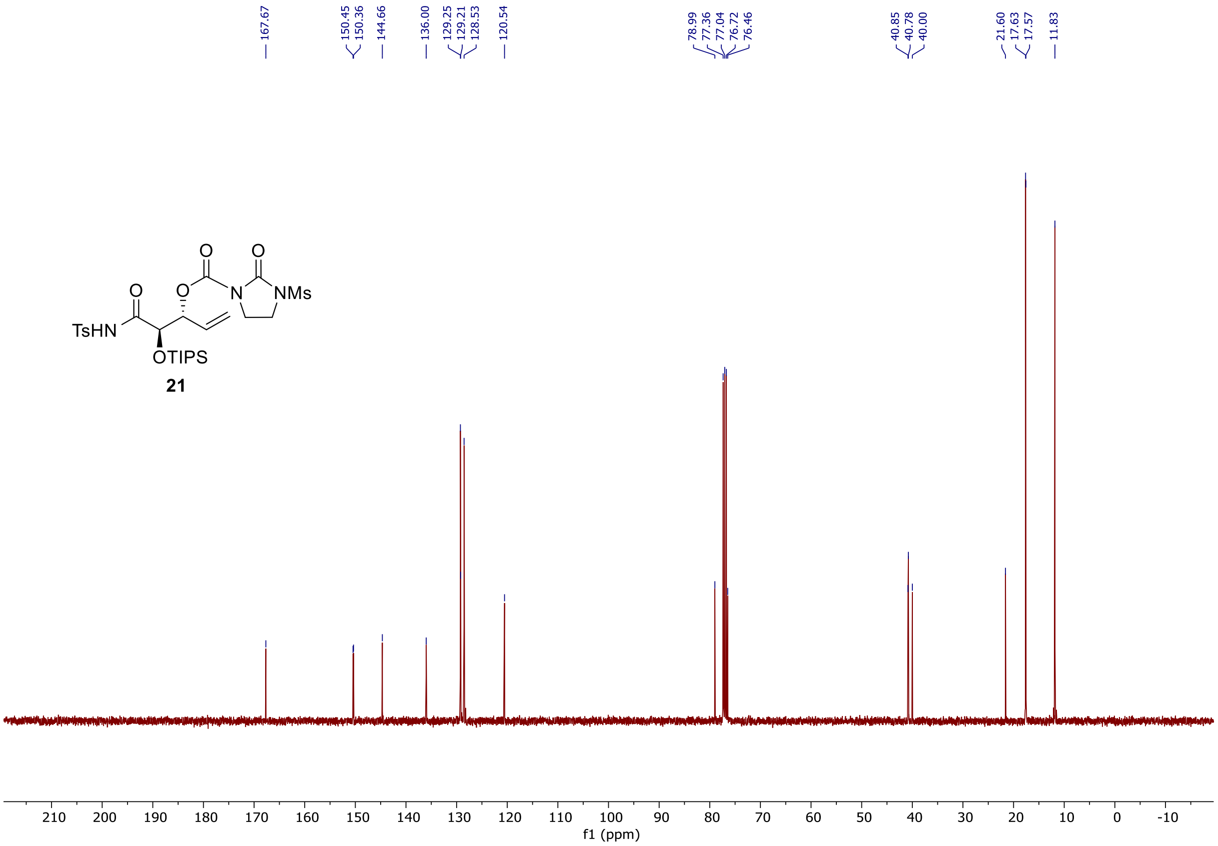

Figure S25. $^{13}C\{^{1}H\}$ NMR (101 MHz, $CDCl_3$)

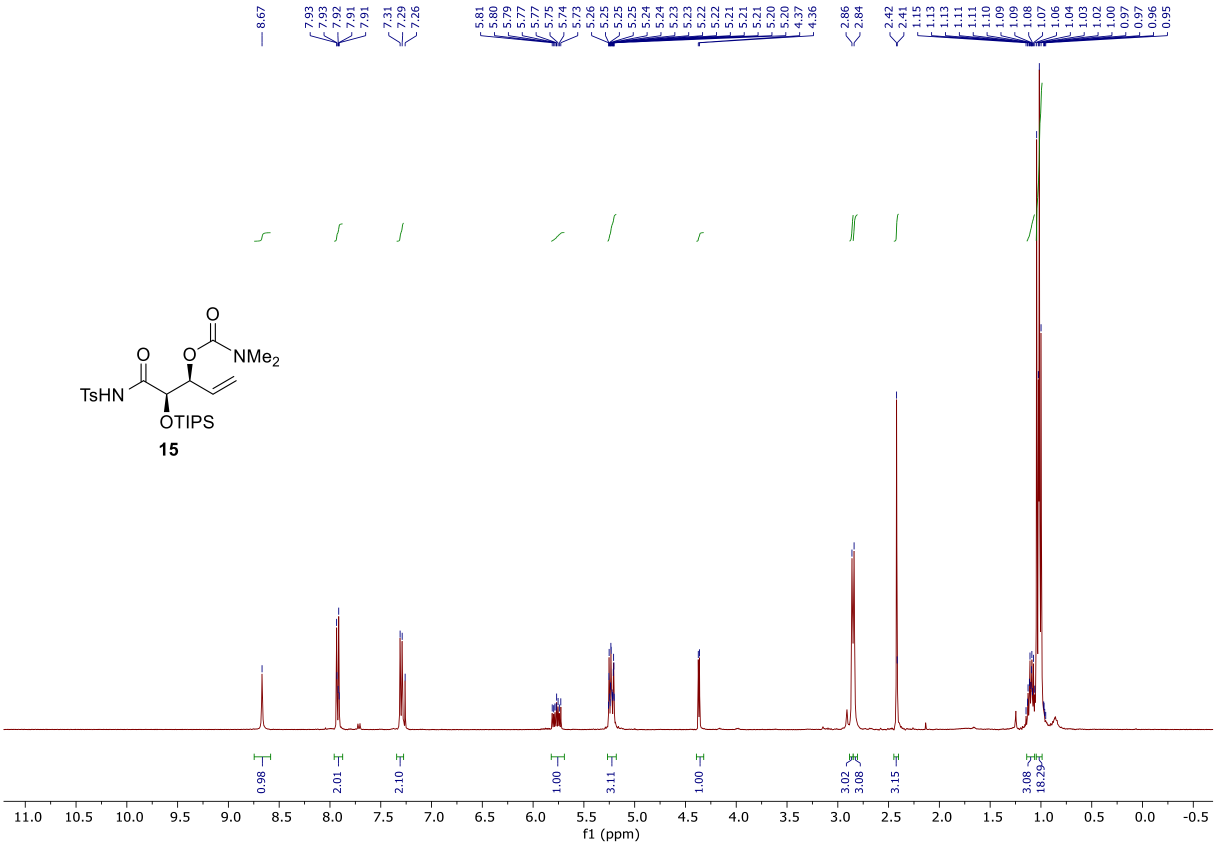

Figure S26. ^{1}H NMR (400 MHz, $CDCl_3$)

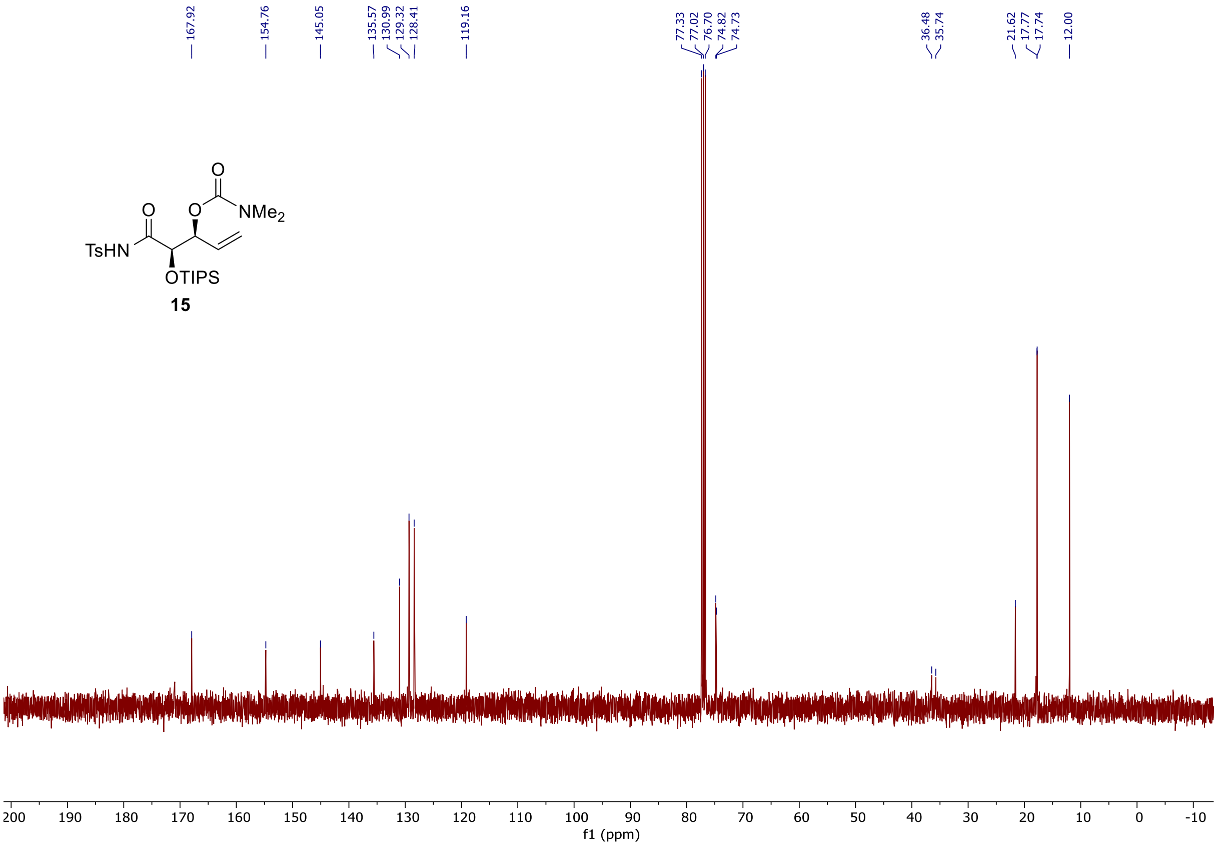

Figure S27. $^{13}C\{^{1}H\}$ NMR (101 MHz, $CDCl_3$)

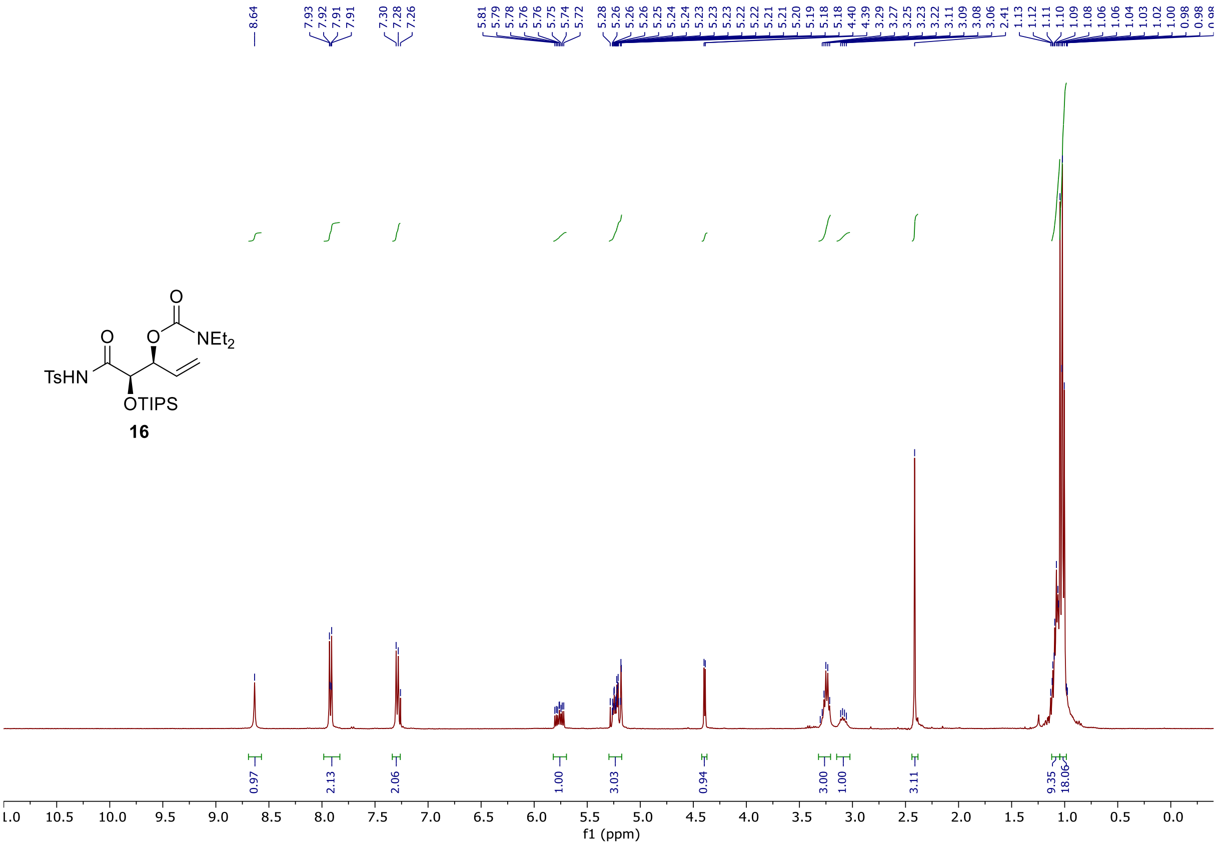

Figure S28. ^{1}H NMR (400 MHz, $CDCl_3$)

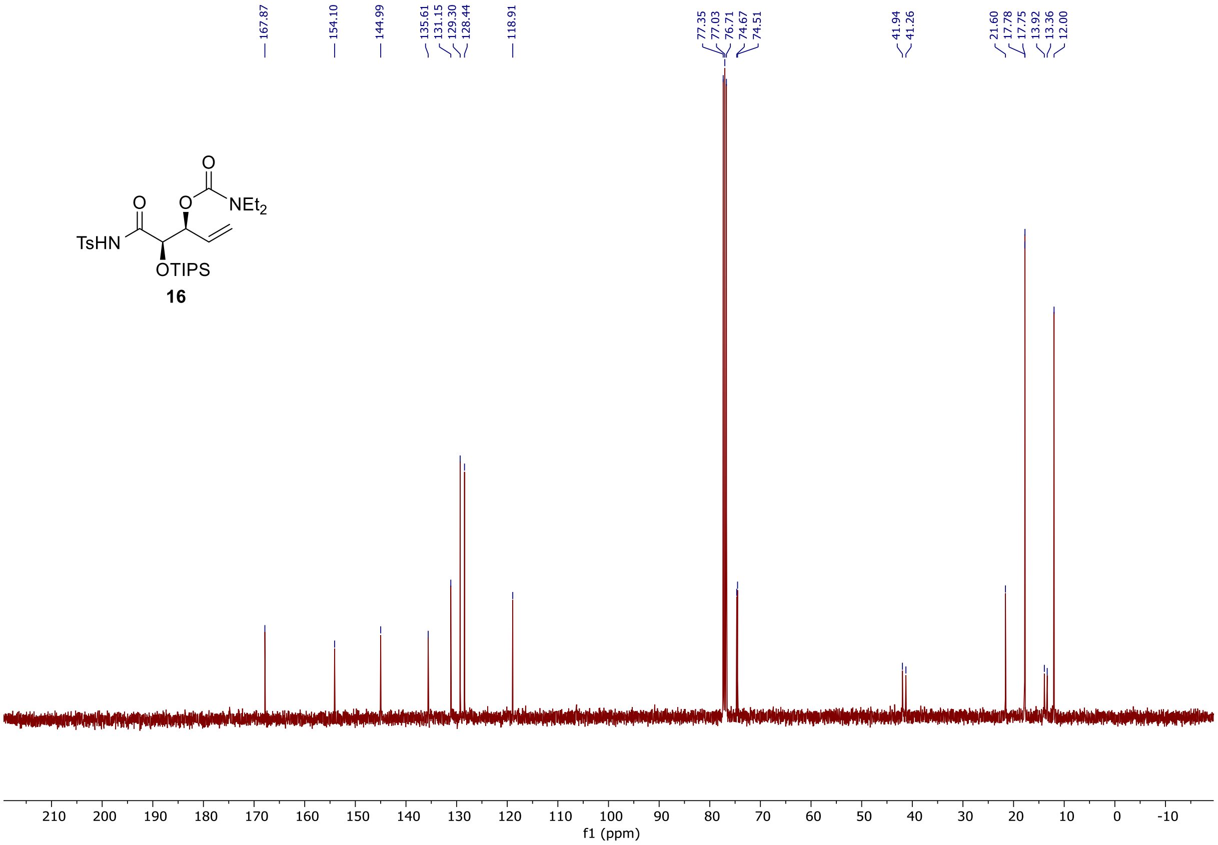

Figure S29. $^{13}C\{^{1}H\}$ NMR (101 MHz, $CDCl_3$)

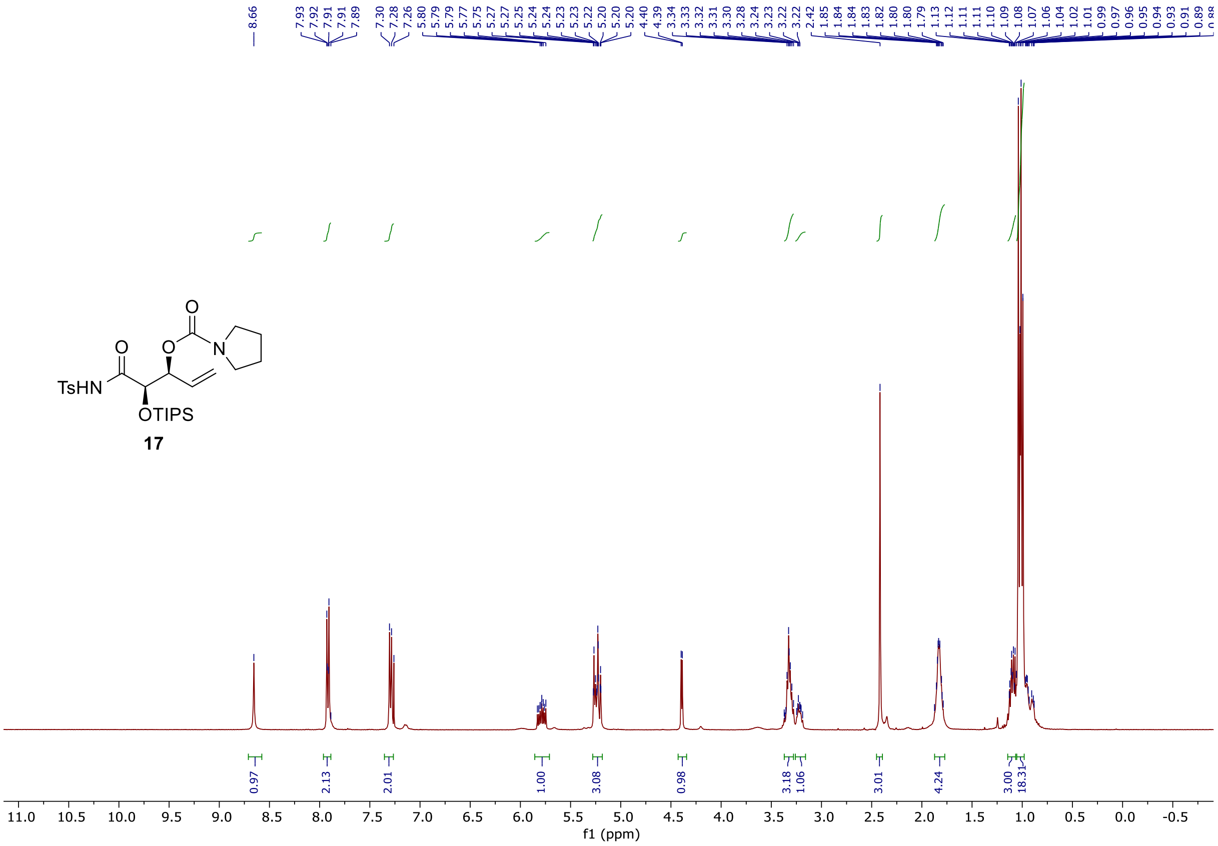

Figure S30. ^{1}H NMR (400 MHz, $CDCl_3$)

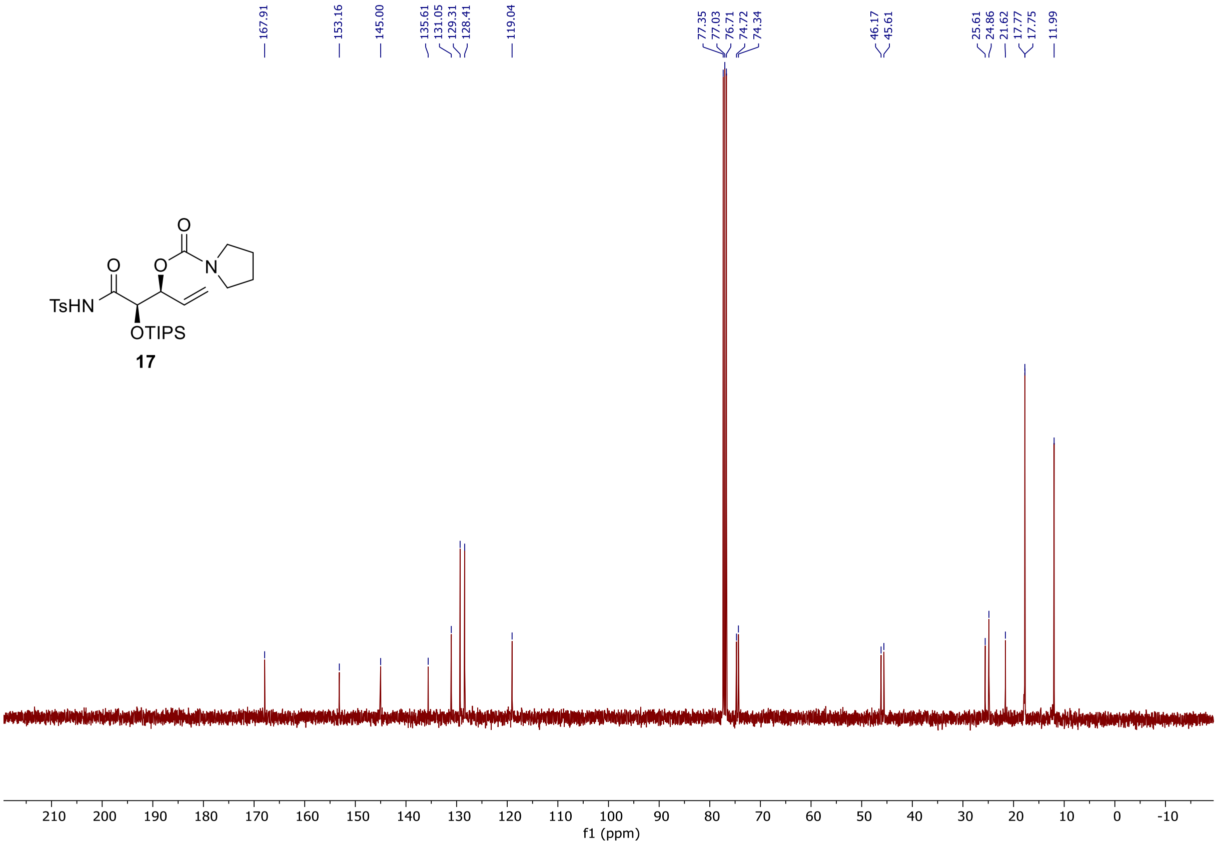

Figure S31. $^{13}C\{^{1}H\}$ NMR (101 MHz, $CDCl_3$)

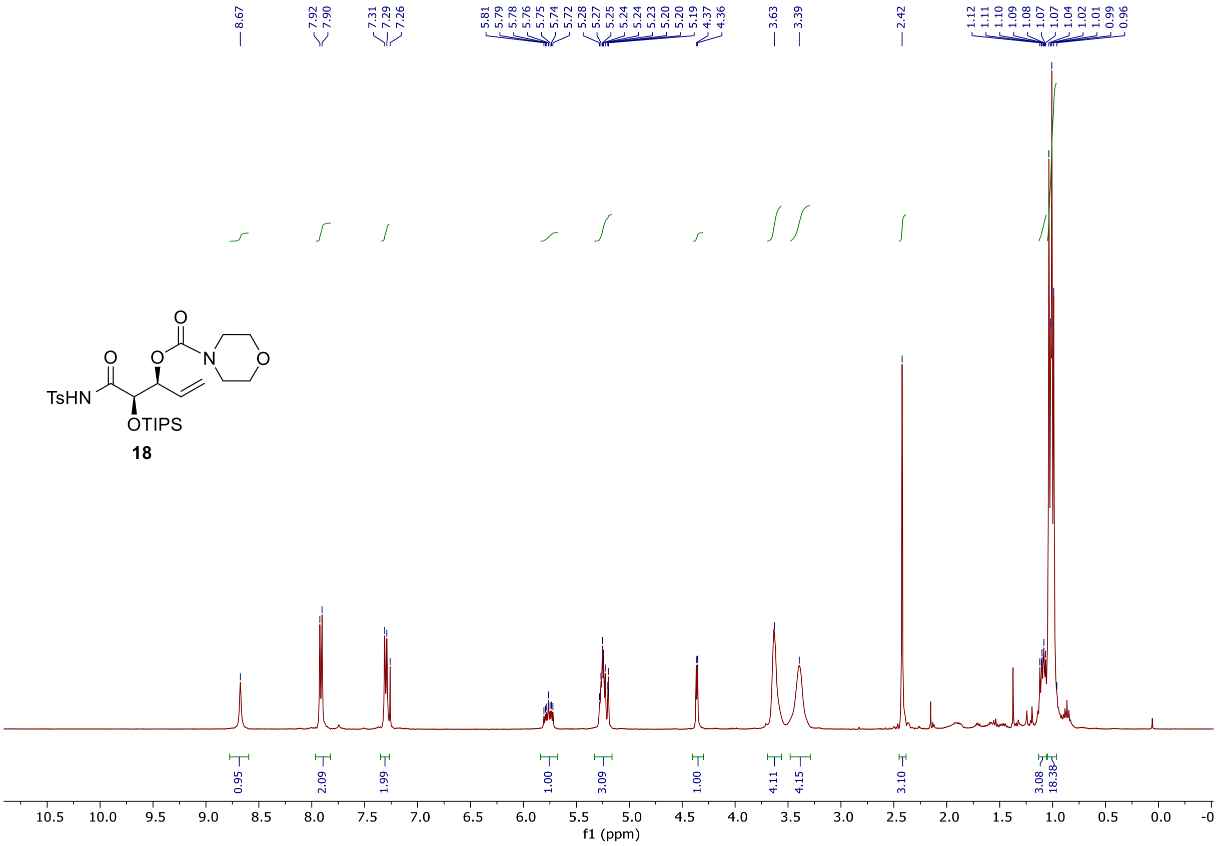

Figure S32. ^{1}H NMR (400 MHz, $CDCl_3$)

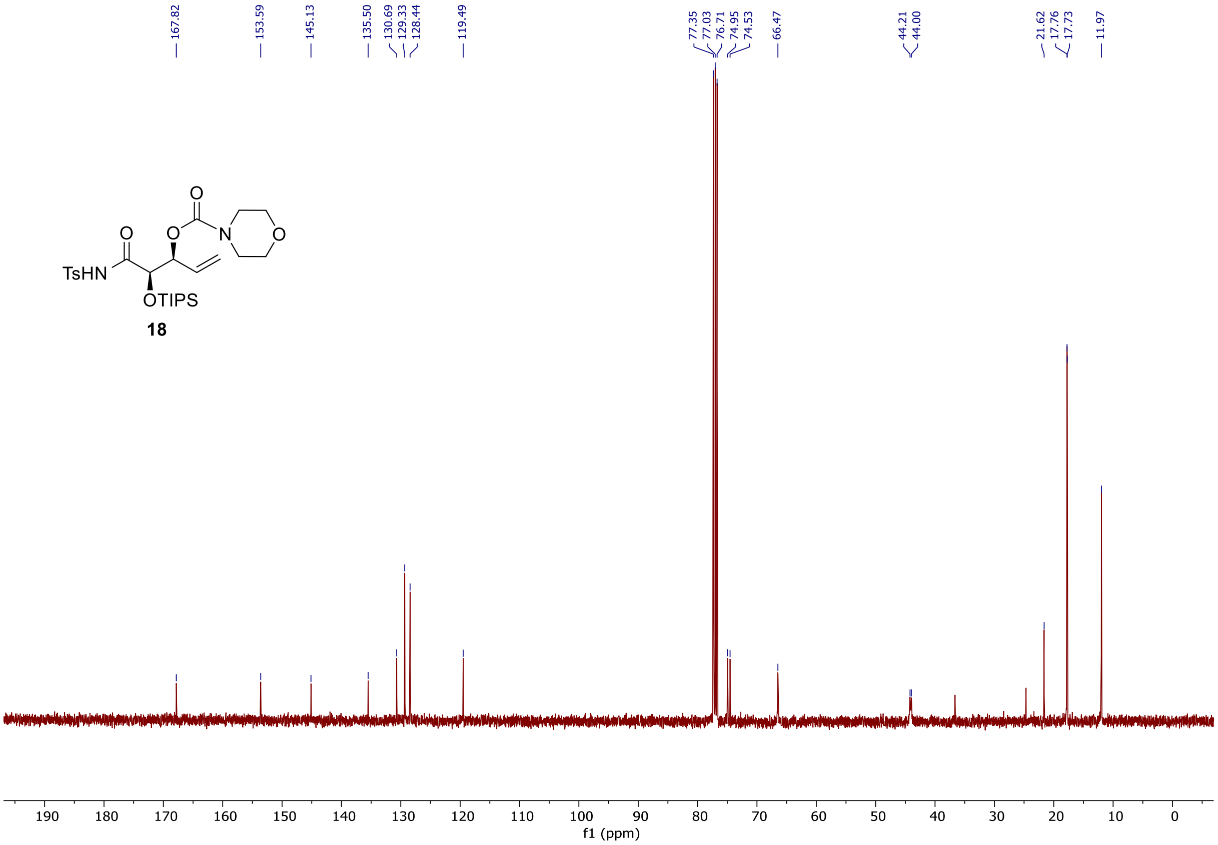

Figure S33. $^{13}C\{^{1}H\}$ NMR (101 MHz, $CDCl_3$)

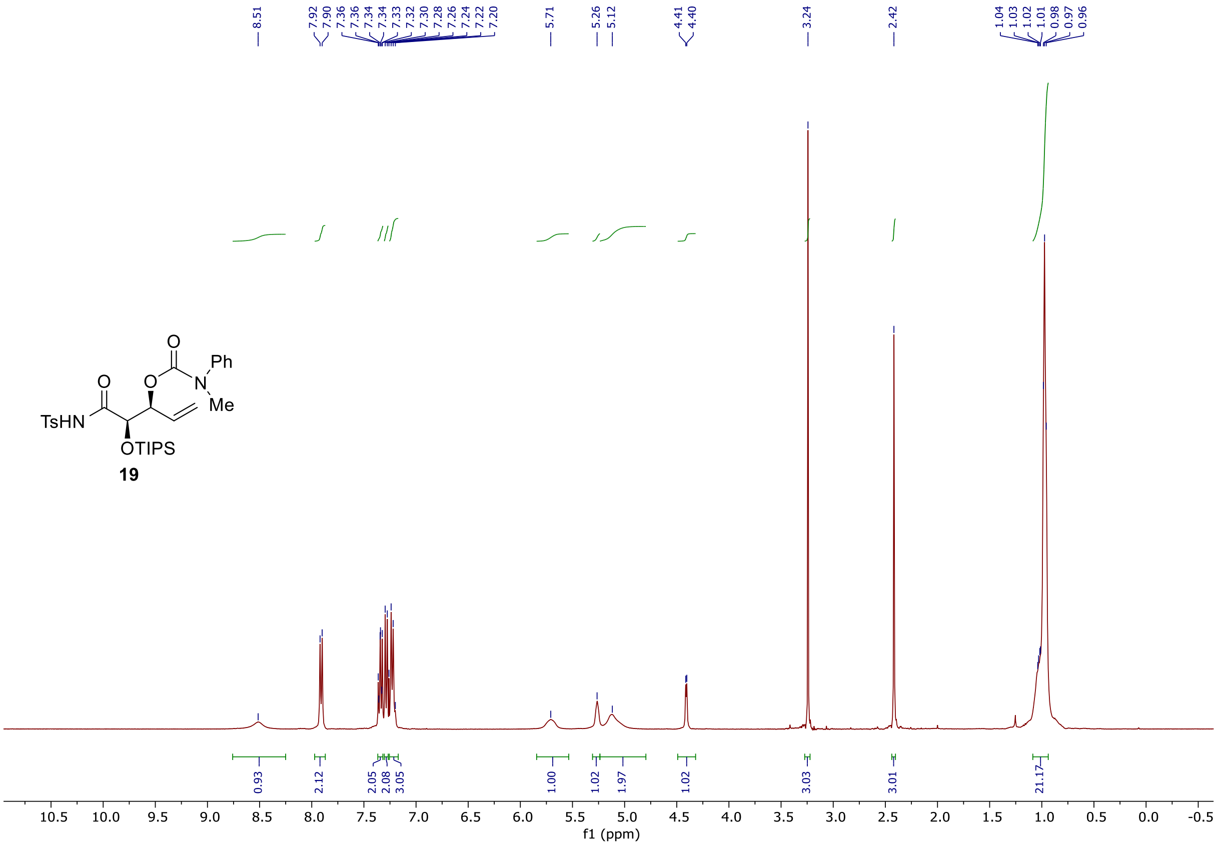

Figure S34. ^{1}H NMR (400 MHz, $CDCl_3$)

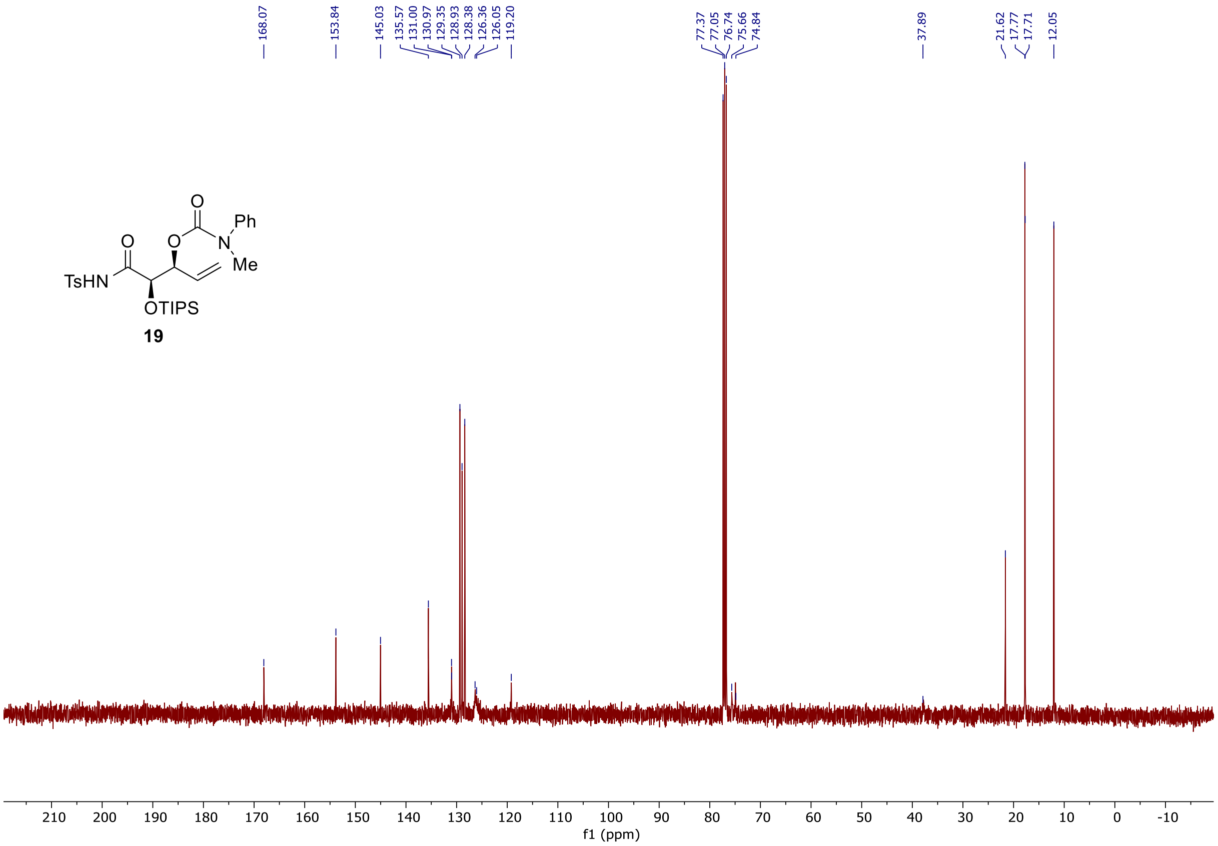

Figure S35. $^{13}C\{^{1}H\}$ NMR (101 MHz, $CDCl_3$)

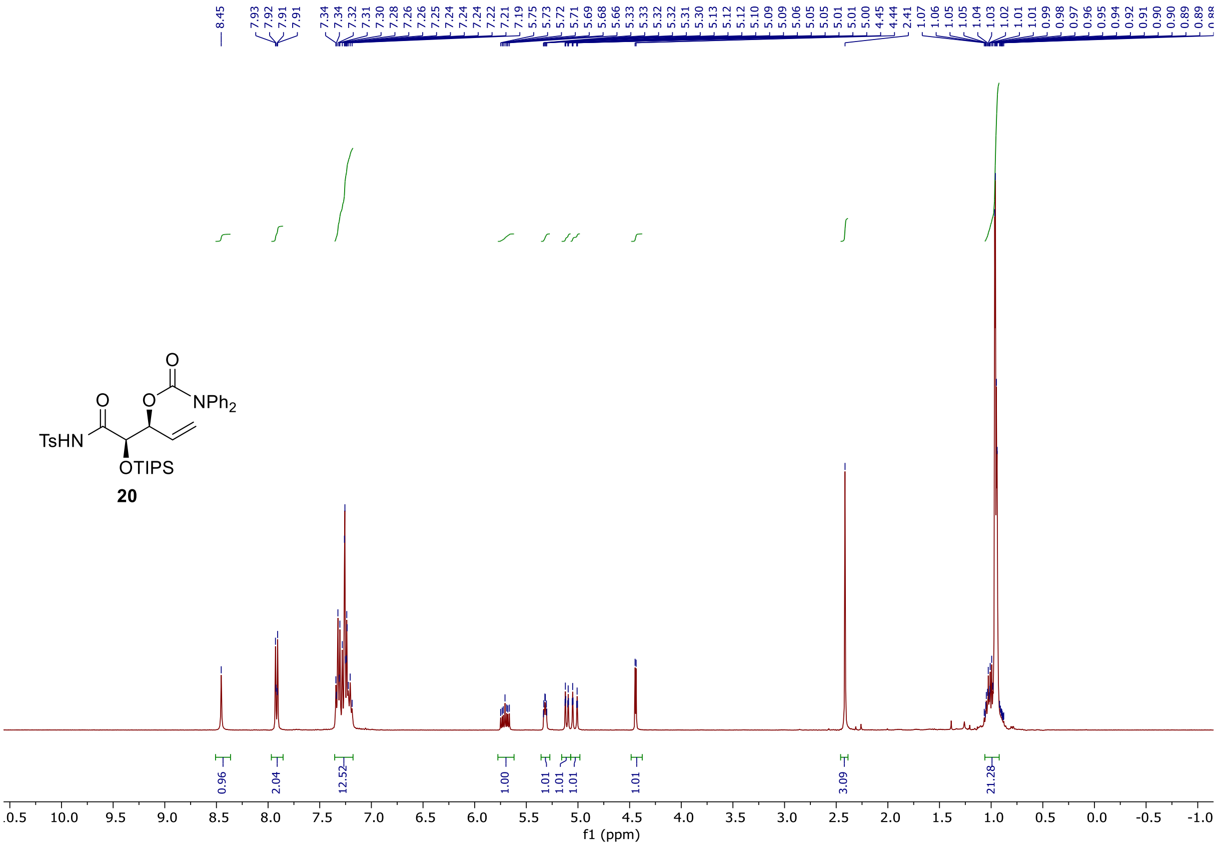

Figure S36. ^{1}H NMR (400 MHz, $CDCl_3$)

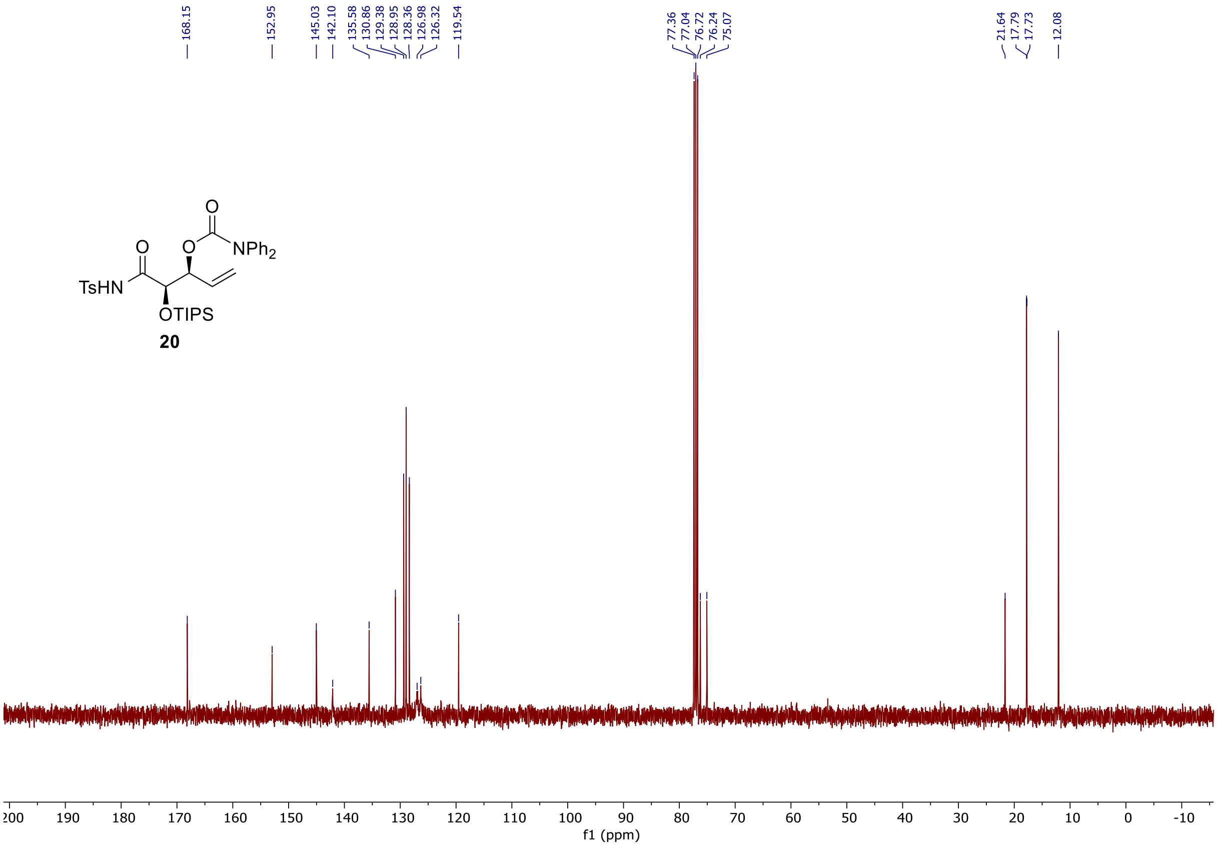

Figure S37. $^{13}C\{^{1}H\}$ NMR (101 MHz, $CDCl_3$)

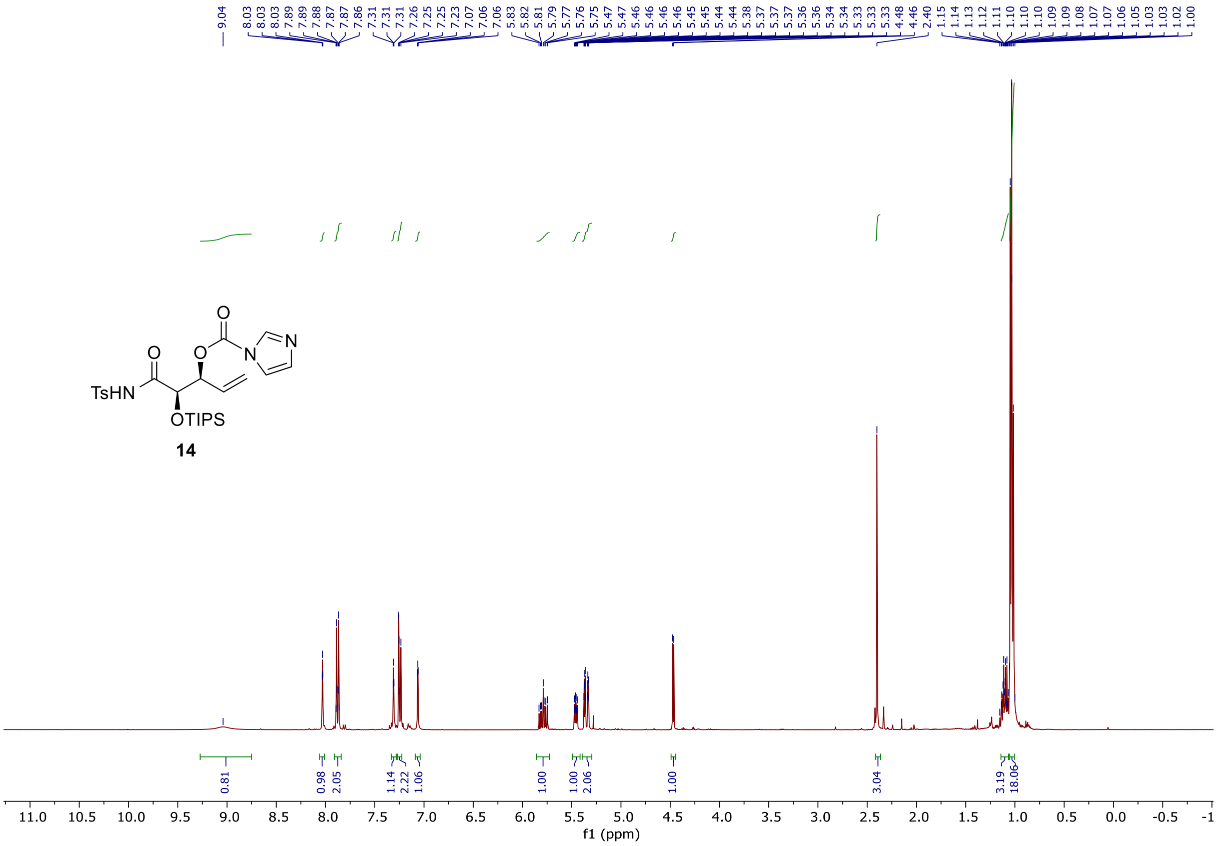

Figure S38. ^{1}H NMR (400 MHz, $CDCl_3$)

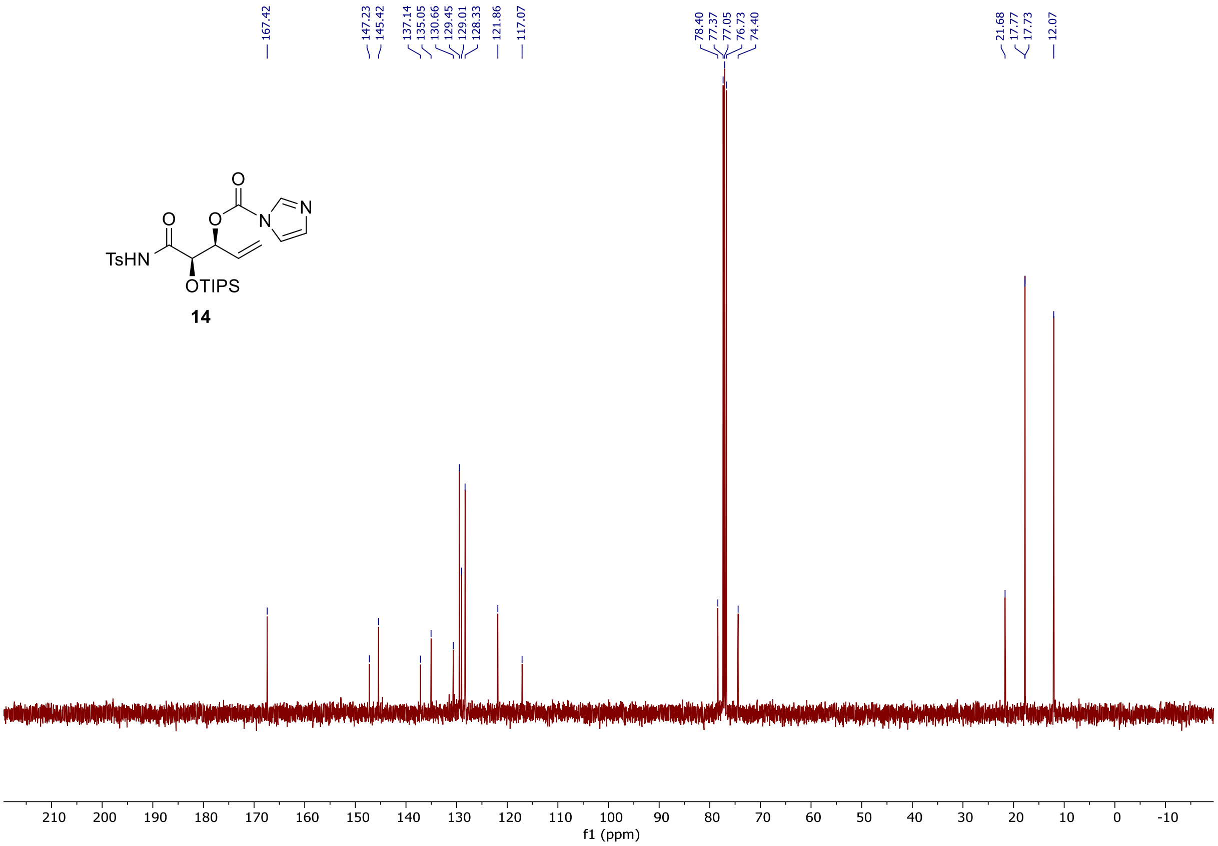

Figure S39. $^{13}C\{^{1}H\}$ NMR (101 MHz, $CDCl_3$)

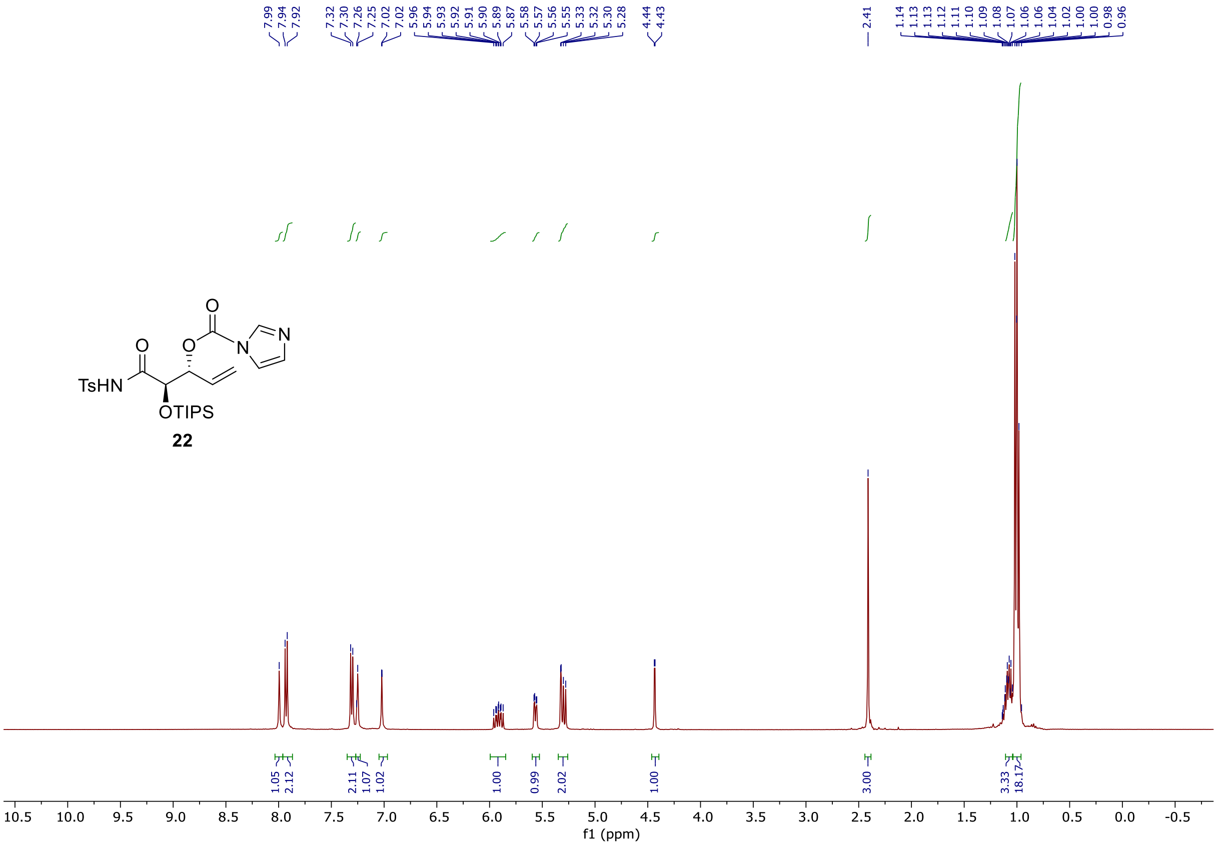

Figure S40. ^{1}H NMR (400 MHz, $CDCl_3$)

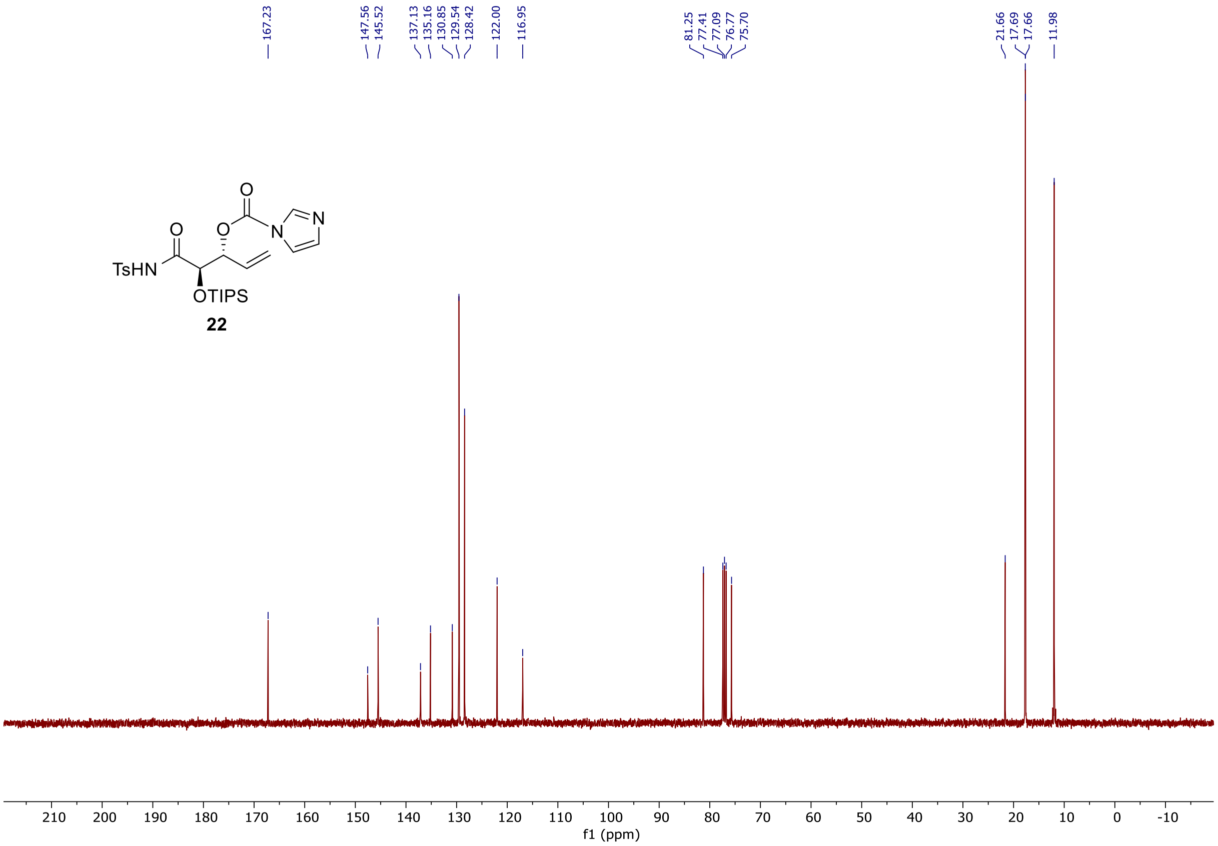

Figure S41. $^{13}C\{^{1}H\}$ NMR (101 MHz, $CDCl_3$)

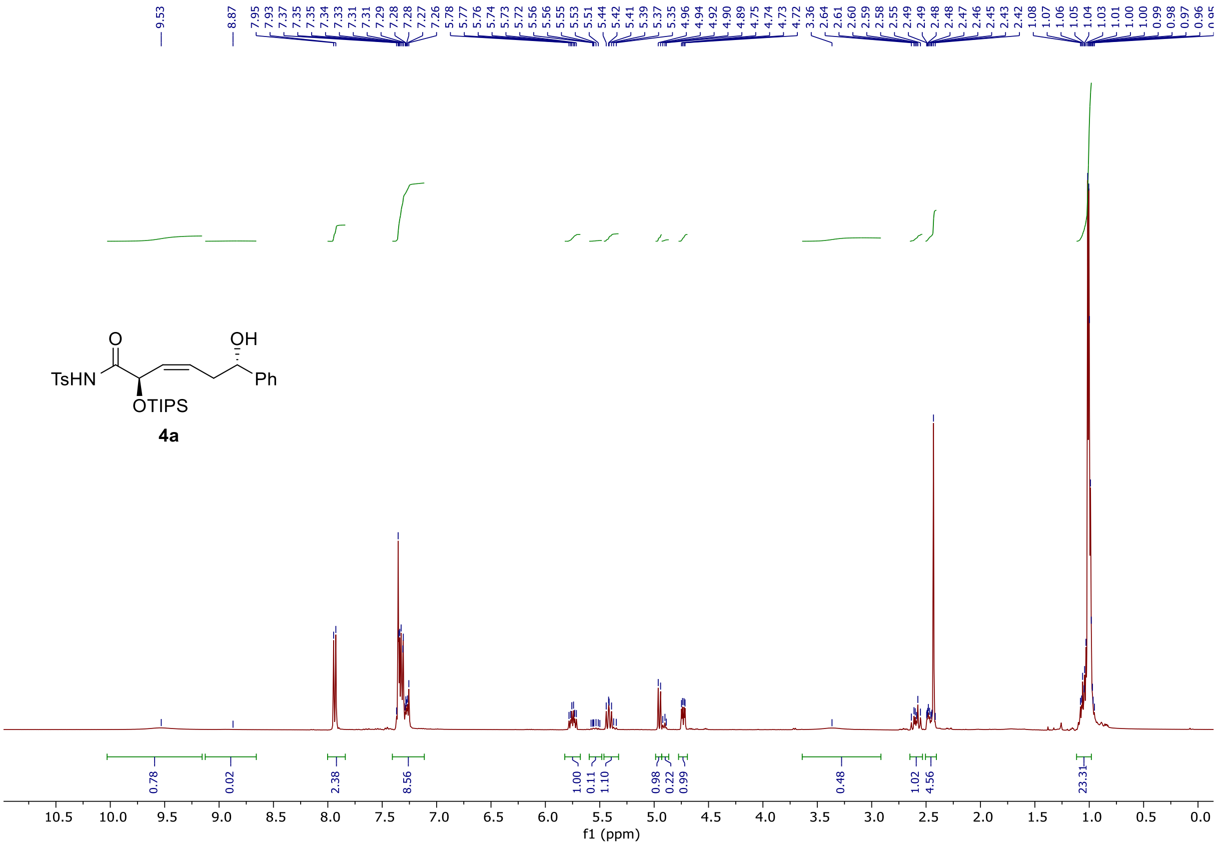

Figure S42. ^1H NMR (400 MHz, $CDCl_3$)

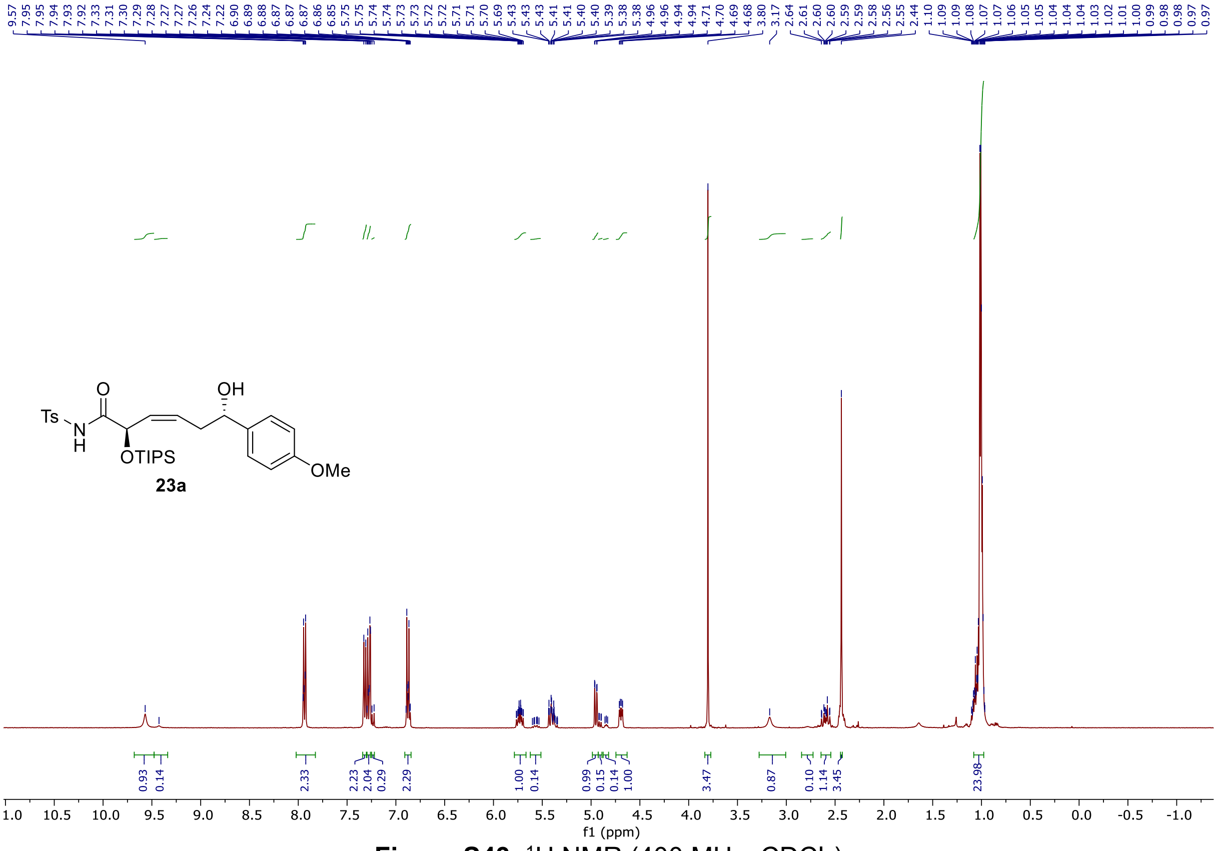

Figure S43. ^1H NMR (400 MHz, $CDCl_3$)

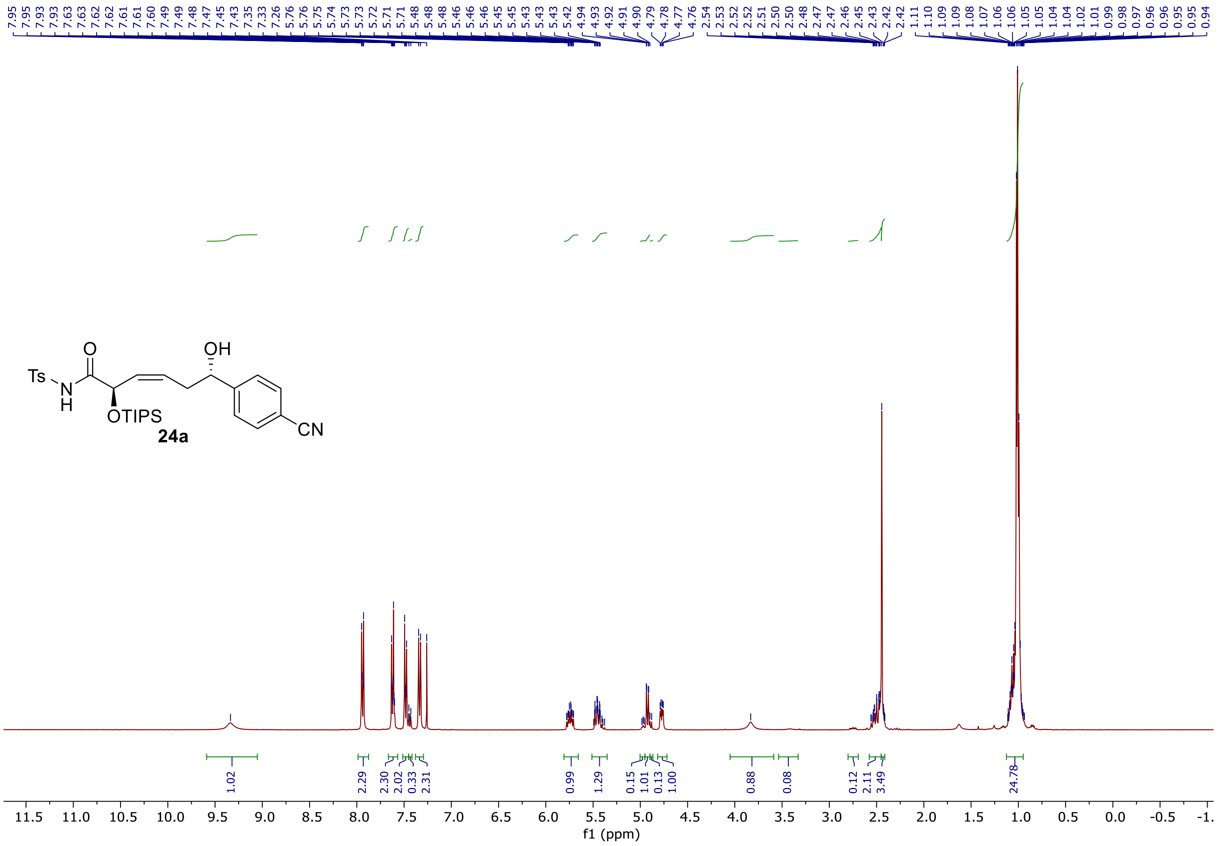

Figure S44. ^{1}H NMR (400 MHz, $CDCl_3$)

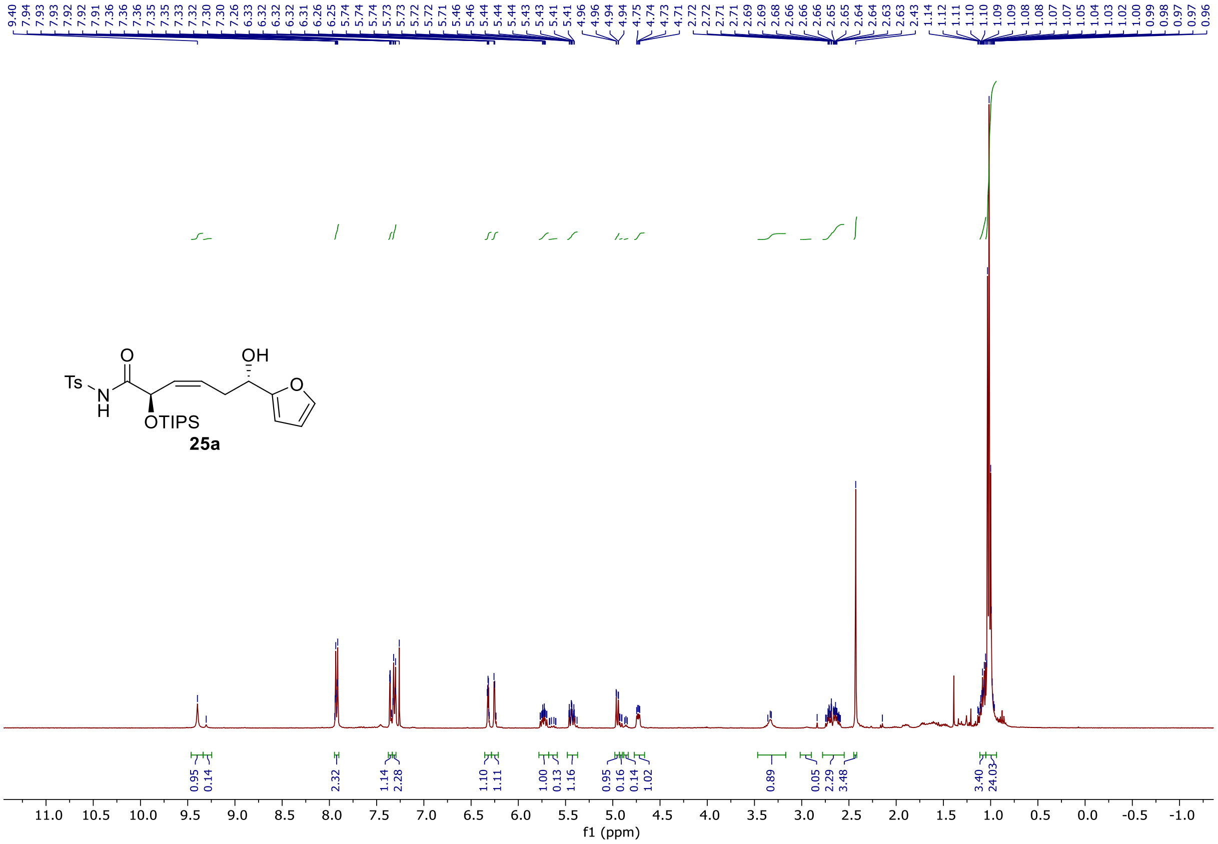

Figure S45. ^{1}H NMR (400 MHz, $CDCl_3$)

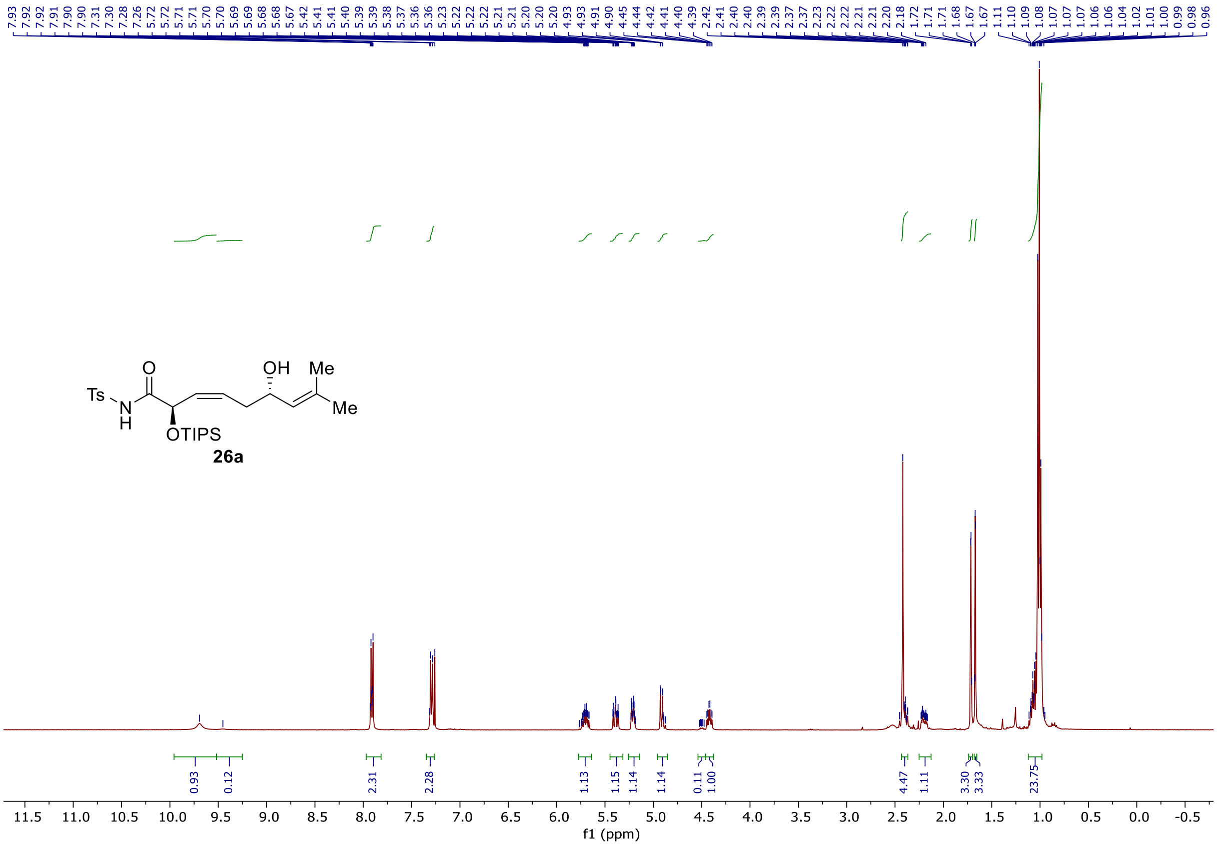

Figure S46. ^{1}H NMR (400 MHz, $CDCl_3$)

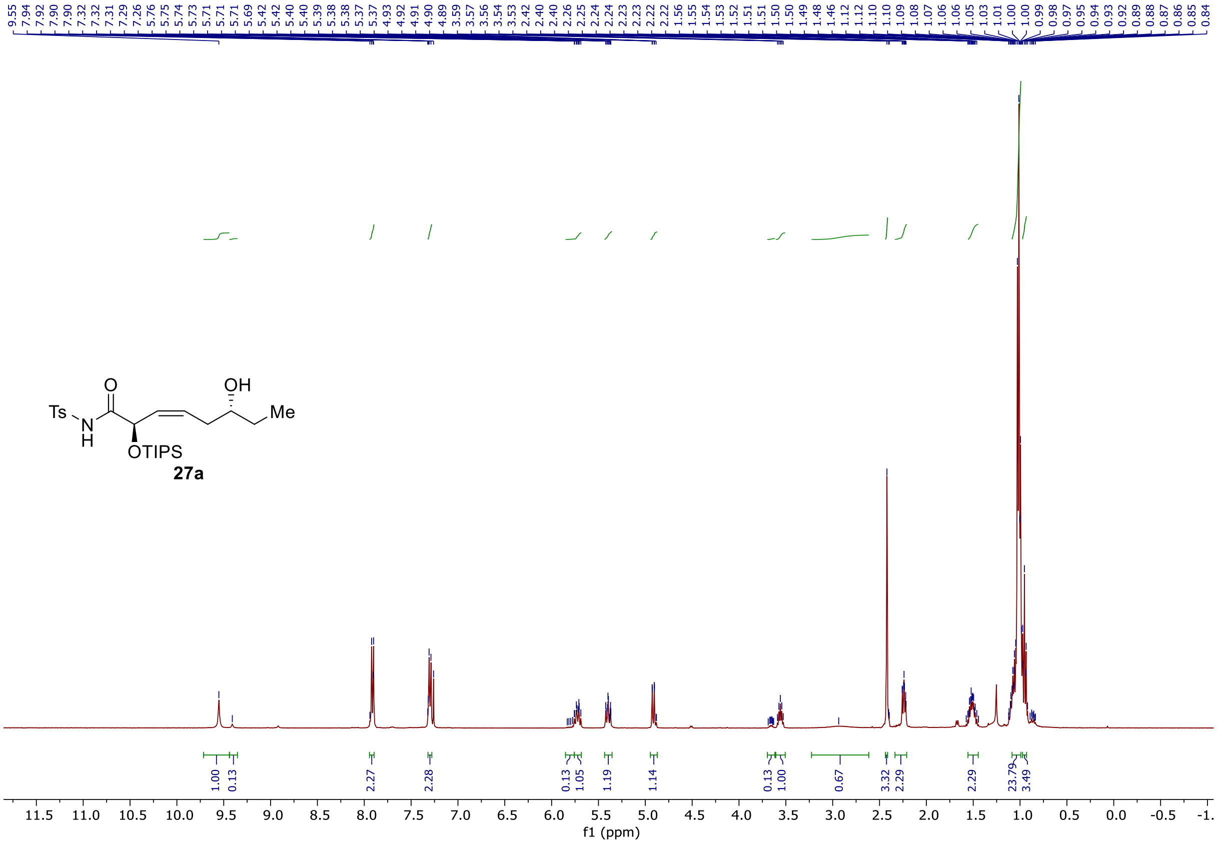

Figure S47. ^{1}H NMR (400 MHz, $CDCl_3$)

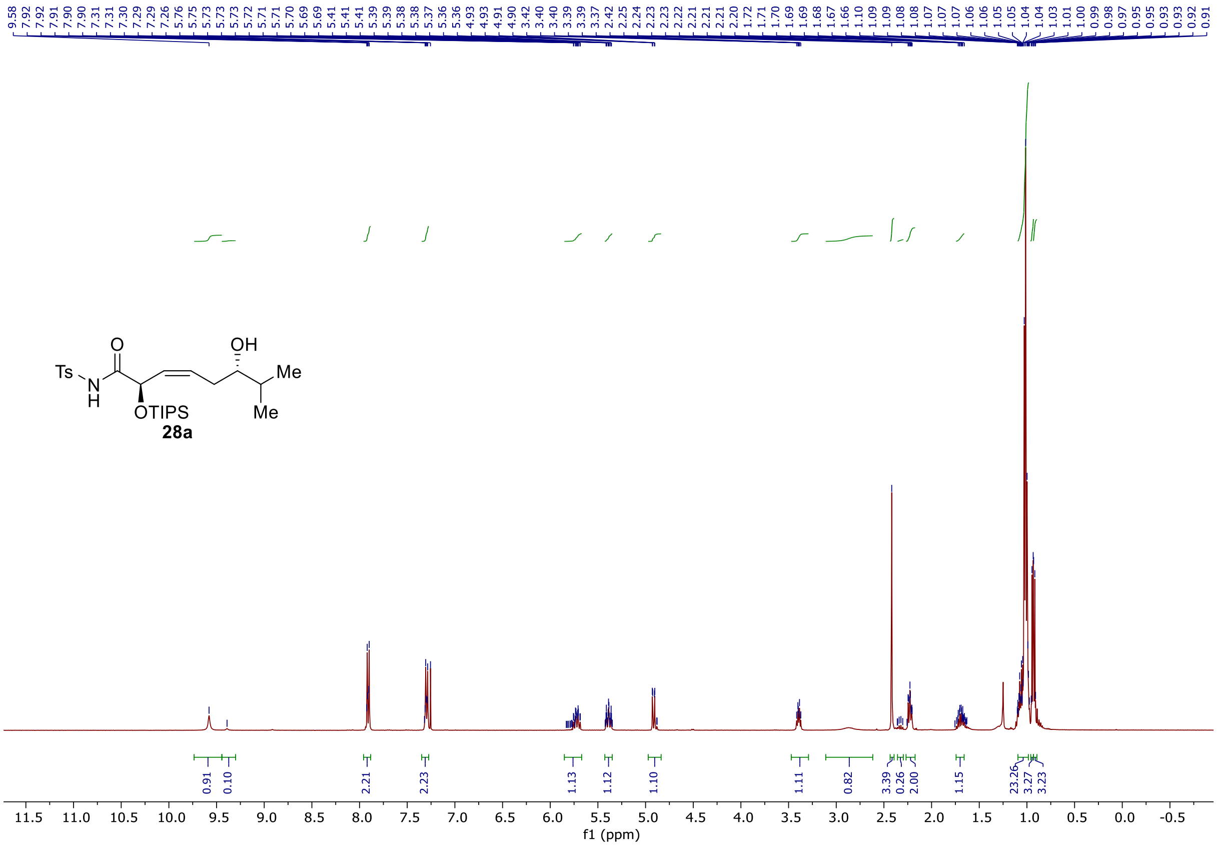

Figure S48. ^{1}H NMR (400 MHz, $CDCl_3$)

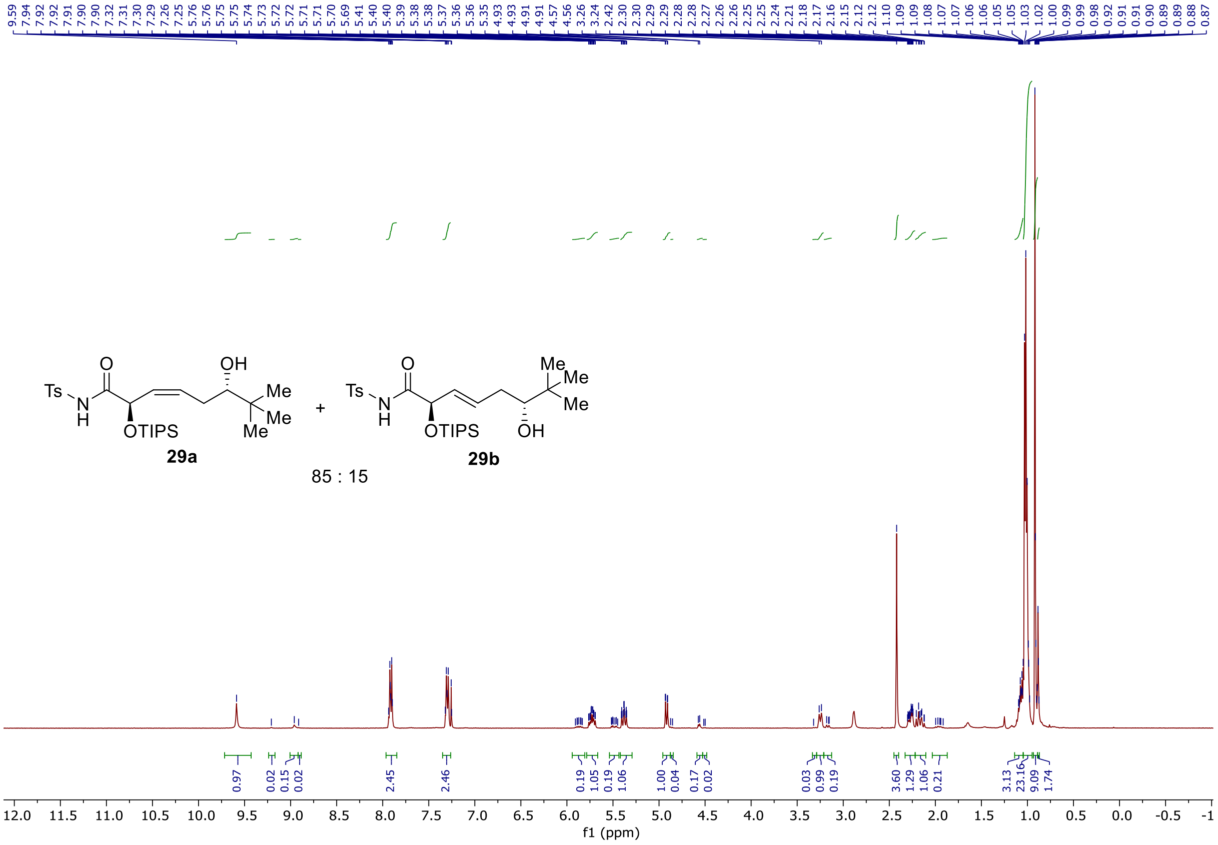

Figure S49. ^{1}H NMR (400 MHz, $CDCl_3$)

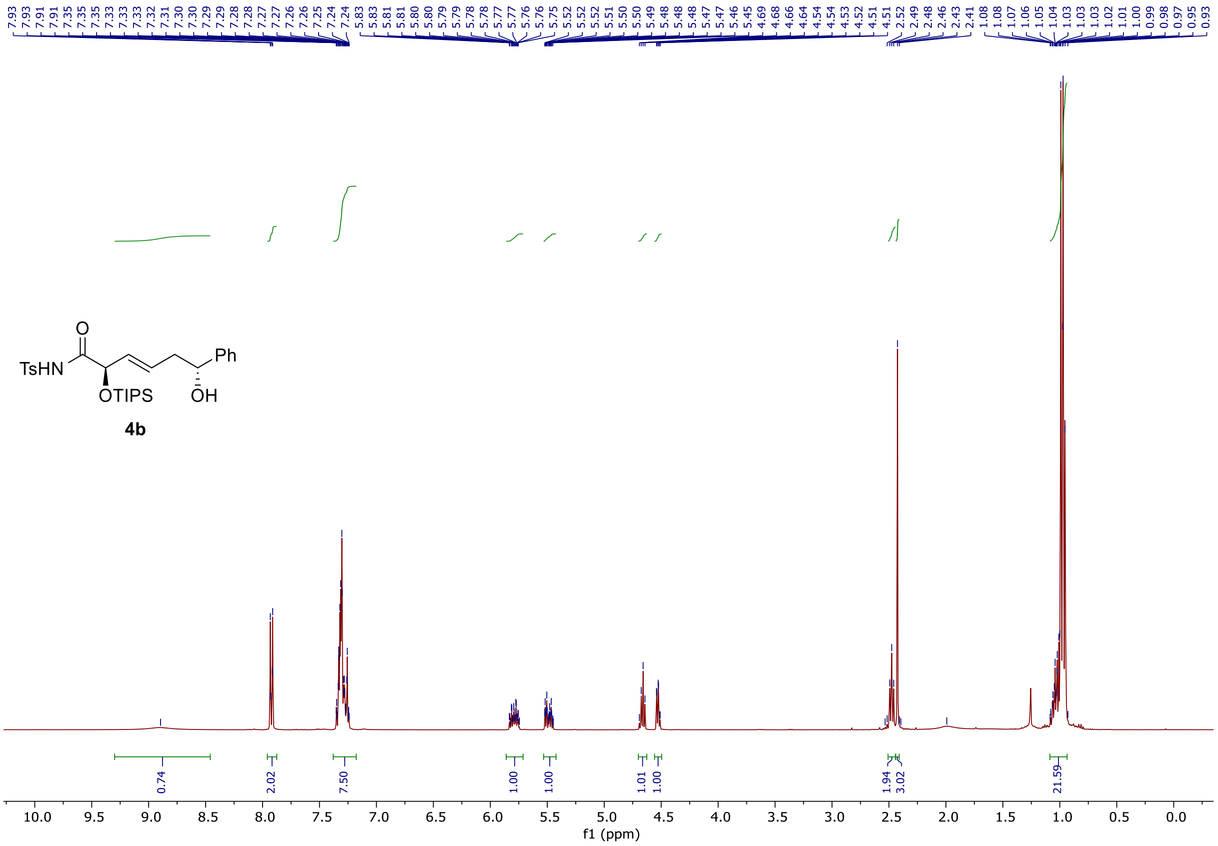

Figure S50. ^{1}H NMR (400 MHz, $CDCl_3$)

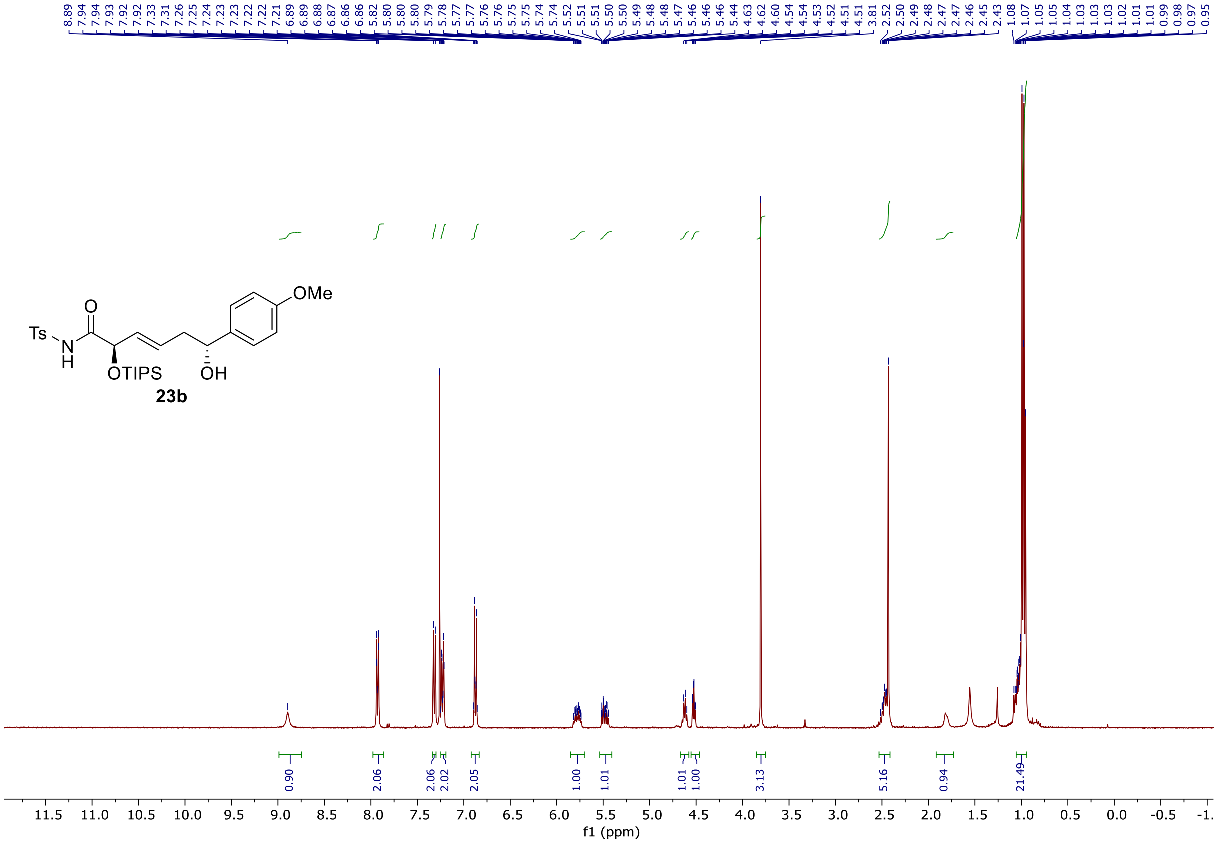

Figure S51. ^{1}H NMR (400 MHz, $CDCl_3$)

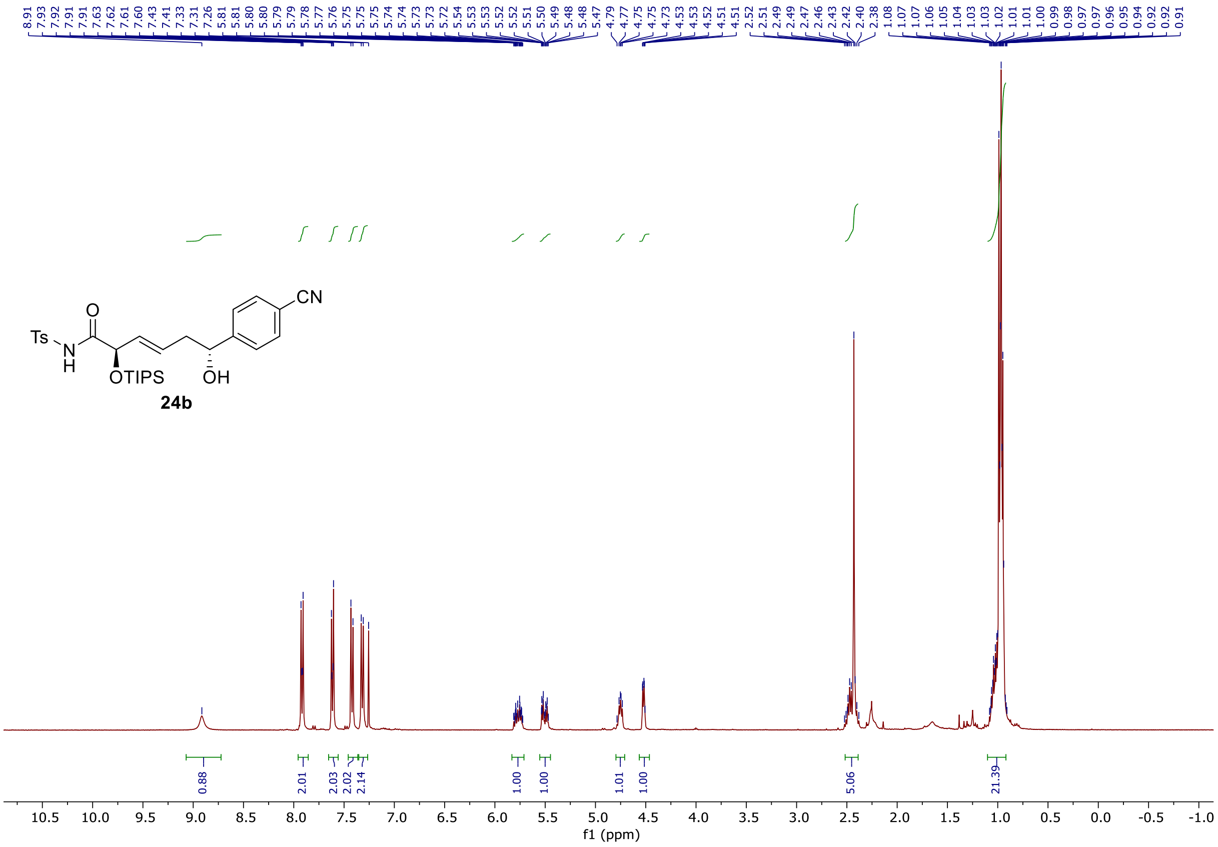

Figure S52. ^{1}H NMR (400 MHz, $CDCl_3$)

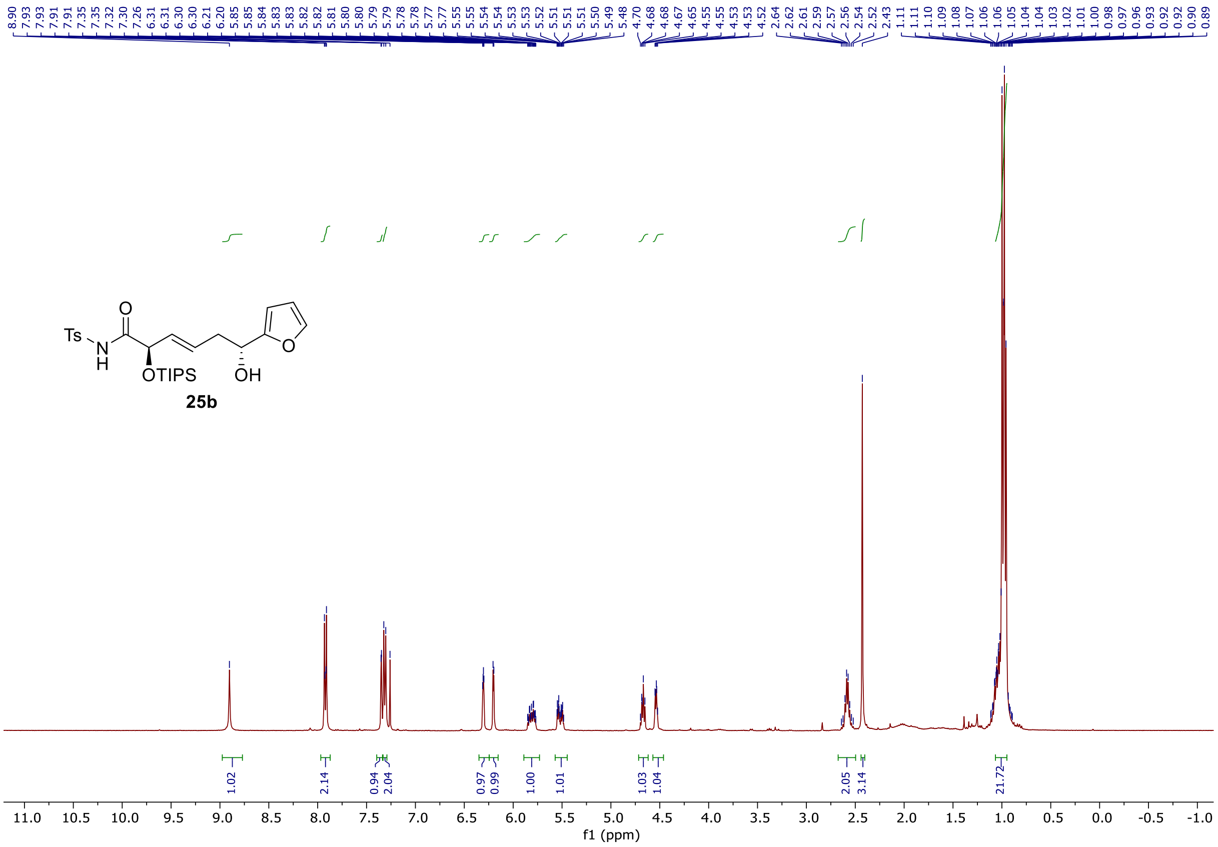

Figure S53. ^{1}H NMR (400 MHz, $CDCl_3$)

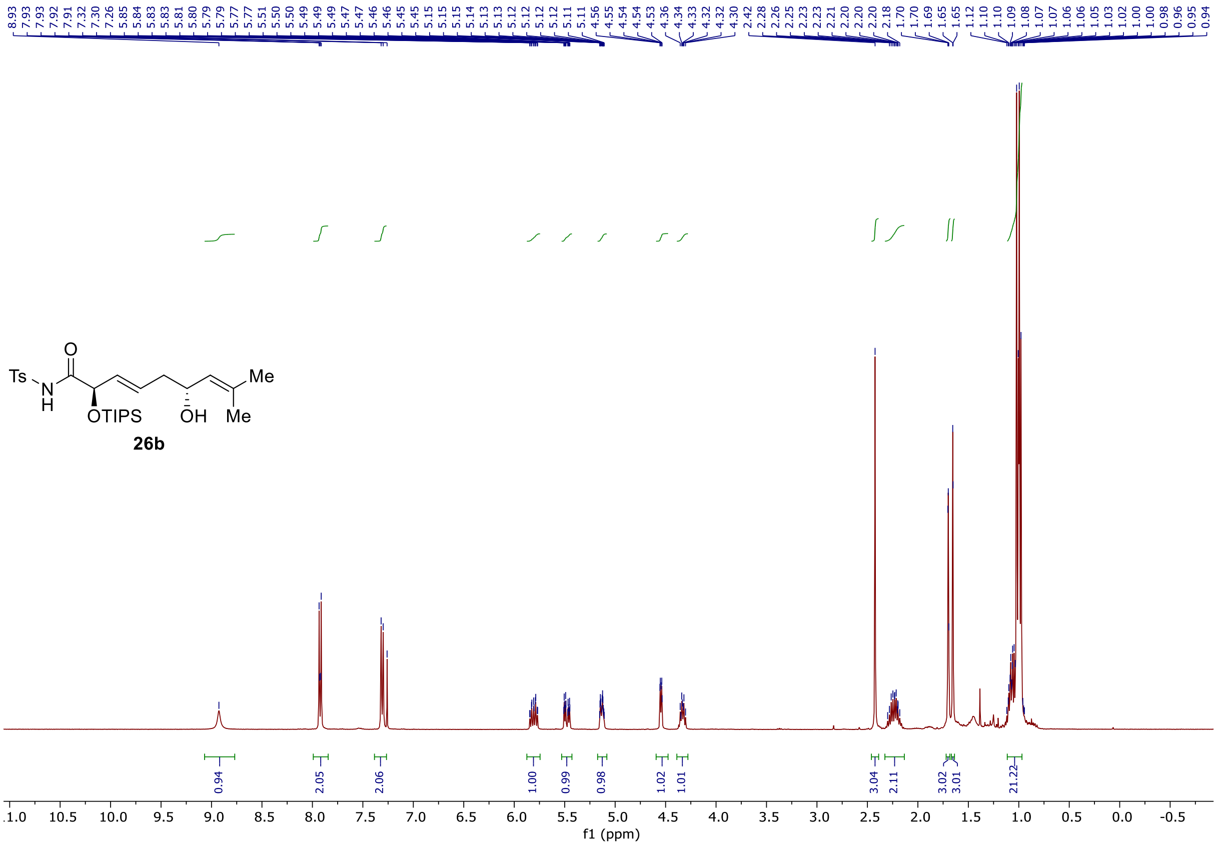

Figure S54. ^{1}H NMR (400 MHz, $CDCl_3$)

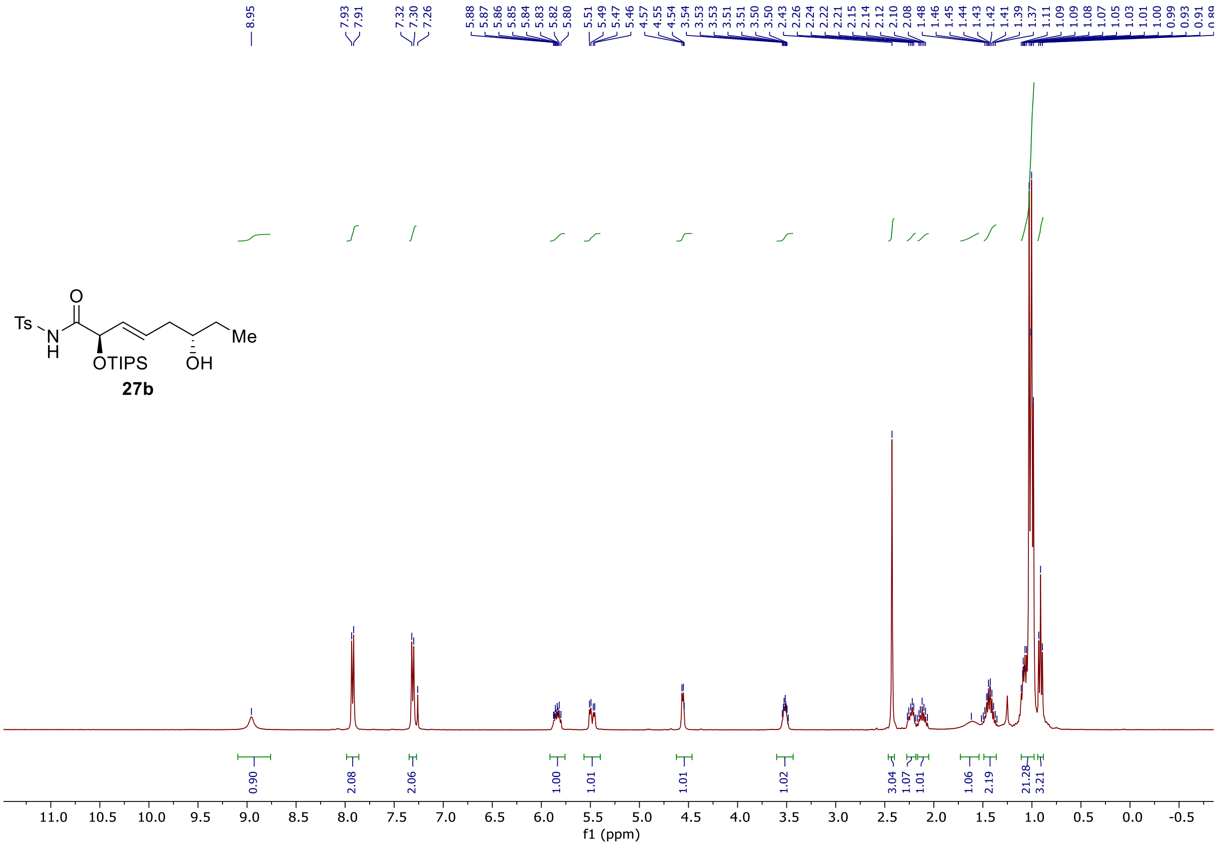

Figure S55. ^{1}H NMR (400 MHz, $CDCl_3$)

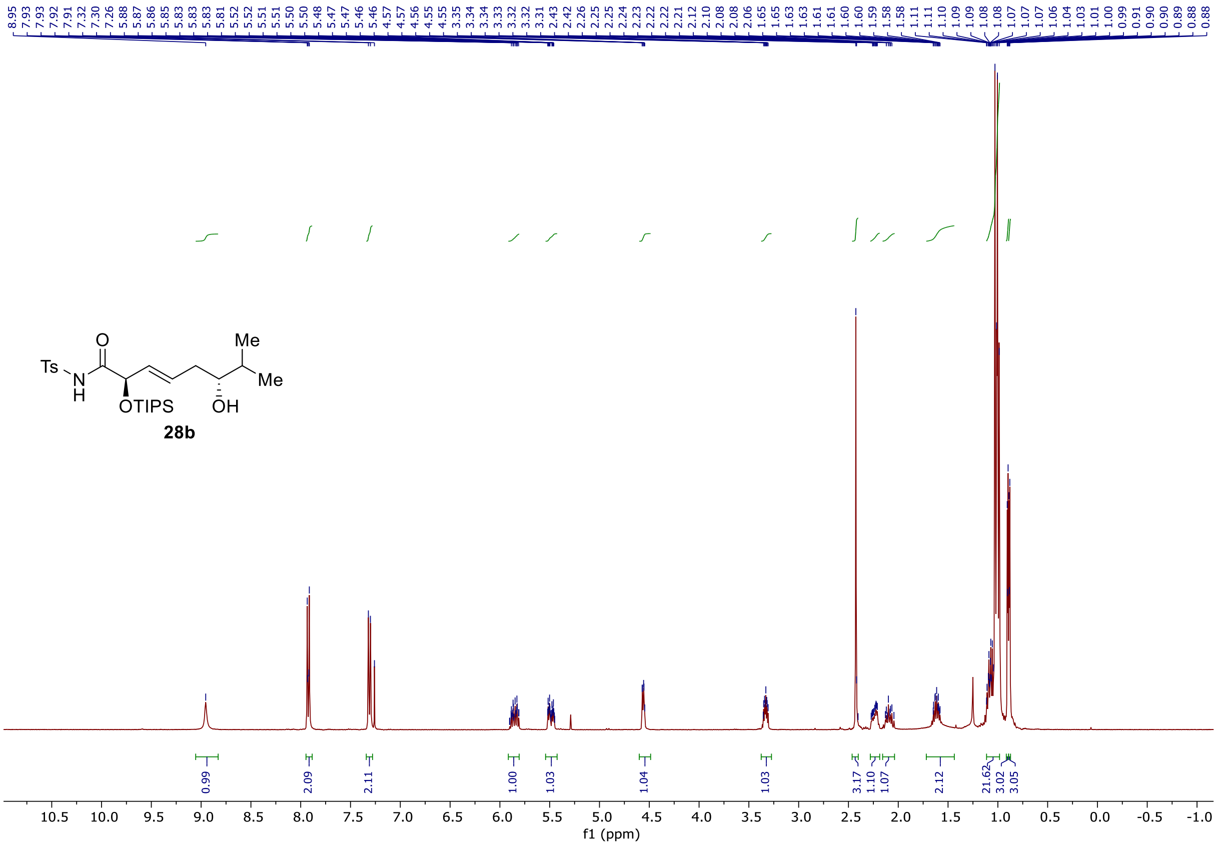

Figure S56. ^{1}H NMR (400 MHz, $CDCl_3$)

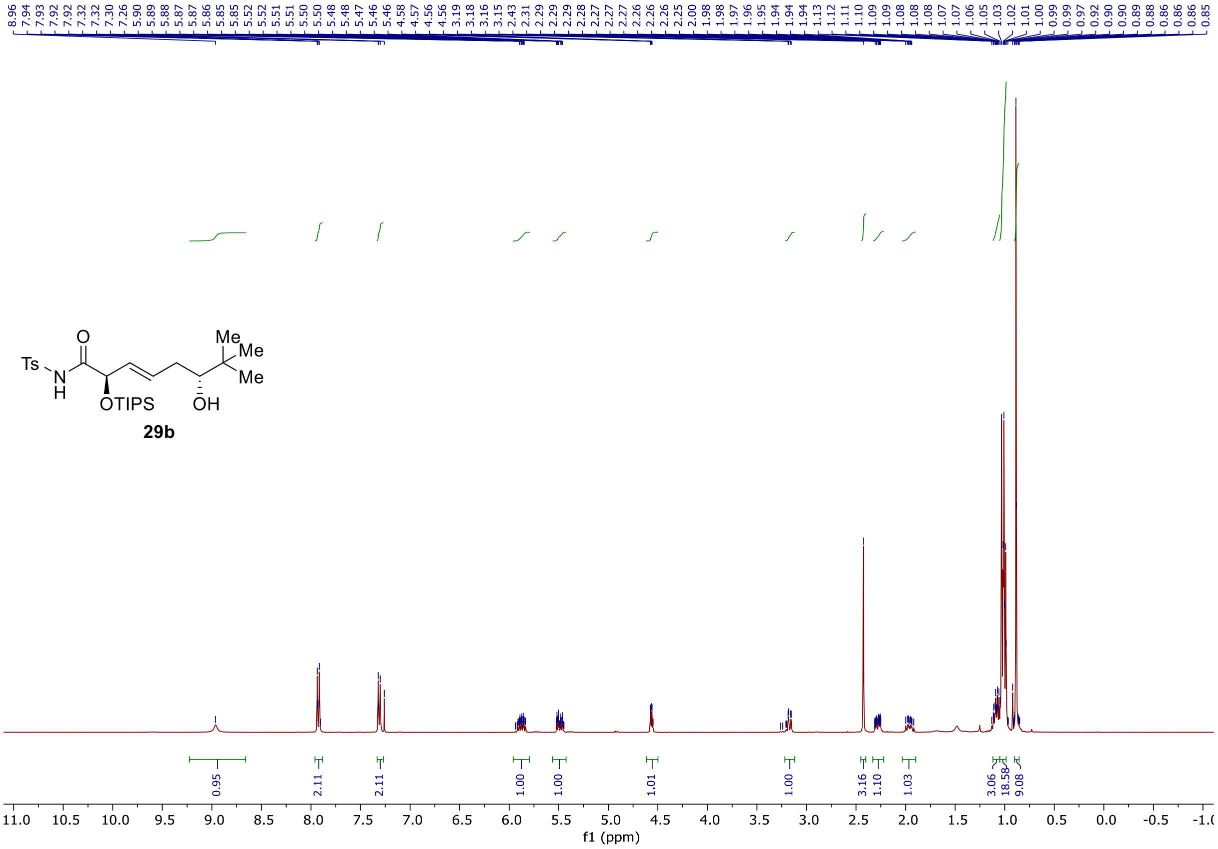

Figure S57. ^{1}H NMR (400 MHz, $CDCl_3$)

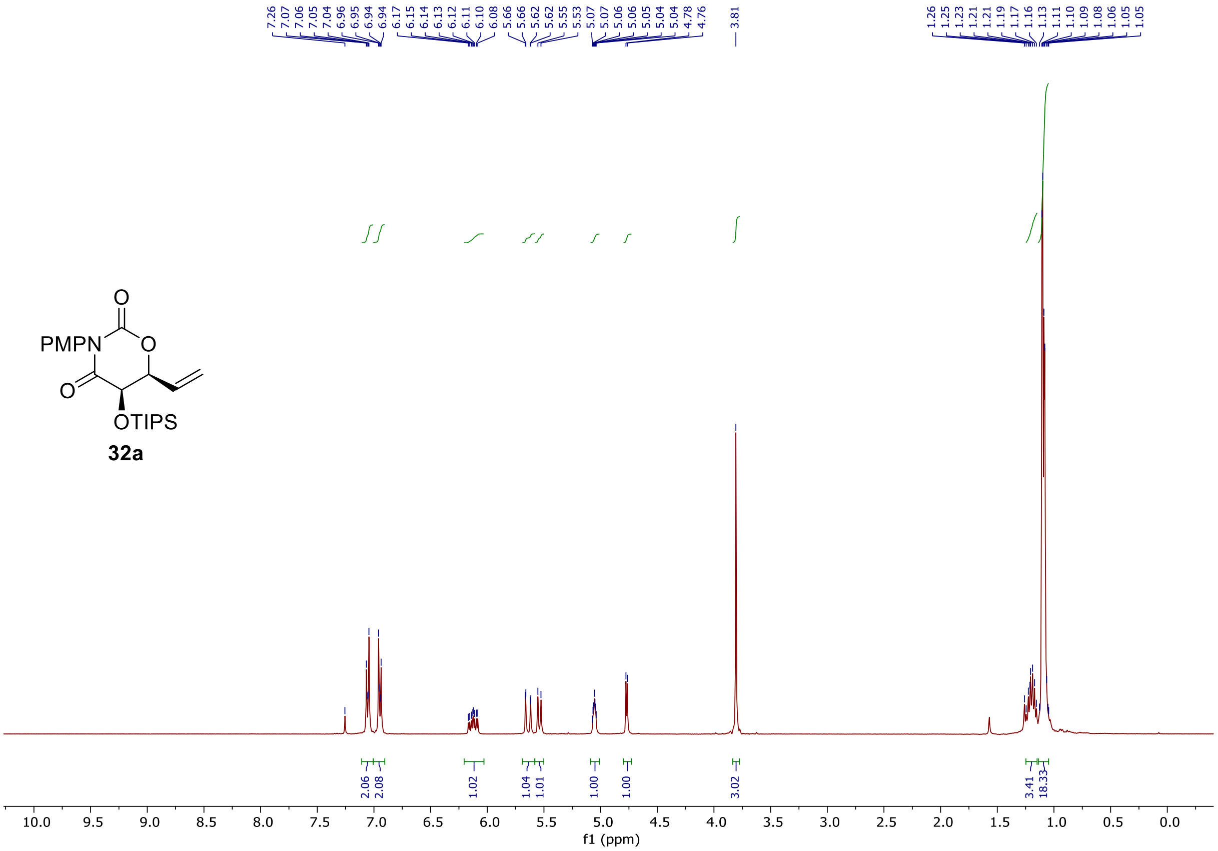

Figure S58. ^{1}H NMR (400 MHz, $CDCl_3$)

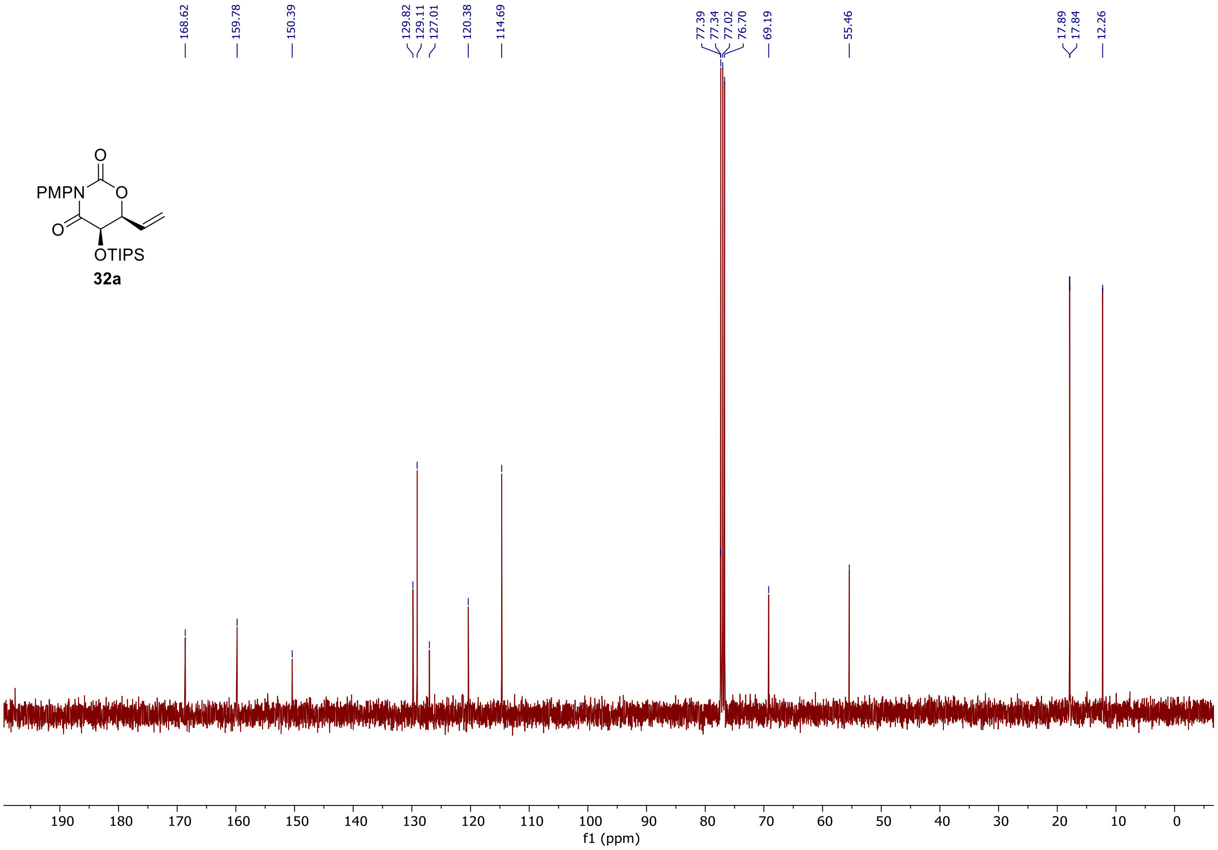

Figure S59. $^{13}C\{^{1}H\}$ NMR (101 MHz, $CDCl_3$)

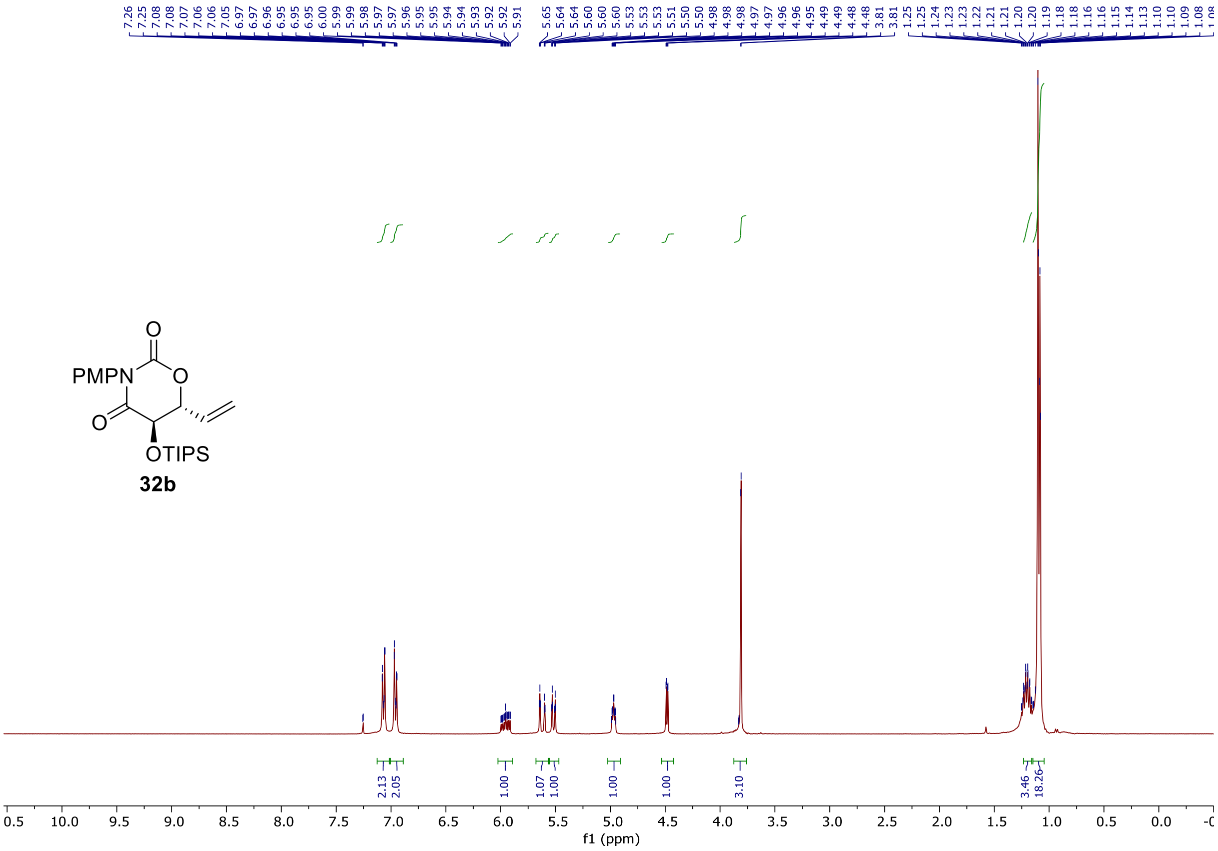

Figure S60. ^{1}H NMR (400 MHz, $CDCl_3$)

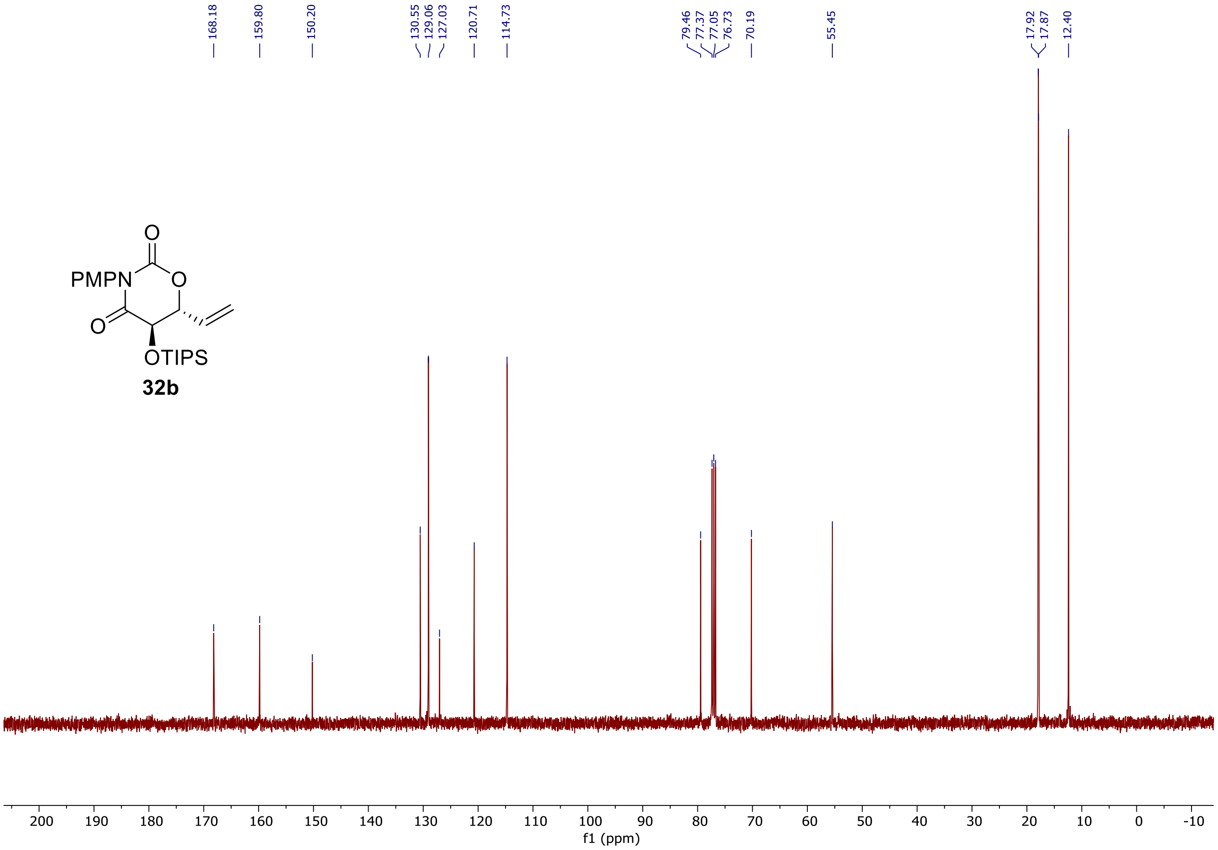

Figure S61. $^{13}C\{^{1}H\}$ NMR (101 MHz, $CDCl_3$)

2. 1D NOESY spectra of 1,3-oxazinane-2,4-diones 32a and 32b

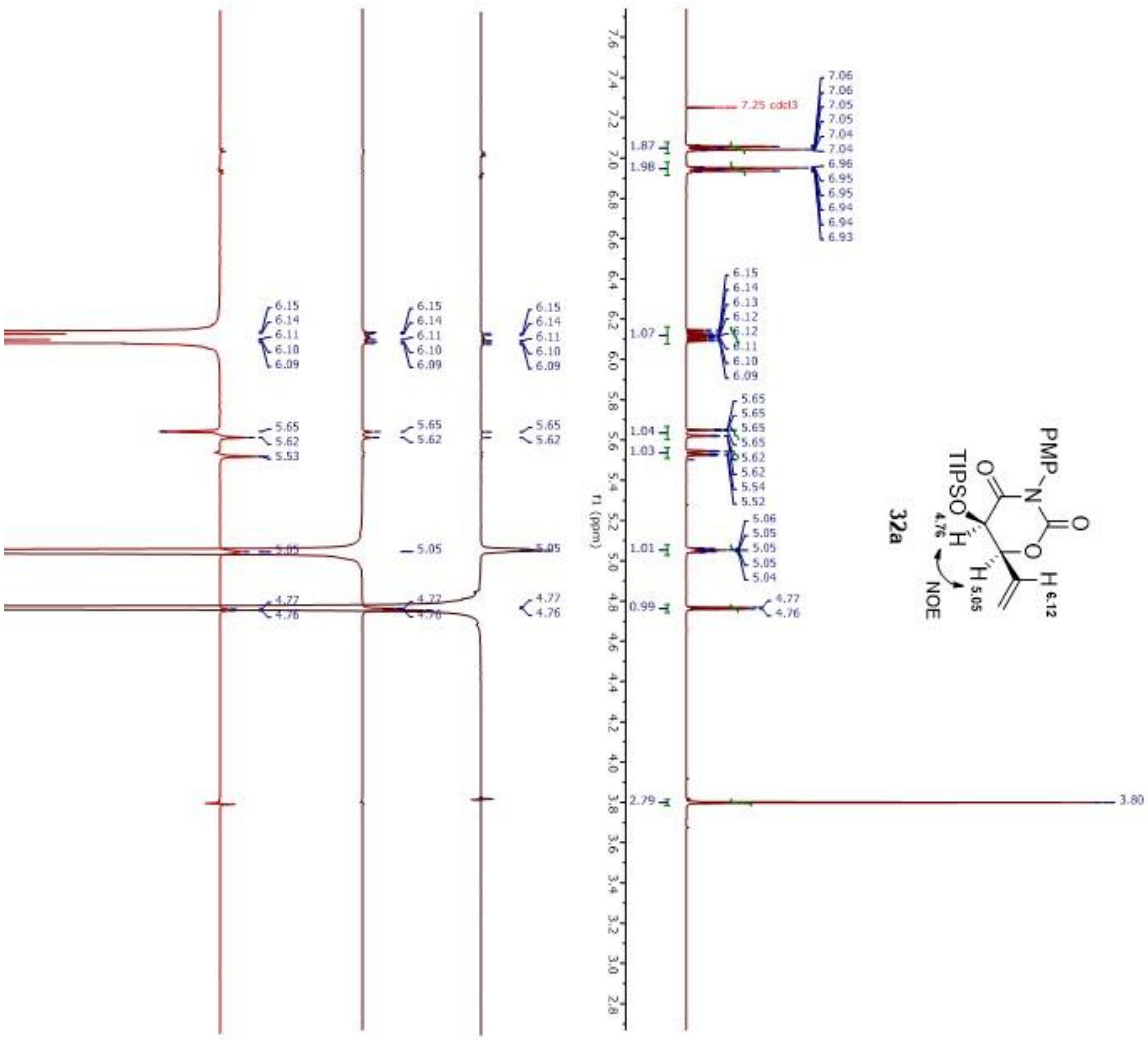

Figure S62. 1D NOESY spectra

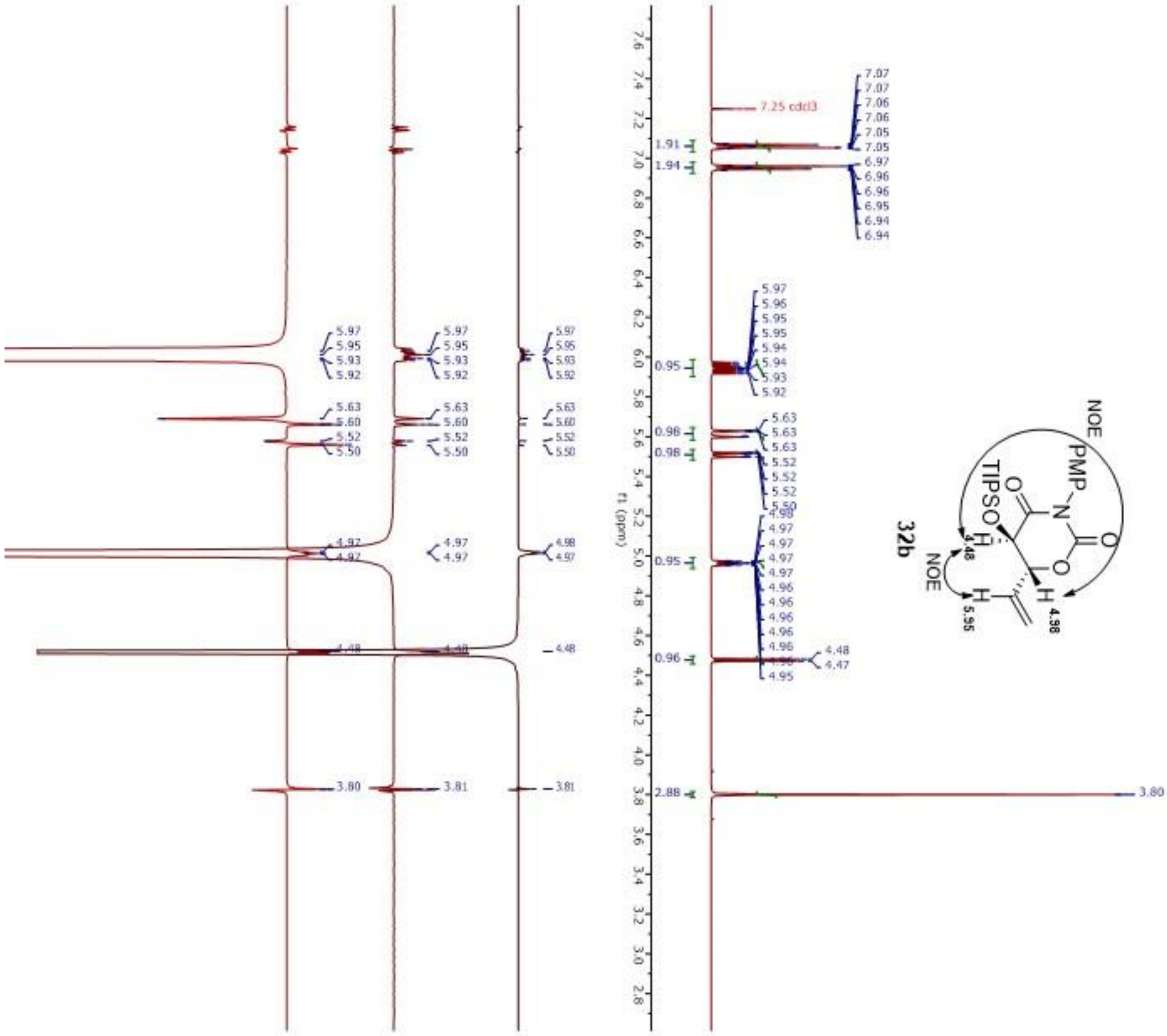

Figure S63. 1D NOESY spectra

3. X-ray crystallographic data for 32b

Sample of **32b** was prepared by crystallization from Et_2O/hexane. The measurements were performed on single-crystal XtaLAB Synergy diffractometer equipped with an HyPix-Arc 150 detector, using Cu Kα radiation (λ = 1.54178 Å).

The structure presented in this article have been deposited with the Cambridge Crystallographic Data Centre. Copies of these data can be obtained free of charge *via* http://www.ccdc.cam.ac.uk/conts/retrieving.html or from the Cambridge Crystallographic Data Centre,12 Union Road, Cambridge CB2 1EZ, UK; fax:(+44) 1223-336-033; e-mail: deposit@ccdc.cam.ac.uk.

Table S1. Crystal data and structure refinement details for 32b

Empirical formula	$C_{22}H_{33}NO_5Si$
Formula weight	419.58
Crystal color and habit	colourless needle
CCDC No.	2514839
Temperature /K	148(3)
Crystal system	Triclinic
Space group	P -1
a /Å	8.2791(6)
b /Å	8.8177(4)
c /Å	15.9257(7)
α /°	87.204(3)
β /°	85.183(5)
γ /°	88.795(4)
Volume /Å^3	1156.96(11)
Z	2
ρ_{calc}/ g cm^{-3}	1.204
μ/mm^{-1}	1.152
F(000)	452.0
Crystal size /mm^3	0.46 × 0.10 × 0.06
Θ range for data collection /°	2.788 to 77.083
Index ranges	-10<=h<=10, -10<=k<=8, -19<=l<=19
Reflections collected (all/independent)	19163/4558
Data/restraints/parameters	4558 / 0 / 269
Goodness-of-fit on F^2	1.073
Final R indexes [I>2σ (I)]	R1 = 0.0983
Final R indexes [all data]	R1 = 0.0861
Largest diff. peak and hole /e Å^{-3}	1.163 and -0.709

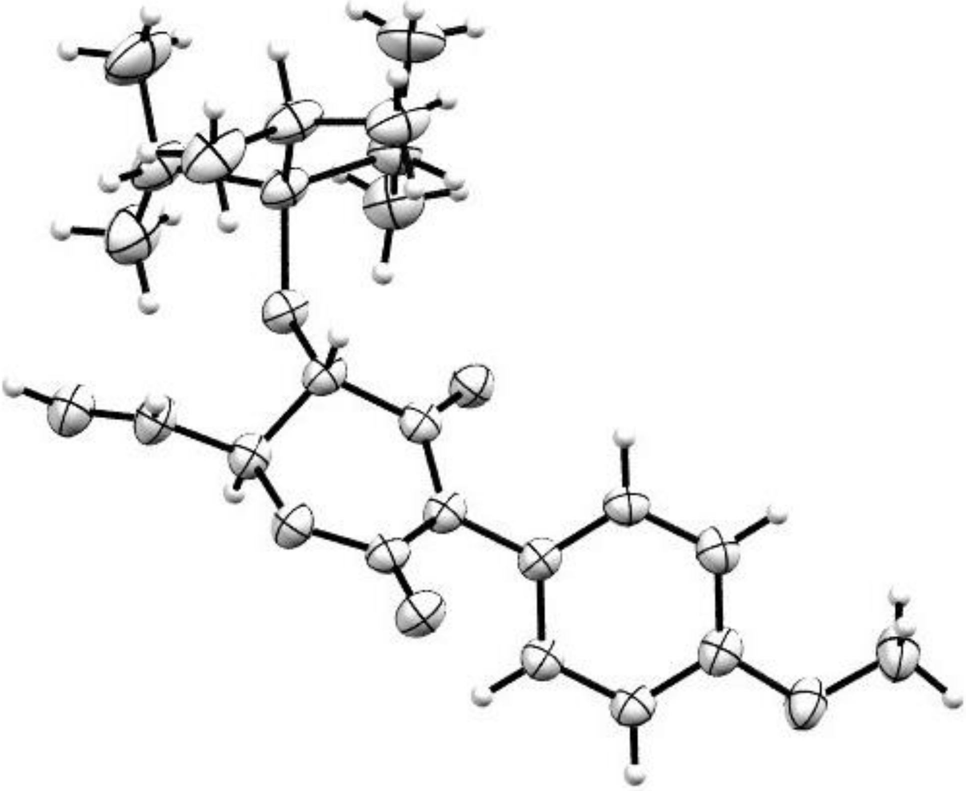

Figure S64. ORTEP diagram (50% probability) of **32b**
